# Neoadjuvant Chemoimmunotherapy for NSCLC: A Systematic Review and Meta-Analysis

**DOI:** 10.1001/jamaoncol.2024.0057

**Published:** 2024-03-21

**Authors:** Mark Sorin, Connor Prosty, Louis Ghaleb, Kathy Nie, Khaled Katergi, Muhammad H. Shahzad, Laurie-Rose Dubé, Aline Atallah, Anikka Swaby, Matthew Dankner, Trafford Crump, Logan A. Walsh, Pierre O. Fiset, Boris Sepesi, Patrick M. Forde, Tina Cascone, Mariano Provencio, Jonathan D. Spicer

**Affiliations:** 1 Rosalind and Morris Goodman Cancer Institute, McGill University, Montréal, Quebec, Canada; 2 Department of Human Genetics, McGill University, Montréal, Quebec, Canada; 3 Faculty of Medicine and Health Sciences, McGill University, Montréal, Quebec, Canada; 4 Faculty of Medicine, University of Montreal, Montréal, Quebec, Canada; 5 Department of Surgery, McGill University, Montréal, Quebec, Canada; 6 Department of Pathology, McGill University, Montréal, Quebec, Canada; 7 Department of Thoracic and Cardiovascular Surgery, University of Texas MD Anderson Cancer Center, Houston, Texas; 8 Bloomberg-Kimmel Institute for Cancer Immunotherapy, Johns Hopkins University, Baltimore, Maryland; 9 Department of Thoracic/Head and Neck Medical Oncology, University of Texas MD Anderson Cancer Center, Houston, Texas; 10 Department of Medical Oncology, Puerta de Hierro University Hospital, Autonomous University, Madrid, Instituto de Investigacion Sanitaria Puerta de Hierro–Segovia de Arana, Spain

## Abstract

**Importance:**

To date, no meta-analyses have comprehensively assessed the association of neoadjuvant chemoimmunotherapy with clinical outcomes in non–small cell lung cancer (NSCLC) in randomized and nonrandomized settings. In addition, there exists controversy concerning the efficacy of neoadjuvant chemoimmunotherapy for patients with NSCLC with programmed cell death 1 ligand 1 (PD-L1) levels less than 1%.

**Objective:**

To compare neoadjuvant chemoimmunotherapy with chemotherapy by adverse events and surgical, pathological, and efficacy outcomes using recently published randomized clinical trials and nonrandomized trials.

**Data Sources:**

MEDLINE and Embase were systematically searched from January 1, 2013, to October 25, 2023, for all clinical trials of neoadjuvant chemoimmunotherapy and chemotherapy that included at least 10 patients.

**Study Selection:**

Observational studies and trials reporting the use of neoadjuvant radiotherapy, including chemoradiotherapy, molecular targeted therapy, or immunotherapy monotherapy, were excluded.

**Main Outcomes and Measures:**

Surgical, pathological, and efficacy end points and adverse events were pooled using a random-effects meta-analysis.

**Results:**

Among 43 eligible trials comprising 5431 patients (4020 males [74.0%]; median age range, 55-70 years), there were 8 randomized clinical trials with 3387 patients. For randomized clinical trials, pooled overall survival (hazard ratio, 0.65; 95% CI, 0.54-0.79; *I*^2^ = 0%), event-free survival (hazard ratio, 0.59; 95% CI, 0.52-0.67; *I*^2^ = 14.9%), major pathological response (risk ratio, 3.42; 95% CI, 2.83-4.15; *I*^2^ = 31.2%), and complete pathological response (risk ratio, 5.52; 95% CI, 4.25-7.15; *I*^2^ = 27.4%) favored neoadjuvant chemoimmunotherapy over neoadjuvant chemotherapy. For patients with baseline tumor PD-L1 levels less than 1%, there was a significant benefit in event-free survival for neoadjuvant chemoimmunotherapy compared with chemotherapy (hazard ratio, 0.74; 95% CI, 0.62-0.89; *I*^2^ = 0%).

**Conclusion and Relevance:**

This study found that neoadjuvant chemoimmunotherapy was superior to neoadjuvant chemotherapy across surgical, pathological, and efficacy outcomes. These findings suggest that patients with resectable NSCLC with tumor PD-L1 levels less than 1% may have an event-free survival benefit with neoadjuvant chemoimmunotherapy.

## Key Points

**Question:**

Do patients with resectable non–small cell lung cancer (NSCLC) and tumor programmed cell death 1 ligand 1 (PD-L1) levels less than 1% benefit from neoadjuvant chemoimmunotherapy?

**Findings:**

In this meta-analysis of 43 studies involving 5431 patients with resectable NSCLC, there was a significant benefit in event-free survival (hazard ratio, 0.74; 95% CI, 0.62-0.89; *I*^2^ = 0%) but not overall survival for patients with baseline tumor PD-L1 levels less than 1% who received neoadjuvant chemoimmunotherapy compared with chemotherapy.

**Meaning:**

This study found that neoadjuvant chemoimmunotherapy was superior to neoadjuvant chemotherapy across surgical, pathological, and efficacy outcomes and patients with resectable NSCLC and tumor PD-L1 levels less than 1% had an event-free survival benefit with neoadjuvant chemoimmunotherapy.

## Teaser

This meta-analysis investigates the association of neoadjuvant chemoimmunotherapy with patient benefits vs neoadjuvant chemotherapy in patients with non–small cell lung cancer (NSCLC).

## Introduction

Lung cancer remains the leading cause of cancer death worldwide.^1^ Non–small cell lung cancer (NSCLC) accounts for 80% to 85% of all lung cancers and is associated with poor outcomes owing to advanced disease at diagnosis.^2^ Surgery remains the main treatment modality for early-stage NSCLC, but only one-quarter of patients have resectable disease at diagnosis and approximately 30% to 55% of patients will have a recurrence after surgery.^3,4,5^ Recently, several clinical trials have been initiated to assess the efficacy of treatment in the neoadjuvant setting. Neoadjuvant treatment can target micrometastatic disease prior to surgery and can downstage cancer, permitting resection that was previously not possible or considered too extensive.^6,7^ Potential drawbacks of this approach include progression on neoadjuvant therapy, thus precluding surgery, and possible effects on surgical performance.

In 2022, the Food and Drug Administration (FDA) approved neoadjuvant nivolumab with platinum-doublet chemotherapy for patients with resectable NSCLC based on results of the Phase III CheckMate 816 trial.^8^ This approval led to a recommendation by the National Comprehensive Cancer Network for neoadjuvant nivolumab with platinum-doublet chemotherapy in patients with stage IB to IIIA or IIIB (only T3, N2) NSCLC.^9^ In 2023, the FDA approved neoadjuvant pembrolizumab in combination with platinum-containing chemotherapy for resectable NSCLC followed by single-agent pembrolizumab in the adjuvant setting across all programmed cell death 1 ligand 1 (PD-L1) strata after overall survival (OS) results were produced from KEYNOTE-671.^10,11^ In contrast, the European Medicines Agency approved nivolumab in combination with platinum-based chemotherapy in the neoadjuvant setting only in patients at high risk of recurrence and with tumor cell PD-L1 expression levels greater than 1%. This was based on an analysis showing no difference in event-free survival (EFS) between neoadjuvant chemoimmunotherapy and chemotherapy in patients with PD-L1 levels less than 1% in CheckMate 816, with a decision still pending regarding results of KEYNOTE-671. These conflicting approvals highlight the uncertainty concerning the efficacy of neoadjuvant chemoimmunotherapy in subgroups of patients and demonstrate the need for a meta-analysis across all published neoadjuvant chemoimmunotherapy trials.

Existing meta-analyses of nonrandomized clinical trials have described inconsistent results when comparing neoadjuvant chemoimmunotherapy trials with studies assessing other neoadjuvant treatment regimens.^12,13^ This highlights that the inclusion of single-arm studies results in indirect comparisons that are prone to high levels of bias due to interstudy heterogeneity in stage, sex, age, and histology. Finally, recent randomized clinical trials (RCTs) of neoadjuvant chemoimmunotherapy are not captured by existing systematic reviews. Thus, we sought to compare neoadjuvant chemoimmunotherapy and neoadjuvant chemotherapy in a larger number of patients through a meta-analysis of RCTs and non-RCTs by assessing surgical, pathological, and efficacy outcomes, as well as treatment-related adverse events (TRAEs) and surgical adverse events (SRAEs). We also describe the performance of neoadjuvant chemoimmunotherapy in specific subgroups of patients derived from RCTs to inform future neoadjuvant drug approvals.

## Methods

### Study Protocol

This systematic review and meta-analysis followed the Preferred Reporting Items for Systematic Reviews and Meta-analyses (PRISMA) reporting guideline.^14^ The protocol of this study was registered with PROSPERO (CRD42023392998).

### Search Strategy and Selection Criteria

Due to the evolution of surgical and staging modalities, as well as an existing landmark meta-analysis on neoadjuvant chemotherapy,^15^ we decided to include studies published from 2013 onward. MEDLINE and Embase databases were searched from January 1, 2013, to the present (search last updated October 25, 2023) with no language restriction for published single-arm trials and RCTs pertaining to neoadjuvant chemoimmunotherapy, chemotherapy, or both treatments in patients with resectable lung cancer. The complete search strategy can be found in eTables 1 and 2 in Supplement 1. Only published articles reporting trial-level data related to neoadjuvant chemotherapy or neoadjuvant chemoimmunotherapy were included. Abstracts, conference proceedings, retrospective studies, editorials, comments, gray literature, and all other publication types without trial-level evidence were excluded. Clinical trials reporting the use of radiotherapy, including neoadjuvant chemoradiotherapy, molecular targeted therapy, or immunotherapy monotherapy, were excluded. Only studies with adult patients were included. Studies that included only patients with NSCLC with *EGFR* variants or studies that did not use TNM staging were excluded. All articles were screened for relevance by title and abstract by 2 independent reviewers (M.S. and C.P., L.G., K.N., K.K., M.H.S., L-.R.D., A.A., or A.S.). Relevant articles were then read fully to determine eligibility for inclusion, once again by 2 independent reviewers (M.S. and C.P., L.G., K.N., K.K., M.H.S., L-.R.D., A.A., or A.S.). In all cases, disagreements were resolved by a third reviewer (M.D.). In addition, abstracts from the 2023 American Society of Clinical Oncology conference, European Society for Medical Oncology conference, European Lung Cancer Congress, World Conference on Lung Cancer, and American Association for Cancer Research conference were screened for updates on published RCTs and for results on new RCTs. The most updated online data were included, and online material from conference websites was also used.

### Quality Assessment

Risk of bias was assessed by 2 independent reviewers (M.S. and K.N.) using the revised Cochrane risk of bias tool^16,17^ for RCTs and the Joanna Briggs Institute checklist for single-arm studies. Disagreements were resolved by a third reviewer (M.D.).

### Data Extraction

Relevant data were extracted independently by 2 researchers (M.S. and C.P., L.G., K.N., K.K., M.H.S., L-.R.D., A.A., or A.S.) from included articles using a prespecified form. Discrepancies were discussed with a third reviewer (M.D.) and were resolved through consensus. Extracted data included surgical, pathological, and efficacy outcomes, as well as adverse events (eMethods in Supplement 1). For RCTs, hazard ratios (HRs) for EFS and OS and their 95% CIs were also extracted from each included study. When available, HRs by subgroup stratified by sex, age, histology, PD-L1 level, stage, and type of platinum agent received (carboplatin vs cisplatin) were also extracted. All single arms were extracted if they met inclusion criteria. Only studies that reported the outcome of interest were included in the relevant analysis.

### Statistical Analysis

Outcomes of interest included surgical (surgical resection rate and R0 resection), pathological (major pathological response [MPR] and complete pathological response [pCR]), and efficacy (EFS and OS) outcomes, as well as adverse events (SRAEs and TRAEs) (eMethods in Supplement 1). We performed analyses using metafor^18^ and meta^19^ packages in R statistical software version 3.4.0 (R Project for Statistical Computing).

Outcome data from single-arm studies were pooled independently for neoadjuvant chemoimmunotherapy and chemotherapy by an inverse variance random-effects meta-analysis using logit transformation. Pooled proportion estimates were compared between chemotherapy and chemoimmunotherapy by univariate metaregression.^20^ For single-arm studies, the incidence of death was calculated using the number of death events and the median follow-up time, with a comparison done by subgroup analysis. For RCT data, we performed a restricted maximum likelihood meta-analysis of risk ratios (RRs) and HRs for time-dependent data. Subgroup analyses were exploratory. For each analysis, we included the 95% CIs, *I*^2^ statistic, τ^2^ statistic, and χ^2^. All tests were 2-sided, and a *P* value < .05 was considered significant unless otherwise indicated.

## Results

Our search returned 642 total and 602 unique results. After title and abstract screening, 519 studies were excluded, and an additional 41 studies were removed after full-text screening. A total of 42 publications^8,10,21,22,23,24,25,26,27,28,29,30,31,32,33,34,35,36,37,38,39,40,41,42,43,44,45,46,47,48,49,50,51,52,53,54,55,56,57,58,59,60^ and 6 abstracts^11,61,62,63,64,65^ met inclusion criteria, including 43 trials^8,10,21,22,23,24,25,26,27,29,30,31,32,33,34,35,37,38,39,40,41,42,43,44,45,46,47,48,49,50,51,52,53,54,55,56,57,58,59,60,61,62,63^ and 5 follow-up studies,^11,28,36,64,65^ from which we extracted 54 study arms; 5431 patients were included overall in the 43 trials (4020 males [74.0%]; median age range, 55-70 years) (Table; eTable 3 and eFigure 1 in Supplement 1). There were 8 RCTs comparing neoadjuvant chemoimmunotherapy with chemotherapy, namely CheckMate 816,^8,64,65,66^ KEYNOTE-671,^10,11,67^ NADIM II,^21^ AEGEAN,^41^ Neotorch,^63,68^ Checkmate 77T,^61,69^ TD-FOREKNOW,^23^ and RATIONALE 315^62,70^ (Table). Across 3387 patients (2582 males [76.2%]; median age range, 61-66 years) included in 8 RCTs,^8,10,11,21,23,41,61,62,63,64,65,66,67,68,69,70^ there were 1688 patients (49.8%) treated with chemotherapy, 1699 patients (50.2%) treated with chemoimmunotherapy, 1882 patients (55.6%) who had squamous histology, and 2443 patients (72.1%) who had stage III disease. Across all studies, there were 27 neoadjuvant chemotherapy^8,10,11,21,22,23,24,26,31,33,35,36,37,38,39,41,42,43,44,45,46,47,50,51,52,59,61,62,63,64,65,66,67,68,69,70^ and 27 neoadjuvant chemoimmunotherapy^8,10,11,21,23,25,27,28,29,30,32,34,40,41,48,49,53,54,55,56,57,58,60,61,62,63,64,65,66,67,68,69,70^ arms; 21 arms^21,22,23,24,25,26,27,28,29,30,31,32,33,34,35,36,37,49,50,63,68^ included only patients with stage III disease. Studies ranged from 10 patients^33^ to 400 patients^10^ per arm, and there were 2347 patients assigned to neoadjuvant chemoimmunotherapy and 3084 patients assigned to neoadjuvant chemotherapy. With regard to histology, 2767 patients had squamous cell carcinoma, with the remaining patients having nonsquamous histologies. For neoadjuvant chemoimmunotherapy studies,^8,10,11,21,23,25,27,28,29,30,32,34,40,41,48,49,53,54,55,56,57,58,60,61,62,63,64,65,66,67,68,69,70^ there were 533 patients treated with nivolumab, 434 patients treated with pembrolizumab, 433 patients treated with durvalumab, 381 patients treated with toripalimab, 226 patients treated with tislelizumab, 169 patients treated with sintilimab, 46 patients treated with ipilimumab, 43 patients treated with camrelizumab, 37 patients treated with adebrelimab, 30 patients treated with atezolizumab, and 15 patients treated with avelumab. For 11 studies,^8,10,11,21,23,29,38,39,41,61,62,63,64,65,66,67,68,69,70^ more than a single arm was extracted per study. For 4 studies,^8,10,27,35^ a follow-up study^11,28,36,64,65,66,67^ was found (2 were found for CheckMate 816), and the updated data were used instead of the original data.

**Table.** RCT Study Characteristics

Treatment mode	Source	ClinicalTrials.gov Identifier	Clinical trial name	Study phase	Study design	Neoadjuvant treatment regimen	Main inclusion criteria	Patients, No.	Males, %	Median age, y	SCC, %	Stage III disease, %
Chemotherapy	Forde et al,^8^ 2022; Forde et al, ^64^ 2023; Provencio Pulla et al^65^ 2023; Provencio Pulla et al^66^ 2023	NCT02998528	CheckMate 816	III	Open label, randomized	Platinum-doublet chemotherapy	Stage IB-IIIA resectable NSCLC	179	70.9	65	53.1	64.2
Chemoimmunotherapy	Forde et al,^8^ 2022; Forde et al, ^64^ 2023; Provencio Pulla et al^65^ 2023; Provencio Pulla et al^66^ 2023	NCT02998528	CheckMate 816	III	Open label, randomized	Nivolumab + platinum-doublet chemotherapy	Stage IB-IIIA resectable NSCLC	179	71.5	64	48.6	63.1
Chemotherapy	Wakelee et al,^10^ 2023; Spicer et al,^11^ 2023; Spicer et al,^67^ 2023	NCT03425643	KEYNOTE-671	III	Double blind, randomized	Platinum-doublet chemotherapy	Stage II-IIIB resectable NSCLC	400	71.0	64	43.2	69.8
Chemoimmunotherapy	Wakelee et al,^10^ 2023; Spicer et al,^11^ 2023; Spicer et al,^67^ 2023	NCT03425643	KEYNOTE-671	III	Double blind, randomized	Pembrolizumab + platinum-doublet chemotherapy	Stage II-IIIB resectable NSCLC	397	70.3	63	43.1	70.3
Chemoimmunotherapy	Heymach et al,^41^ 2023	NCT03800134	AEGEAN	III	Double blind, randomized	Durvalumab + carboplatin and paclitaxel	Stage IIA-IIIB N2 resectable NSCLC	366	68.9	65	46.2	71.3
Chemotherapy	Heymach et al,^41^ 2023	NCT03800134	AEGEAN	III	Double blind, randomized	Carboplatin and paclitaxel	Stage IIA-IIIB N2 resectable NSCLC	374	74.3	65	51.1	70.3
Chemoimmunotherapy	Provencio et al,^21^ 2023	NCT03838159	NADIMII	II	Open label, randomized	Nivolumab + paclitaxel + carboplatin	Stage IIIA-IIIB resectable NSCLC	57	63.2	65	36.8	100
Chemotherapy	Provencio et al,^21^ 2023	NCT03838159	NADIMII	II	Open label, randomized	Paclitaxel + carboplatin	Stage IIIA-IIIB resectable NSCLC	29	55.2	63	48.3	100
Chemoimmunotherapy	Cascone et al,^61^ 2023; Cascone et al,^69^ 2023	NCT04025879	Checkmate 77t	III	Double blind, randomized	Nivolumab + platinum-based chemotherapy	Stage IIA-IIIB N2 resectable NSCLC	229	72.9	66	50.7	63.8
Chemotherapy	Cascone et al,^61^ 2023; Cascone et al,^69^ 2023	NCT04025879	Checkmate 77t	III	Double blind, randomized	Platinum-based chemotherapy	Stage IIA-IIIB N2 resectable NSCLC	232	69.0	66	50.9	64.2
Chemoimmunotherapy	Lu et al,^63^ 2023; Lu et al,^68^ 2023	NCT04158440	Neotorch	III	Double blind, randomized	Toripalimab + platinum based chemotherapy	Stage II-III resectable NSCLC	202	89.6	62	77.7	100
Chemotherapy	Lu et al,^63^ 2023; Lu et al,^68^ 2023	NCT04158440	Neotorch	III	Double blind, randomized	Platinum-based chemotherapy	Stage II-III resectable NSCLC	202	93.6	61	77.7	100
Chemoimmunotherapy	Lei et al,^23^ 2023	NCT04338620	TD-FOREKNOW	II	Open label, randomized	Camrelizumab + platinum-based chemotherapy	Stage IIIA-IIIB resectable NSCLC	43	79.1	61	62.8	100
Chemotherapy	Lei et al,^23^ 2023	NCT04338620	TD-FOREKNOW	II	Open label, randomized	Platinum-based chemotherapy	Stage IIIA-IIIB resectable NSCLC	45	88.9	61	71.1	100
Chemotherapy	Yue et al,^62^ 2023; Yue et al, ^70^ 2023	NCT04379635	RATIONALE 315	III	Double blind, randomized	Platinum-based chemotherapy	Stage II-IIIA resectable NSCLC	227	90.3	63	77.1	58.1
Chemoimmunotherapy	Yue et al,^62^ 2023; Yue et al, ^70^ 2023	NCT04379635	RATIONALE 315	III	Double blind, randomized	Tislelizumab + platinum-based chemotherapy	Stage II-IIIA resectable NSCLC	226	90.7	62	79.2	58.4

Abbreviations: NSCLC, non–small cell lung cancer; RCT, randomized clinical trial; SCC, squamous cell carcinoma of the lung.

Overall, there was a low risk of bias among RCTs, with bias arising due to unbalanced treatment arms in 1 study^23^ and potentially multiple analyses of the same data in another study^41^ (eFigure 2 in Supplement 1). For nonrandomized studies,^22,24,25,26,27,28,29,30,31,32,33,34,35,36,37,38,39,40,42,43,44,45,46,47,48,49,50,51,52,53,54,55,56,57,58,59,60^ bias concerns were mostly associated with inadequate length of follow-up (eTable 4 in Supplement 1).

**Figure 1.**
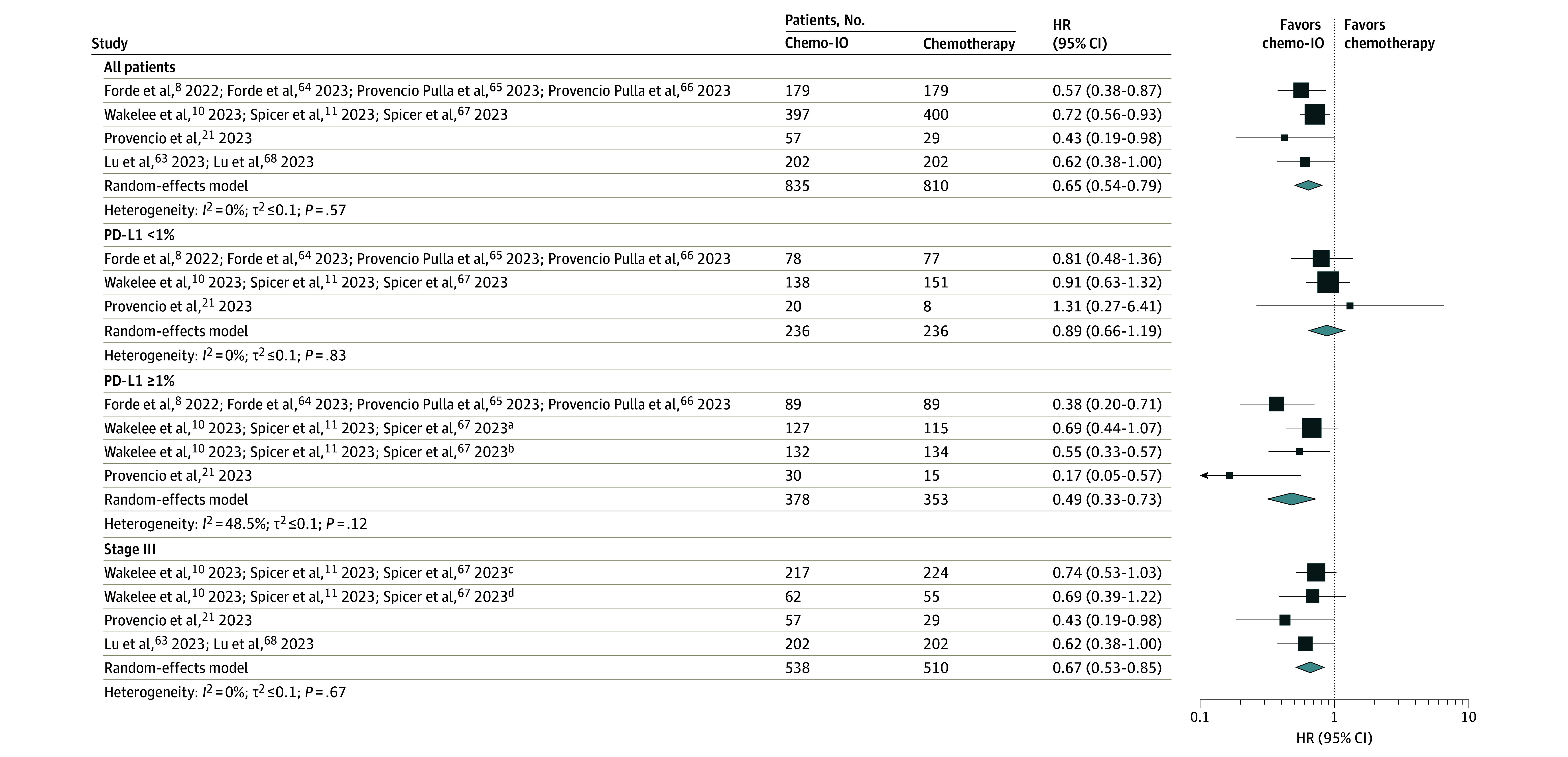
Pooled Hazard Ratios (HRs) of Overall Survival Across Randomized Clinical Trials Pooled HRs comparing neoadjuvant chemoimmunotherapy with neoadjuvant chemotherapy are given for all patients, by programmed cell death 1 ligand 1 (PD-L1) status, and for patients with stage III disease. For Wakelee et al,^10^ Spicer et al,^11^ and Spicer et al,^67^ PD-L1 levels of 1% or greater and stage III were each split into 2 groups. Chemo-IO indicates chemoimmunotherapy. ^a^ PD-L1 group: 1% to 49%. ^b^ PD-L1 group 50% or greater. ^c^Stage IIIA. ^d^Stage IIIB.

**Figure 2.**
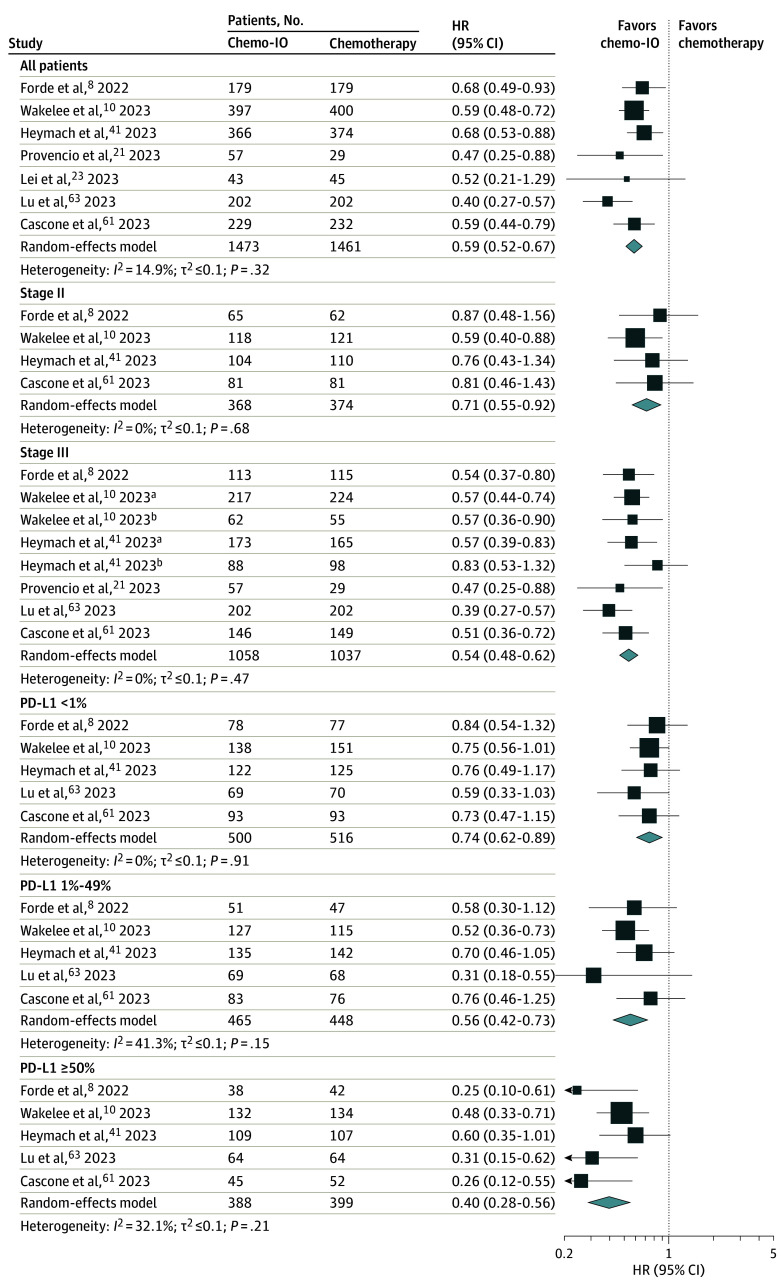
Pooled Hazard Ratios (HRs) of Event-Free Survival Across Randomized Clinical Trials Pooled HRs comparing neoadjuvant chemoimmunotherapy with neoadjuvant chemotherapy are given for all patients, by stage, and by programmed cell death 1 ligand 1 (PD-L1) level. Additional references include Forde et al,^64^ Provencio Pulla et al,^65^ and Provencio Pulla et al^66^ for Forde et al^8^; Spicer et al^11^ and Spicer et al^67^ for Wakelee et al^10^; Lu et al^68^ for Lu et al ^63^; and Cascone et al^69^ for Cascone et al.^61^ For Forde et al,^8^ 97.38% CIs were reported for overall event-free survival. For Heymach et al,^41^ a stratified HR was reported for overall event-free-survival. For Wakelee et al^10^ and Heymach et al,^41^ stage III was split into 2 groups. For Provencio,^21^ progression-free survival was used instead of event-free survival. Chemo-IO indicates chemoimmunotherapy. ^a^Stage IIIA. ^b^Stage IIIB.

Across RCTs, the pooled OS favored neoadjuvant chemoimmunotherapy (HR, 0.65; 95% CI, 0.54-0.79; *I*^2^ = 0%) over neoadjuvant chemotherapy (Figure 1; eTable 5 in Supplement 1).^8,10,11,21,23,41,61,62,63,64,65,66,67,68,69,70^ While there was an improvement in OS for patients treated with neoadjuvant chemoimmunotherapy vs neoadjuvant chemotherapy among patients with tumor PD-L1 levels of 1% or greater (HR, 0.49; 95% CI, 0.33-0.73; *I*^2^ = 48.5%) and patients with stage III cancer (HR, 0.67; 95% CI, 0.53-0.85; *I*^2^ = 0%), this was not seen for patients with tumor PD-L1 levels less than 1% (HR, 0.89; 95% CI, 0.66-1.19; *I*^2^ = 0%) (Figure 1). The pooled EFS estimate across all patients in RCTs favored neoadjuvant chemoimmunotherapy over neoadjuvant chemotherapy (HR, 0.59; 95% CI, 0.52-0.67; *I*^2^ = 14.9%) (Figure 2; eTable 6 in Supplement 1).^8,10,11,21,23,41,61,62,63,64,65,66,67,68,69,70^ For individual subgroups, we found an improvement in EFS for neoadjuvant chemoimmunotherapy over neoadjuvant chemotherapy across patients treated in Europe (HR, 0.65; 95% CI, 0.50-0.83; *I*^2^ = 0%) and Asia (HR, 0.51; 95% CI, 0.41-0.63; *I*^2^ = 8.2%) but not North America (HR, 0.69; 95% CI, 0.43-1.10; *I*^2^ = 0%) (eFigure 3 in Supplement 1). There was also an improvement with neoadjuvant chemoimmunotherapy vs neoadjuvant chemotherapy for patients aged younger than 65 years and 65 years or older (eFigure 4 in Supplement 1), females and males (eFigure 5 in Supplement 1), patients with squamous and nonsquamous histologies (eFigure 6 in Supplement 1). A significant improvement in EFS with neoadjuvant chemoimmunotherapy vs neoadjuvant chemotherapy was seen for patients with stage II (HR, 0.71; 95% CI, 0.55-0.92; *I*^2^ = 0%) and stage III (HR, 0.54; 95% CI, 0.48-0.62; *I*^2^ = 0%) cancer (Figure 2). For patients with baseline tumor PD-L1 levels less than 1%, the pooled outcome favored neoadjuvant chemoimmunotherapy (HR, 0.74; 95% CI, 0.62-0.89; *I*^2^ = 0%) (Figure 2). There was significant improvement for patients with PD-L1 levels of 1% to 49% (HR, 0.56; 95% CI, 0.42-0.73; *I*^2^ = 41.3%) and 50% or greater (HR, 0.40; 95% CI, 0.28-0.56; *I*^2^ = 32.1%) (Figure 2). By type of platinum therapy used, there was an EFS benefit for patients treated with cisplatin and carboplatin in the neoadjuvant chemoimmunotherapy group (eFigure 7 in Supplement 1). There was an improvement across all subgroups for MPR and pCR with neoadjuvant chemoimmunotherapy vs neoadjuvant chemotherapy (eFigures 8-9 in Supplement 1), with the exception of MPR for patients treated in North America.

**Figure 3.**
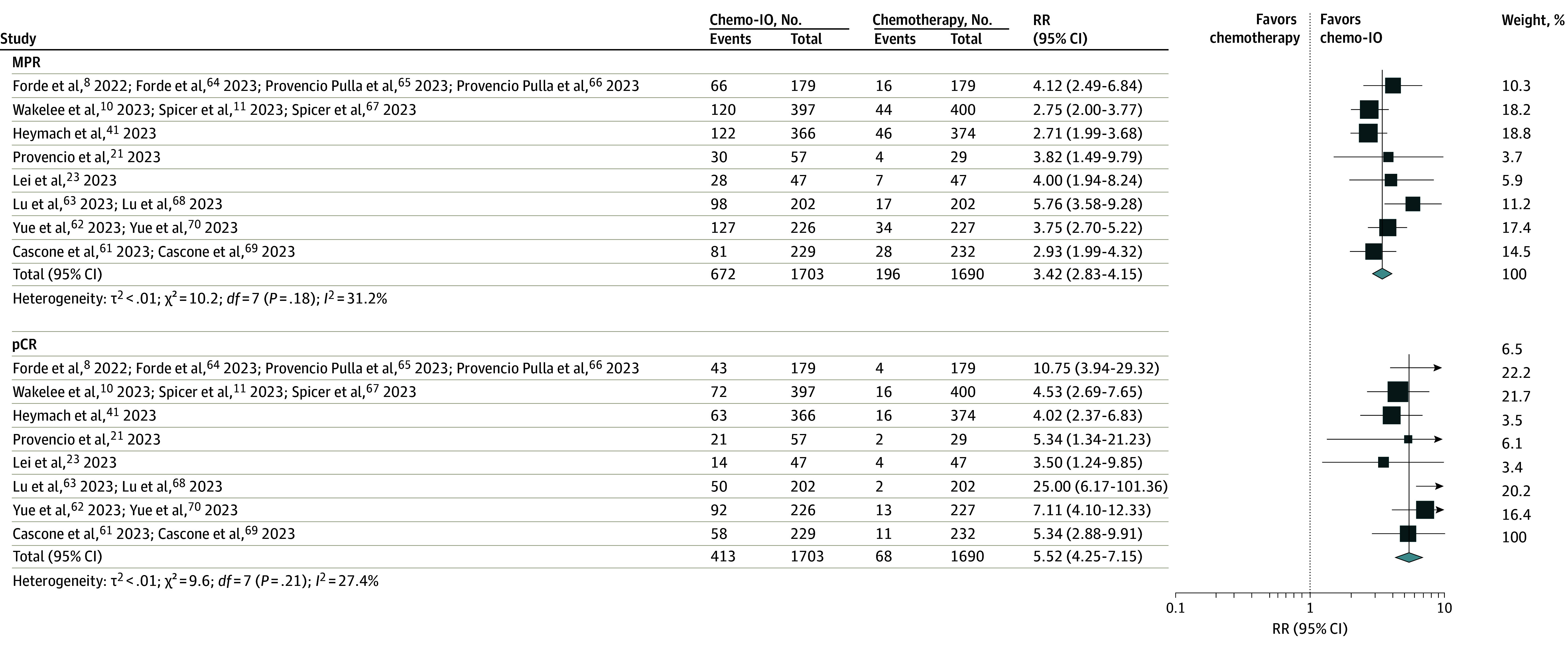
Pooled Risk Ratios (RRs) for Pathological Outcomes Across Randomized Clinical Trials Pooled RRs comparing neoadjuvant chemoimmunotherapy with neoadjuvant chemotherapy are given for major pathological response (MPR) and complete pathological response (pCR). Chemo-IO indicates chemoimmunotherapy.

**Figure 4.**
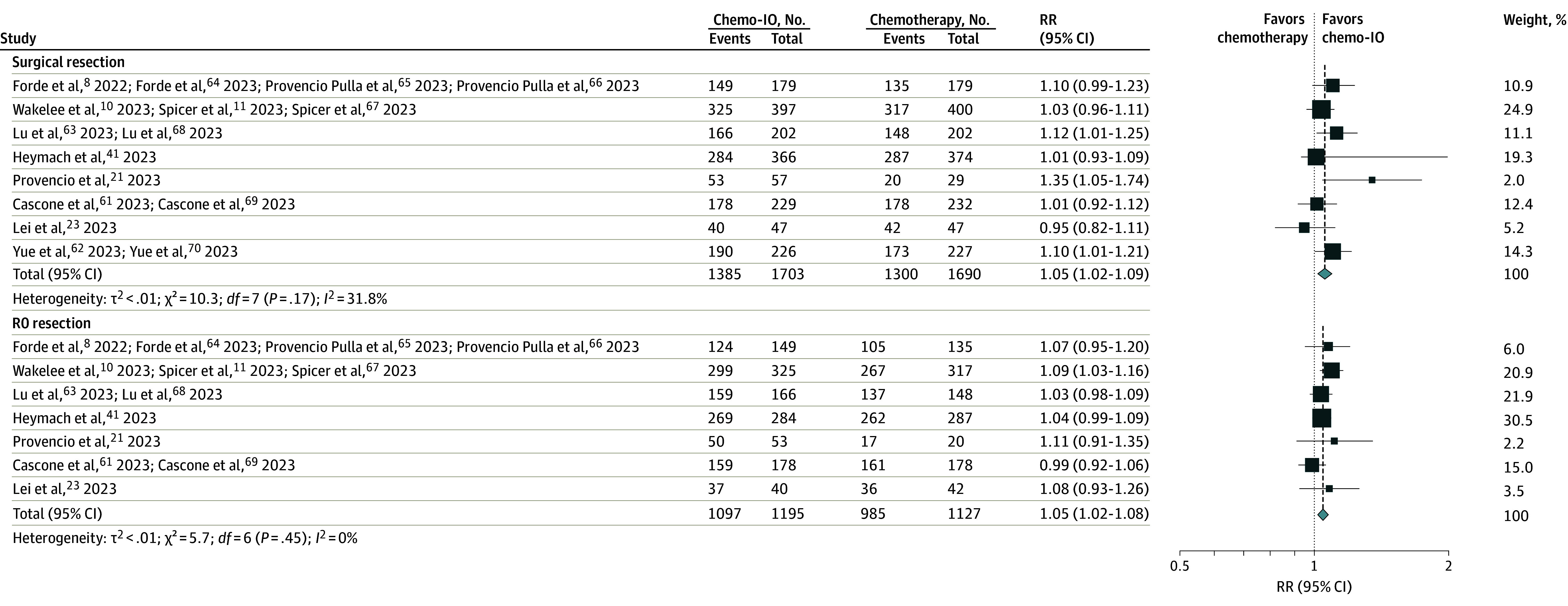
Pooled Risk Ratios (RRs) for Surgical Outcomes Across Randomized Clinical Trials Pooled RRs comparing neoadjuvant chemoimmunotherapy with neoadjuvant chemotherapy are given for surgical resection (A) and R0 resection (B). Chemo-IO indicates chemoimmunotherapy.

For RCT pathological end points, the relative risk for MPR (RR, 3.42; 95% CI, 2.83-4.15; *I*^2^ = 31.2%), and pCR (RR, 5.52; 95% CI, 4.25-7.15; *I*^2^ = 27.4%) was significantly increased for neoadjuvant chemoimmunotherapy vs neoadjuvant chemotherapy (Figure 3).^8,10,11,21,23,41,61,62,63,64,65,66,67,68,69,70^ There was an increased relative risk of undergoing surgery (RR, 1.05; 95% CI, 1.02-1.09; *I*^2^ = 31.8%) (Figure 4)^8,10,11,21,23,41,61,62,63,64,65,66,67,68,69,70^ and lobectomy (RR, 1.07; 95% CI, 1.01-1.13; *I*^2^ = 42.4%) for neoadjuvant chemoimmunotherapy compared with chemotherapy in RCTs (eFigure 10 in Supplement 1). There was no significant difference between neoadjuvant chemotherapy and neoadjuvant chemoimmunotherapy in the relative risk of receiving all cycles of neoadjuvant therapy, pneumonectomy, or bilobectomy or having surgical delay or open surgery (eFigures 11-15 in Supplement 1). However, patients treated with neoadjuvant chemoimmunotherapy were more likely to undergo R0 resection (RR, 1.05; 95% CI, 1.02-1.08; *I*^2^ = 0%) (Figure 4). The proportion of patients in RCTs who were not resected in chemoimmunotherapy arms ranged from 4 of 57 patients (7.0%)^21^ to 51 of 229 patients (22.3%).^61,69^ Among these patients, the reason precluding surgery was patient refusal in 1 of 57 patients (1.0%)^21^ to 18 of 202 patients (8.9%)^63,68^ and progression on therapy for 0 of 57 patients^21^ to 27 of 366 patients (7.4%),^4^^1^ among other reasons (eTable 7 in Supplement 1).

There were no significant differences in the relative risk for grade 3 to 4, grade 5, or total TRAEs or SRAEs among patients receiving neoadjuvant chemoimmunotherapy vs neoadjuvant chemotherapy in RCTs (eFigures 16-21 in Supplement 1). Among complications preventing surgery (eTable 7 in Supplement 1), there was a reduced risk of not undergoing surgery (RR, 0.81; 95% CI, 0.70-0.94; *I*^2^ = 40.5%) (eFigure 22 in Supplement 1) and progression precluding surgery (RR, 0.51; 95% CI, 0.33-0.79; *I*^2^ = 49.0%) (eFigure 23 in Supplement 1), an increased risk of adverse events (RR, 2.16; 95% CI, 1.15-4.06; *I*^2^ = 0%) (eFigure 24 in Supplement 1), and no difference in risk of patient refusal (RR, 0.78; 95% CI, 0.58-1.05; *I*^2^ = 0%) (eFigure 25 in Supplement 1) or other reasons precluding surgery (RR, 0.82; 95% CI, 0.54-1.23; *I*^2^ = 16.5%) for the neoadjuvant chemoimmunotherapy vs neoadjuvant chemotherapy arm (eFigure 26 in Supplement 1).

We also compared these clinical variables across all trials, including RCTs and nonrandomized single-arm trials.^8,10,11,21,22,23,24,25,26,27,28,29,30,31,32,33,34,35,36,37,38,39,40,41,42,43,44,45,46,47,48,49,50,51,52,53,54,55,56,57,58,59,60,61,62,63,64,65,66,67,68,69,70^ For single-arm studies,^8,10,11,21,22,23,24,25,26,27,28,29,30,31,32,33,34,35,36,37,38,39,40,41,42,43,44,45,46,47,48,49,50,51,52,53,54,55,56,57,58,59,60,61,62,63,64,65,66,67,68,69,70^ there was also a significantly higher MPR and pCR for chemoimmunotherapy than chemotherapy (eFigures 27-28 in Supplement 1). There was no significant difference in the pooled prevalence of patients who had a complete response or progressive disease, but there was an increase in the proportion of patients with a partial response or stable disease for chemoimmunotherapy vs chemotherapy (eFigures 29-32 in Supplement 1).

For single-arm studies,^8,10,11,21,22,23,24,25,26,27,28,29,30,31,32,33,34,35,36,37,38,39,40,41,42,43,44,45,46,47,48,49,50,51,52,53,54,55,56,57,58,59,60,61,62,63,64,65,66,67,68,69,70^ no trend was observed for surgical resection (eFigure 33 in Supplement 1). There was a significant increase in the prevalence of R0 resections (eFigure 34 in Supplement 1) and lobectomies (eFigure 35 in Supplement 1) and a decrease in pneumonectomies for chemoimmunotherapy vs chemotherapy (eFigure 36 in the Supplement). No differences were found for the incidence of death between for chemoimmunotherapy vs chemotherapy (eFigure 37 in the Supplement).

## Discussion

To our knowledge, this is the most comprehensive meta-analysis to date comparing neoadjuvant chemoimmunotherapy and chemotherapy using RCT data. Overall, we found that neoadjuvant chemoimmunotherapy was associated with improved OS, EFS, MPR, and pCR compared with neoadjuvant chemotherapy; in addition, chemoimmunotherapy was associated with improved resectability and an increased rate of R0 resections, with a similar rate of adverse events.

Our meta-analysis showed an improvement in EFS with neoadjuvant chemoimmunotherapy over neoadjuvant chemotherapy across age (≥65 and <65 years), sex (male and female), and histology (squamous and nonsquamous cancer) groups. There was an improvement for patients with stage II and stage III disease. Chemoimmunotherapy was associated with a benefit in EFS for all 3 categories for PD-L1 level (<1%, 1%-49%, and ≥50%), although the OS benefit was restricted to the subgroup with a PD-L1 level of  1% or greater based on the current maturity of OS data. This highlights that the restriction in the approval of neoadjuvant chemoimmunotherapy exclusively for patients with a PD-L1 level of 1% or greater by the European Medicines Agency was based on a subgroup analysis from a single trial (CheckMate 816)^8^ and that the available evidence now suggests that this patient population may have an EFS benefit with neoadjuvant chemoimmunotherapy. It will be important to assess whether this translates into an OS benefit as the number of studies reporting OS and the follow-up time for these studies increase.

A major concern of the neoadjuvant approach is progression on therapy preventing surgery.^71^ We found that neoadjuvant chemoimmunotherapy was associated with a reduced risk of not undergoing surgery compared with chemotherapy alone owing to a reduced risk of progression precluding surgery; however, there was an increased risk in adverse events precluding surgery. Across all RCTs,^8,10,11,21,23,41,61,62,63,64,65,66,67,68,69,70^ 7.0% to 22.3% of patients were not resected in chemoimmunotherapy arms. Patient refusal was the reason precluding surgery in 1.0% to 8.9% of patients receiving chemoimmunotherapy and progression on therapy for 0% to 7.4% of these patients. While it is possible that patients who progressed on therapy could have benefited from up-front surgery, these patients were likely to develop early metastasis even if resected up front. There remains a clear gap in our understanding of the relative benefits associated with neoadjuvant vs adjuvant strategies for this patient population, although significant challenges exist around the feasibility of trials aimed at resolving this open question. Certainly, neoadjuvant chemoimmunotherapy may also be associated with downstaging of disease prior to surgery and a reduced extent of resection required for curative surgery, while an adjuvant strategy does not offer such an opportunity.^71^ Conversely, data in this study on resectability suggest that concerns that neoadjuvant chemoimmunotherapy may be associated with poor surgical outcomes compared with neoadjuvant chemotherapy may not be relevant. It is worth noting that results from the General Thoracic Surgery Database indicate that 30- and 90-day mortality after neoadjuvant chemotherapy are not different from outcomes of patients undergoing up-front surgery after risk adjustment.^72^ Ideally, a meta-analysis of surgical outcomes from adjuvant studies compared with those from neoadjuvant studies may help more definitively resolve this important question if a pure neoadjuvant vs adjuvant trial fails to emerge.

### Limitations

This study has several limitations. Meta-analyses of nonrandomized clinical trials are subject to a high level of bias owing to the inherent nature of indirect comparisons, which assume that trial designs and patient populations of individual arms are similar enough to be compared. Our subanalysis of nonrandomized studies may thus also be subject to confounding. However, our meta-analysis also included large RCTs comparing neoadjuvant chemoimmunotherapy with chemotherapy, which represents an important strength. Other limitations of our study include the variability in definitions for pathological and efficacy end points and differences in follow-up time. Additionally, differences in inclusion criteria may affect outcomes of individual studies, and we are not able to rule out the possibility of multiplicity and a type I error based on the large number of end points tested. Furthermore, studies varied in the number of neoadjuvant cycles, type of immunotherapy drugs (pembrolizumab, nivolumab, or durvalumab) used in combination with chemotherapy, and dose and nature of the adjuvant treatment, which could be associated with EFS and OS. Importantly, the appropriate adjuvant treatment regimen for patients treated with neoadjuvant chemoimmunotherapy remains controversial, and there is currently limited evidence to suggest the superiority of a perioperative approach compared with a pure neoadjuvant approach. However, all RCTs presented in this study had the commonality of a neoadjuvant chemoimmunotherapy approach. In terms of number of cycles, the neoSCORE trial^40^ compared 2 vs 3 cycles of neoadjuvant chemoimmunotherapy and found that at 12 months, the OS rate was 92.3% in the 2-cycle arm and 86.2% in the 3-cycle arm. These findings potentially suggest that the number of neoadjuvant cycles is associated with outcomes.

## Conclusions

This meta-analysis found that neoadjuvant chemoimmunotherapy was superior to neoadjuvant chemotherapy given that it was associated with improved efficacy (EFS and OS) and pathological (MPR and pCR) outcomes and increased surgical resection rate and R0 resections, without an increase in the rate of SRAEs or TRAEs. In addition, neoadjuvant chemoimmunotherapy was superior across sex, age, histology, and PD-L1 levels in EFS. This finding has increased importance given the recent restriction by the EMA of neoadjuvant chemoimmunotherapy to patients with PD-L1 levels of 1% or greater. Future studies should continue to assess the benefit associated with neoadjuvant chemoimmunotherapy by subgroup as OS matures. In addition, future studies may be able to assess whether the specific type of chemotherapy or immunotherapy is associated with outcomes for patients treated with neoadjuvant chemoimmunotherapy.

## Notes

Supplement 1. **eMethods.** Methodology **eFigure 1.** Study Flowchart **eFigure 2.** Randomized Clinical Trial Risk of Bias Assessment **eFigure 3.** Pooled Hazard Ratios of Event-Free Survival Across Randomized Clinical Trials Comparing Neoadjuvant Chemoimmunotherapy With Neoadjuvant Chemotherapy by Geographic Region **eFigure 4.** Pooled Hazard Ratios of Event-Free Survival Across Randomized Clinical Trials Comparing Neoadjuvant Chemoimmunotherapy With Neoadjuvant Chemotherapy by Age **eFigure 5.** Pooled Hazard Ratios of Event-Free Survival Across Randomized Clinical Trials Comparing Neoadjuvant Chemoimmunotherapy With Neoadjuvant Chemotherapy by Sex **eFigure 6.** Pooled Hazard Ratios of Event-Free Survival Across Randomized Clinical Trials Comparing Neoadjuvant Chemoimmunotherapy With Neoadjuvant Chemotherapy by Histology **eFigure 7.** Pooled Hazard Ratios of Event-Free Survival Across Randomized Clinical Trials Comparing Neoadjuvant Chemoimmunotherapy With Neoadjuvant Chemotherapy by Chemotherapy Type **eFigure 8.** Pooled Risk Ratios of Major Pathological Response Across Randomized Clinical Trials Comparing Neoadjuvant Chemoimmunotherapy With Neoadjuvant Chemotherapy by Sex, Geographic Region, Histology, Stage, Programmed Cell Death 1 Ligand 1 Status and Chemotherapy Type **eFigure 9.** Pooled Risk Ratios of Complete Pathological Response Across Randomized Clinical Trials Comparing Neoadjuvant Chemoimmunotherapy With Neoadjuvant Chemotherapy by Sex, Geographic Region, Histology, Stage, Programmed Cell Death 1 Ligand 1 Status and Chemotherapy Type **eFigure 10.** Pooled Risk Ratios Across Randomized Clinical Trials Comparing Neoadjuvant Chemoimmunotherapy With Neoadjuvant Chemotherapy by Number of Patients Who Received a Lobectomy **eFigure 11.** Pooled Risk Ratios Across Randomized Clinical Trials Comparing Neoadjuvant Chemoimmunotherapy With Neoadjuvant Chemotherapy by Number of Patients Who Received All Cycles of Neoadjuvant Treatment **eFigure 12.** Pooled Risk Ratios Across Randomized Clinical Trials Comparing Neoadjuvant Chemoimmunotherapy With Neoadjuvant Chemotherapy by Number of Patients Who Received a Pneumonectomy **eFigure 13.** Pooled Risk Ratios Across Randomized Clinical Trials Comparing Neoadjuvant Chemoimmunotherapy With Neoadjuvant Chemotherapy by Number of Patients Who Received a Bilobectomy **eFigure 14.** Pooled Risk Ratios Across Randomized Clinical Trials Comparing Neoadjuvant Chemoimmunotherapy With Neoadjuvant Chemotherapy by Number of Patients Who Had Surgical Delay **eFigure 15.** Pooled Risk Ratios Across Randomized Clinical Trials Comparing Neoadjuvant Chemoimmunotherapy With Neoadjuvant Chemotherapy by Number of Patients Who Had Open Surgery **eFigure 16.** Pooled Risk Ratios Across Randomized Clinical Trials Comparing Neoadjuvant Chemoimmunotherapy With Neoadjuvant Chemotherapy by Number of Patients Who Had Grade 3-4 Treatment-Related Adverse Events **eFigure 17.** Pooled Risk Ratios Across Randomized Clinical Trials Comparing Neoadjuvant Chemoimmunotherapy With Neoadjuvant Chemotherapy by Number of Patients Who Had Grade 5 Treatment-Related Adverse Events **eFigure 18.** Pooled Risk Ratios Across Randomized Clinical Trials Comparing Neoadjuvant Chemoimmunotherapy With Neoadjuvant Chemotherapy by Number of Patients Who Had Treatment-Related Adverse Events **eFigure 19.** Pooled Risk Ratios Across Randomized Clinical Trials Comparing Neoadjuvant Chemoimmunotherapy With Neoadjuvant Chemotherapy by Number of Patients Who Had Grade 3-4 Surgery-Related Adverse Events **eFigure 20.** Pooled Risk Ratios Across Randomized Clinical Trials Comparing Neoadjuvant Chemoimmunotherapy With Neoadjuvant Chemotherapy by Number of Patients Who Had Grade 5 Surgery-Related Adverse Events **eFigure 21.** Pooled Risk Ratios Across Randomized Clinical Trials Comparing Neoadjuvant Chemoimmunotherapy With Neoadjuvant Chemotherapy by Number of Patients Who Had Surgery-Related Adverse Events **eFigure 22.** Pooled Risk Ratios Across Randomized Clinical Trials Comparing Neoadjuvant Chemoimmunotherapy With Neoadjuvant Chemotherapy by Number of Patients Who Did Not Receive Surgery **eFigure 23.** Pooled Risk Ratios Across Randomized Clinical Trials Comparing Neoadjuvant Chemoimmunotherapy With Neoadjuvant Chemotherapy by Number of Patients Who Had Progression Precluding Surgery **eFigure 24.** Pooled Risk Ratios Across Randomized Clinical Trials Comparing Neoadjuvant Chemoimmunotherapy With Neoadjuvant Chemotherapy by Number of Patients Who Had Adverse Events Precluding Surgery **eFigure 25.** Pooled Risk Ratios Across Randomized Clinical Trials Comparing Neoadjuvant Chemoimmunotherapy With Neoadjuvant Chemotherapy by Number of Patients With Refusal Precluding Surgery **eFigure 26.** Pooled Risk Ratios Across Randomized Clinical Trials Comparing Neoadjuvant Chemoimmunotherapy With Neoadjuvant Chemotherapy by Number of Patients With Other Reasons Precluding Surgery **eFigure 27.** Pooled Proportions Across All Studies Comparing Neoadjuvant Chemoimmunotherapy With Neoadjuvant Chemotherapy by Major Pathological Response **eFigure 28.** Pooled Proportions Across All Studies Comparing Neoadjuvant Chemoimmunotherapy With Neoadjuvant Chemotherapy by Complete Pathological Response **eFigure 29.** Pooled Proportions Across All Studies Comparing Neoadjuvant Chemoimmunotherapy With Neoadjuvant Chemotherapy by Complete Response per RECIST **eFigure 30.** Pooled Proportions Across All Studies Comparing Neoadjuvant Chemoimmunotherapy With Neoadjuvant Chemotherapy by Partial Response per RECIST **eFigure 31.** Pooled Proportions Across All Studies Comparing Neoadjuvant Chemoimmunotherapy With Neoadjuvant Chemotherapy by Stable Disease per RECIST **eFigure 32.** Pooled Proportions Across All Studies Comparing Neoadjuvant Chemoimmunotherapy With Neoadjuvant Chemotherapy by Progressive Disease per RECIST. **eFigure 33.** Pooled Proportions Across All Studies Comparing Neoadjuvant Chemoimmunotherapy With Neoadjuvant Chemotherapy by Surgical Resection **eFigure 34.** Pooled Proportions Across All Studies Comparing Neoadjuvant Chemoimmunotherapy With Neoadjuvant Chemotherapy by the Number of Patients Who Had R0 Resection **eFigure 35.** Pooled Proportions Across All Studies Comparing Neoadjuvant Chemoimmunotherapy With Neoadjuvant Chemotherapy by the Number of Patients Who Had a Lobectomy **eFigure 36.** Pooled Proportions Across All Studies Comparing Neoadjuvant Chemoimmunotherapy With Neoadjuvant Chemotherapy by the Number of Patients Who Had a Pneumonectomy **eFigure 37.** Pooled Proportions Across All Studies Comparing Neoadjuvant Chemoimmunotherapy With Neoadjuvant Chemotherapy by the Incidence of Death Events **eTable 1.** Search Strategy for Embase. **eTable 2.** Search Strategy for MEDLINE. **eTable 3.** Nonrandomized Study Characteristics **eTable 4.** Joanna Briggs Bias Assessment **eTable 5.** Surgical and Efficacy Outcomes for Randomized Clinical Trials **eTable 6.** Definition of Event-Free Survival, Progression-Free Survival and Criteria Used for Pathological End Points **eTable 7.** Reasons Precluding Surgery

Supplement 2.
Data Sharing Statement
