# Supplementary material for: Neoadjuvant Chemoimmunotherapy for NSCLC: A Systematic Review and Meta-Analysis
Source: JAMA Oncol. 2024 Mar 21;10(5):621–33. doi: 10.1001/jamaoncol.2024.0057 (PMC10958389; doi:10.1001/jamaoncol.2024.0057)
Supplement: Supplement 1. — eMethods. Methodology eFigure 1. Study Flowchart eFigure 2. Randomized Clinical Trial Risk of Bias Assessment eFigure 3. Pooled Hazard Ratios of Event-Free Survival Across Randomized Clinical Trials Comparing Neoadjuvant Chemoimmunotherapy With Neoadjuvant Chemotherapy by Geographic Region eFigure 4. Pooled Hazard Ratios of Event-Free Survival Across Randomized Clinical Trials Comparing Neoadjuvant Chemoimmunotherapy With Neoadjuvant Chemotherapy by Age eFigure 5. Pooled Hazard Ratios of Event-Free Survival Across Randomized Clinical Trials Comparing Neoadjuvant Chemoimmunotherapy With Neoadjuvant Chemotherapy by Sex eFigure 6. Pooled Hazard Ratios of Event-Free Survival Across Randomized Clinical Trials Comparing Neoadjuvant Chemoimmunotherapy With Neoadjuvant Chemotherapy by Histology eFigure 7. Pooled Hazard Ratios of Event-Free Survival Across Randomized Clinical Trials Comparing Neoadjuvant Chemoimmunotherapy With Neoadjuvant Chemotherapy by Chemotherapy Type eFigure 8. Pooled Risk Ratios of Major Pathological Response Across Randomized Clinical Trials Comparing Neoadjuvant Chemoimmunotherapy With Neoadjuvant Chemotherapy by Sex, Geographic Region, Histology, Stage, Programmed Cell Death 1 Ligand 1 Status and Chemotherapy Type eFigure 9. Pooled Risk Ratios of Complete Pathological Response Across Randomized Clinical Trials Comparing Neoadjuvant Chemoimmunotherapy With Neoadjuvant Chemotherapy by Sex, Geographic Region, Histology, Stage, Programmed Cell Death 1 Ligand 1 Status and Chemotherapy Type eFigure 10. Pooled Risk Ratios Across Randomized Clinical Trials Comparing Neoadjuvant Chemoimmunotherapy With Neoadjuvant Chemotherapy by Number of Patients Who Received a Lobectomy eFigure 11. Pooled Risk Ratios Across Randomized Clinical Trials Comparing Neoadjuvant Chemoimmunotherapy With Neoadjuvant Chemotherapy by Number of Patients Who Received All Cycles of Neoadjuvant Treatment eFigure 12. Pooled Risk Ratios Across Randomized Clinical Trials Comparing Neoadju [file jamaoncol-e240057-s001.pdf]

Supplemental Online Content

Sorin M, Prosty C, Ghaleb L, et al. Neoadjuvant chemoimmunotherapy for NSCLC: a systematic review and meta-analysis. *JAMA Oncol*. Published online March 21, 2024. doi:10.1001/jamaoncol.2024.0057

eMethods. Methodology

eFigure 1. Study Flowchart

eFigure 2. Randomized Clinical Trial Risk of Bias Assessment

eFigure 3. Pooled Hazard Ratios of Event-Free Survival Across Randomized Clinical Trials Comparing Neoadjuvant Chemoimmunotherapy With Neoadjuvant Chemotherapy by Geographic Region

eFigure 4. Pooled Hazard Ratios of Event-Free Survival Across Randomized Clinical Trials Comparing Neoadjuvant Chemoimmunotherapy With Neoadjuvant Chemotherapy by Age

eFigure 5. Pooled Hazard Ratios of Event-Free Survival Across Randomized Clinical Trials Comparing Neoadjuvant Chemoimmunotherapy With Neoadjuvant Chemotherapy by Sex

eFigure 6. Pooled Hazard Ratios of Event-Free Survival Across Randomized Clinical Trials Comparing Neoadjuvant Chemoimmunotherapy With Neoadjuvant Chemotherapy by Histology

eFigure 7. Pooled Hazard Ratios of Event-Free Survival Across Randomized Clinical Trials Comparing Neoadjuvant Chemoimmunotherapy With Neoadjuvant Chemotherapy by Chemotherapy Type

eFigure 8. Pooled Risk Ratios of Major Pathological Response Across Randomized Clinical Trials Comparing Neoadjuvant Chemoimmunotherapy With Neoadjuvant Chemotherapy by Sex, Geographic Region, Histology, Stage, Programmed Cell Death 1 Ligand 1 Status and Chemotherapy Type

eFigure 9. Pooled Risk Ratios of Complete Pathological Response Across Randomized Clinical Trials Comparing Neoadjuvant Chemoimmunotherapy With Neoadjuvant Chemotherapy by Sex, Geographic Region, Histology, Stage, Programmed Cell Death 1 Ligand 1 Status and Chemotherapy Type

eFigure 10. Pooled Risk Ratios Across Randomized Clinical Trials Comparing Neoadjuvant Chemoimmunotherapy With Neoadjuvant Chemotherapy by Number of Patients Who Received a Lobectomy

© 2024 Sorin M et al. *JAMA Oncology.*

eFigure 11. Pooled Risk Ratios Across Randomized Clinical Trials Comparing Neoadjuvant Chemoimmunotherapy With Neoadjuvant Chemotherapy by Number of Patients Who Received All Cycles of Neoadjuvant Treatment

eFigure 12. Pooled Risk Ratios Across Randomized Clinical Trials Comparing Neoadjuvant Chemoimmunotherapy With Neoadjuvant Chemotherapy by Number of Patients Who Received a Pneumonectomy

eFigure 13. Pooled Risk Ratios Across Randomized Clinical Trials Comparing Neoadjuvant Chemoimmunotherapy With Neoadjuvant Chemotherapy by Number of Patients Who Received a Bilobectomy

eFigure 14. Pooled Risk Ratios Across Randomized Clinical Trials Comparing Neoadjuvant Chemoimmunotherapy With Neoadjuvant Chemotherapy by Number of Patients Who Had Surgical Delay

eFigure 15. Pooled Risk Ratios Across Randomized Clinical Trials Comparing Neoadjuvant Chemoimmunotherapy With Neoadjuvant Chemotherapy by Number of Patients Who Had Open Surgery

eFigure 16. Pooled Risk Ratios Across Randomized Clinical Trials Comparing Neoadjuvant Chemoimmunotherapy With Neoadjuvant Chemotherapy by Number of Patients Who Had Grade 3-4 Treatment-Related Adverse Events

eFigure 17. Pooled Risk Ratios Across Randomized Clinical Trials Comparing Neoadjuvant Chemoimmunotherapy With Neoadjuvant Chemotherapy by Number of Patients Who Had Grade 5 Treatment-Related Adverse Events

eFigure 18. Pooled Risk Ratios Across Randomized Clinical Trials Comparing Neoadjuvant Chemoimmunotherapy With Neoadjuvant Chemotherapy by Number of Patients Who Had Treatment-Related Adverse Events

eFigure 19. Pooled Risk Ratios Across Randomized Clinical Trials Comparing Neoadjuvant Chemoimmunotherapy With Neoadjuvant Chemotherapy by Number of Patients Who Had Grade 3-4 Surgery-Related Adverse Events

eFigure 20. Pooled Risk Ratios Across Randomized Clinical Trials Comparing Neoadjuvant Chemoimmunotherapy With Neoadjuvant Chemotherapy by Number of Patients Who Had Grade 5 Surgery-Related Adverse Events

eFigure 21. Pooled Risk Ratios Across Randomized Clinical Trials Comparing Neoadjuvant Chemoimmunotherapy With Neoadjuvant Chemotherapy by Number of Patients Who Had Surgery-Related Adverse Events

eFigure 22. Pooled Risk Ratios Across Randomized Clinical Trials Comparing Neoadjuvant Chemoimmunotherapy With Neoadjuvant Chemotherapy by Number of Patients Who Did Not Receive Surgery

eFigure 23. Pooled Risk Ratios Across Randomized Clinical Trials Comparing Neoadjuvant Chemoimmunotherapy With Neoadjuvant Chemotherapy by Number of Patients Who Had Progression Precluding Surgery

© 2024 Sorin M et al. *JAMA Oncology.*

eFigure 24. Pooled Risk Ratios Across Randomized Clinical Trials Comparing Neoadjuvant Chemoimmunotherapy With Neoadjuvant Chemotherapy by Number of Patients Who Had Adverse Events Precluding Surgery

eFigure 25. Pooled Risk Ratios Across Randomized Clinical Trials Comparing Neoadjuvant Chemoimmunotherapy With Neoadjuvant Chemotherapy by Number of Patients With Refusal Precluding Surgery

eFigure 26. Pooled Risk Ratios Across Randomized Clinical Trials Comparing Neoadjuvant Chemoimmunotherapy With Neoadjuvant Chemotherapy by Number of Patients With Other Reasons Precluding Surgery

eFigure 27. Pooled Proportions Across All Studies Comparing Neoadjuvant Chemoimmunotherapy With Neoadjuvant Chemotherapy by Major Pathological Response

eFigure 28. Pooled Proportions Across All Studies Comparing Neoadjuvant Chemoimmunotherapy With Neoadjuvant Chemotherapy by Complete Pathological Response

eFigure 29. Pooled Proportions Across All Studies Comparing Neoadjuvant Chemoimmunotherapy With Neoadjuvant Chemotherapy by Complete Response per RECIST

eFigure 30. Pooled Proportions Across All Studies Comparing Neoadjuvant Chemoimmunotherapy With Neoadjuvant Chemotherapy by Partial Response per RECIST

eFigure 31. Pooled Proportions Across All Studies Comparing Neoadjuvant Chemoimmunotherapy With Neoadjuvant Chemotherapy by Stable Disease per RECIST

eFigure 32. Pooled Proportions Across All Studies Comparing Neoadjuvant Chemoimmunotherapy With Neoadjuvant Chemotherapy by Progressive Disease per RECIST.

eFigure 33. Pooled Proportions Across All Studies Comparing Neoadjuvant Chemoimmunotherapy With Neoadjuvant Chemotherapy by Surgical Resection

eFigure 34. Pooled Proportions Across All Studies Comparing Neoadjuvant Chemoimmunotherapy With Neoadjuvant Chemotherapy by the Number of Patients Who Had R0 Resection

eFigure 35. Pooled Proportions Across All Studies Comparing Neoadjuvant Chemoimmunotherapy With Neoadjuvant Chemotherapy by the Number of Patients Who Had a Lobectomy

eFigure 36. Pooled Proportions Across All Studies Comparing Neoadjuvant Chemoimmunotherapy With Neoadjuvant Chemotherapy by the Number of Patients Who Had a Pneumonectomy

eFigure 37. Pooled Proportions Across All Studies Comparing Neoadjuvant Chemoimmunotherapy With Neoadjuvant Chemotherapy by the Incidence of Death Events

© 2024 Sorin M et al. *JAMA Oncology.*

eTable 1. Search Strategy for Embase.

eTable 2. Search Strategy for MEDLINE.

eTable 3. Nonrandomized Study Characteristics

eTable 4. Joanna Briggs Bias Assessment

eTable 5. Surgical and Efficacy Outcomes for Randomized Clinical Trials

eTable 6. Definition of Event-Free Survival, Progression-Free Survival and Criteria Used for Pathological End Points

eTable 7. Reasons Precluding Surgery

This supplemental material has been provided by the authors to give readers additional information about their work.

© 2024 Sorin M et al. *JAMA Oncology.*

eMethods. Methodology

Study Protocol

The study was pre-registered with PROSPERO with the registration number CRD42023392998, and the full protocol has been made available.

Extracted Data

Extracted data were as follows: (I) study information, including the title, first author, year of publication, country of origin (or international), clinical trial phase, study design, inclusion and exclusion criteria for patients, definition of endpoints reported, neoadjuvant treatment regimens, and the intention-to-treat sample size; (II) baseline characteristics of the included patients, including sex, age, stage, lung cancer histology, ethnicity, smoking status, and tumor PD-L1 expression; (III) data on treatment-related outcomes, including the number of patients who achieved MPR, pCR, and clinical response measures (complete, partial, stable disease, and progressive disease). The total incidence of grade 3-4 and grade 5 TRAEs adverse events were also extracted. Relevant data related to OS, EFS, PFS, RFS, and DFS were extracted if available; (IV) surgical outcome measures were also extracted, including the incidence of grade 3-4, grade 5, and any grade SRAEs, the number of patients who received surgery, the type of operation performed, the incidence of R0, R1, R2 and Rx resection margins, and the total number of patients who received any subsequent adjuvant treatment.

Criteria and Definitions of Endpoints for RCTs

Among RCTs, major pathological response was defined as 10% or less viable tumor cells in the primary tumor and sampled lymph nodes while complete pathological response was defined as the absence of residual tumor in the primary tumor and sampled lymph nodes. Overall survival was defined as the time from randomization to death. Definitions of disease-free survival and progression-free survival can be found in Supplemental Table 5. Adverse events were graded using the Common Terminology Crieteria for Adevrse Events (version 4.0 for CheckMate 816, version 5.0 for NADIM II and version 4.03 for Keynote 671). For staging purposes, the AJCC was used (7th edition for CheckMate 816, 8th edition for NADIM II and Keynote 671).

© 2024 Sorin M et al. *JAMA Oncology.*

eFigure 1. PRISMA (Preferred Reporting Items for Systematic Reviews and Meta-Analyses) diagram.

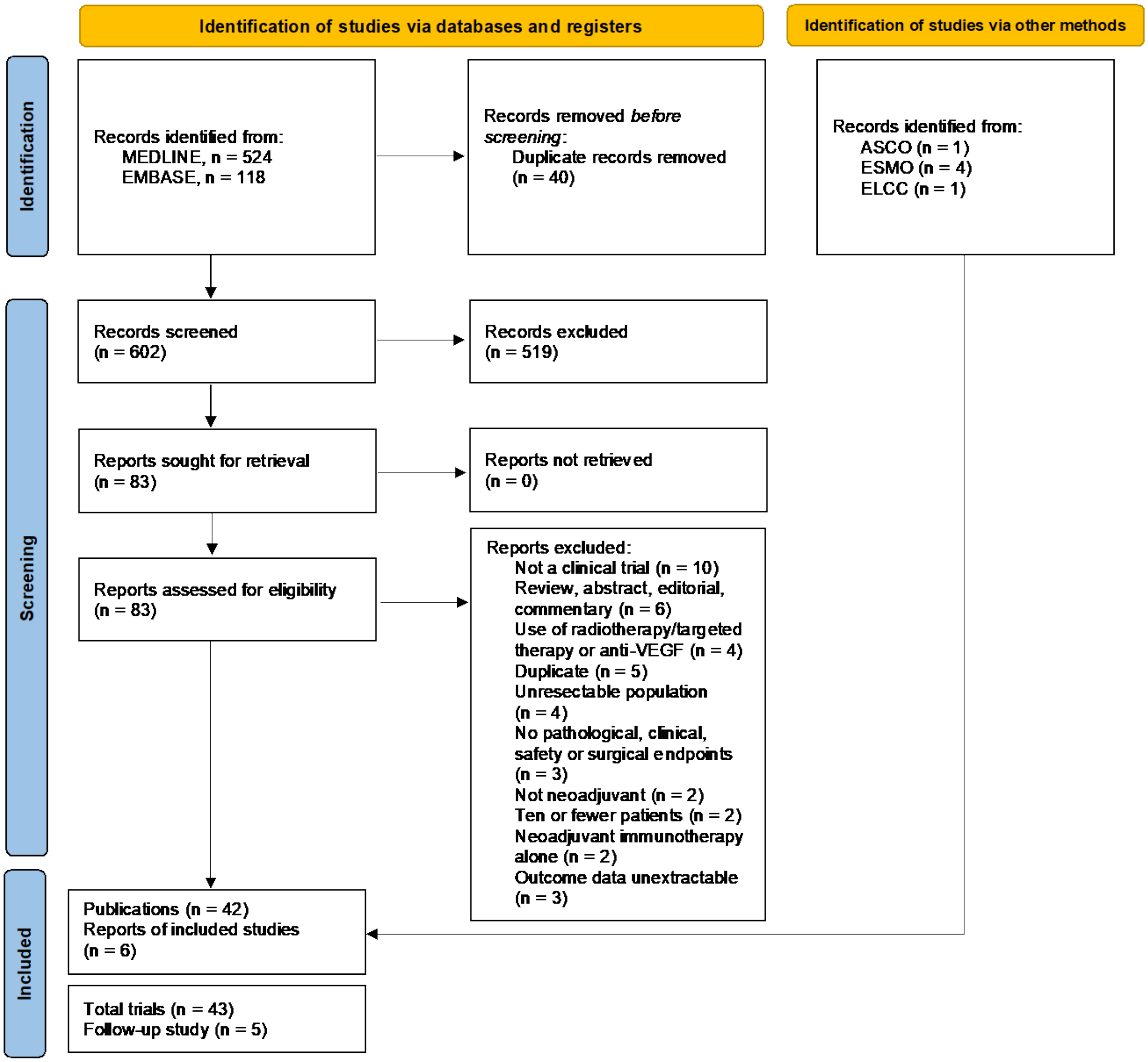

© 2024 Sorin M et al. *JAMA Oncology.*

eFigure 2. RCT risk of bias (ROB2) assessment.

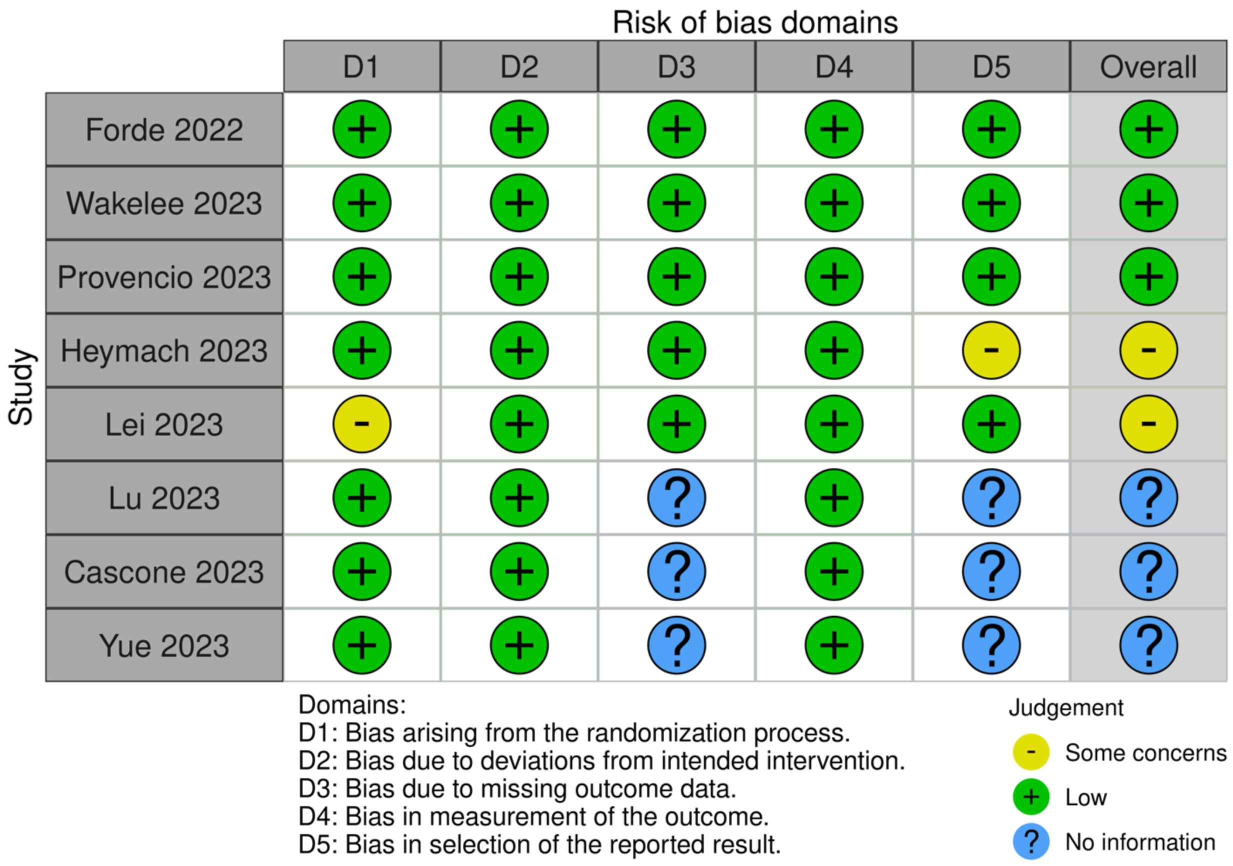

© 2024 Sorin M et al. *JAMA Oncology.*

eFigure 3. Pooled hazard ratios of EFS across RCTs comparing neoadjuvant chemoimmunotherapy to neoadjuvant chemotherapy by geographic region. $For Wakelee 2023, the geographic region was East Asia.

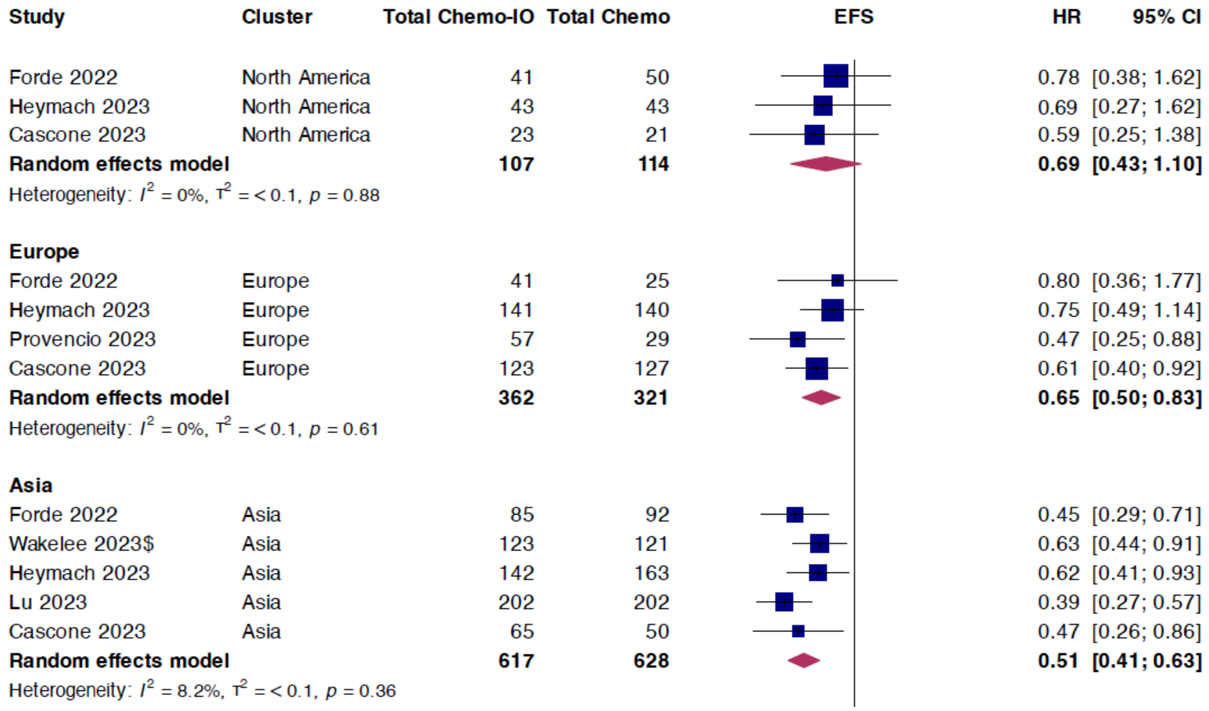

© 2024 Sorin M et al. *JAMA Oncology.*

eFigure 4. Pooled hazard ratios of EFS across RCTs comparing neoadjuvant chemoimmunotherapy to neoadjuvant chemotherapy by age.

Study	Cluster	Total Chemo-IO	Total Chemo	HR	95% CI
**<65yr**					
Forde 2022	<65yr	93	83	0.57	[0.35; 0.93]
Wakelee 2023	<65yr	221	214	0.51	[0.39; 0.67]
**Random effects model**		**314**	**297**	**0.52**	**[0.41; 0.66]**
Heterogeneity: $I^2 = 0\%$, $\tau^2 = < 0.1$, $p = 0.70$					
**≥65yr**					
Forde 2022	≥65yr	86	96	0.70	[0.45; 1.08]
Wakelee 2023	≥65yr	176	186	0.70	[0.52; 0.92]
**Random effects model**		**262**	**282**	**0.70**	**[0.55; 0.88]**
Heterogeneity: $I^2 = 0\%$, $\tau^2 = < 0.1$, $p = 0.98$					

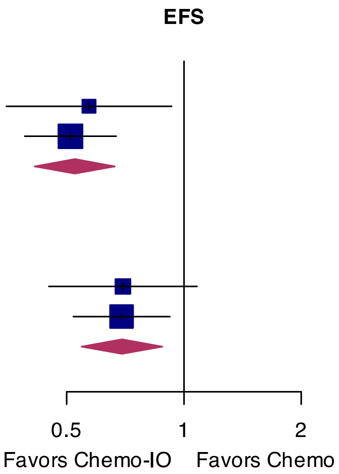

© 2024 Sorin M et al. *JAMA Oncology.*

eFigure 5. Pooled hazard ratios of EFS across RCTs comparing neoadjuvant chemoimmunotherapy to neoadjuvant chemotherapy by sex.

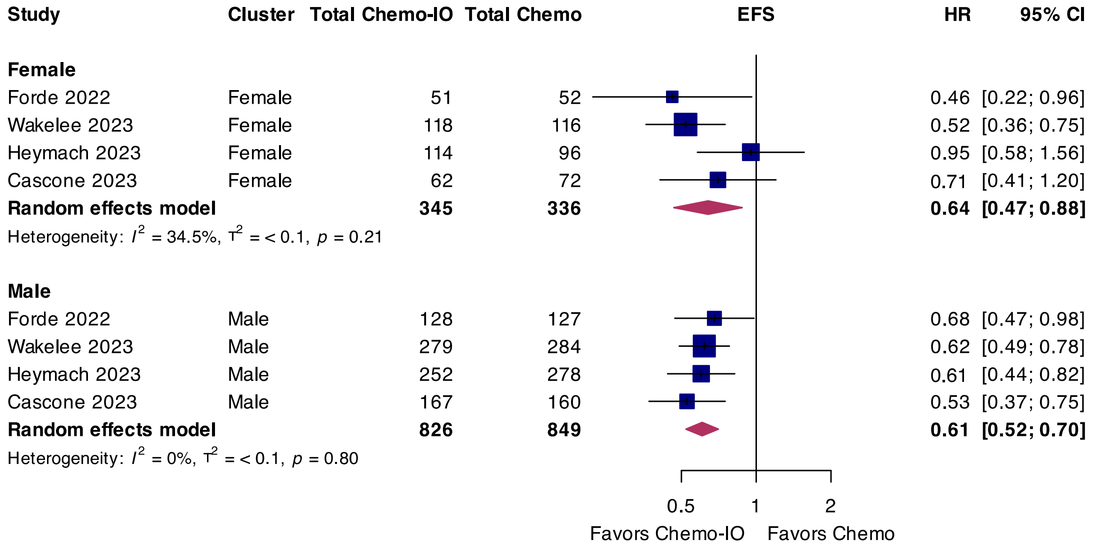

eFigure 6. Pooled hazard ratios of EFS across RCTs comparing neoadjuvant chemoimmunotherapy to neoadjuvant chemotherapy by histology.

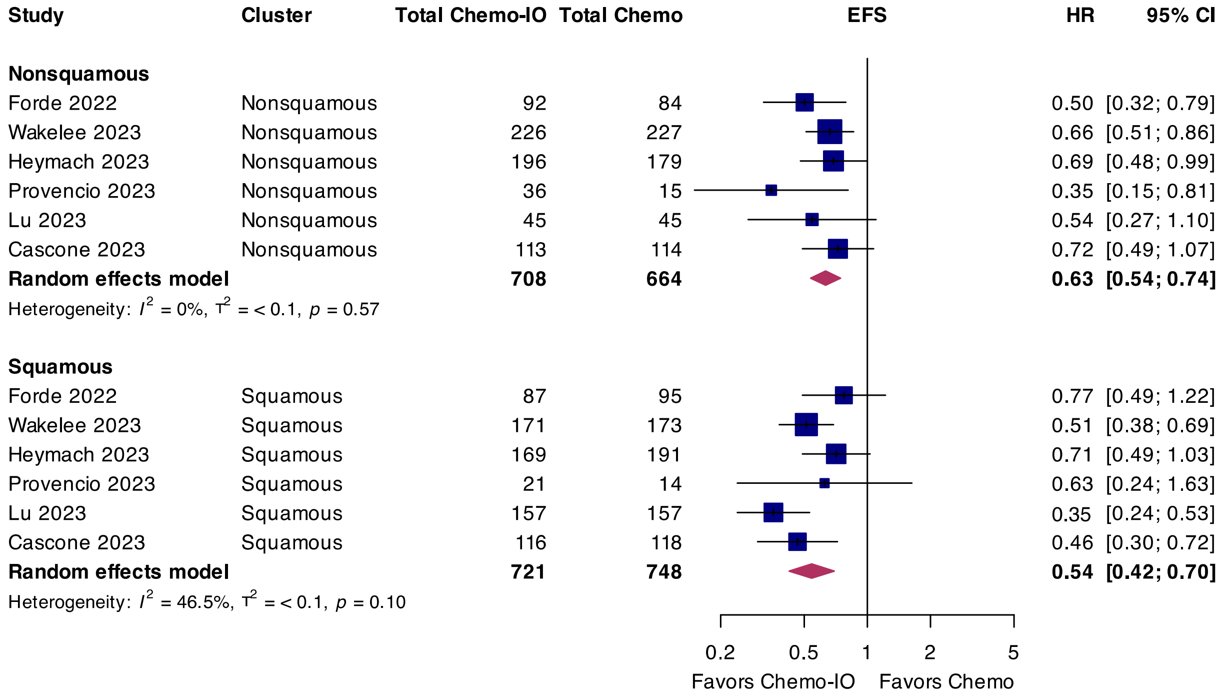

© 2024 Sorin M et al. *JAMA Oncology.*

eFigure 7. Pooled hazard ratios of EFS across RCTs comparing neoadjuvant chemoimmunotherapy to neoadjuvant chemotherapy by chemotherapy type.

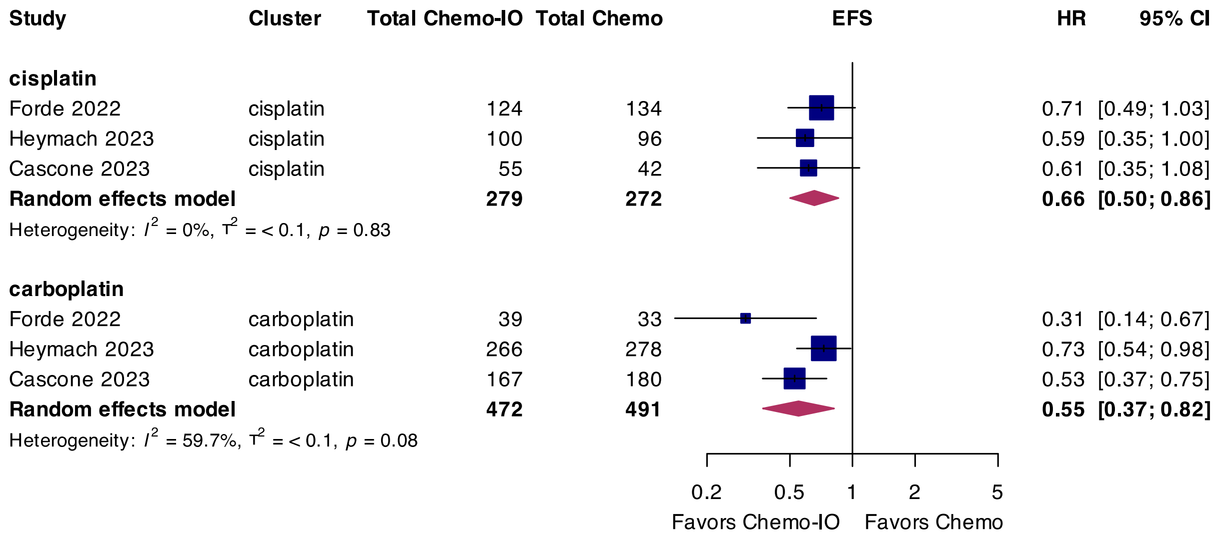

© 2024 Sorin M et al. *JAMA Oncology.*

eFigure 8. Pooled risk ratios of MPR across RCTs comparing neoadjuvant chemoimmunotherapy to neoadjuvant chemotherapy by sex, geographic region, histology, stage, PD-L1 status and chemotherapy type.

Study	Subgroup	Chemo-IO Events	Chemo-IO Total	Chemo Events	Chemo Total	RR [95%CI]
**Female**						
Heymach 2023	Female	28	114	10	96	2.36 [1.21; 4.60]
Forde 2022	Female	19	51	6	52	3.23 [1.40; 7.42]
Lei 2023	Female	6	9	1	5	3.33 [0.54; 20.43]
Yue 2023	Female	7	21	3	22	2.44 [0.73; 8.22]
**Total (95% CI)**		**60**	**195**	**20**	**175**	**2.67 [1.68; 4.25]**
Heterogeneity: Tau^2 = < 0.1; Chi^2 = 0.4, df = 3 (P = 0.94); I^2 = 0%						
**Male**						
Heymach 2023	Male	94	252	36	278	2.88 [2.04; 4.06]
Forde 2022	Male	47	128	10	127	4.66 [2.47; 8.82]
Lei 2023	Male	22	34	6	40	4.31 [1.98; 9.39]
Yue 2023	Male	120	205	31	205	3.87 [2.74; 5.46]
**Total (95% CI)**		**283**	**619**	**83**	**650**	**3.56 [2.82; 4.50]**
Heterogeneity: Tau^2 = < 0.1; Chi^2 = 2.6, df = 3 (P = 0.46); I^2 = 0%						
**North America**						
Heymach 2023	North America	9	43	8	43	1.12 [0.48; 2.64]
Forde 2022	North America	13	41	5	50	3.17 [1.23; 8.16]
**Total (95% CI)**		**22**	**84**	**13**	**93**	**1.85 [0.67; 5.10]**
Heterogeneity: Tau^2 = 0.3; Chi^2 = 2.5, df = 1 (P = 0.11); I^2 = 60.7%						
**Europe**						
Heymach 2023	Europe	51	141	17	140	2.98 [1.81; 4.89]
Forde 2022	Europe	18	41	2	25	5.49 [1.39; 21.67]
**Total (95% CI)**		**69**	**182**	**19**	**165**	**3.20 [2.00; 5.10]**
Heterogeneity: Tau^2 = < 0.1; Chi^2 = 0.7, df = 1 (P = 0.41); I^2 = 0%						
**Asia**						
Heymach 2023	Asia	53	142	19	163	3.20 [1.99; 5.14]
Forde 2022	Asia	33	85	8	92	4.46 [2.19; 9.12]
**Total (95% CI)**		**86**	**227**	**27**	**255**	**3.54 [2.39; 5.26]**
Heterogeneity: Tau^2 = < 0.1; Chi^2 = 0.6, df = 1 (P = 0.45); I^2 = 0%						
**Nonsquamous**						
Heymach 2023	Nonsquamous	55	196	11	179	4.57 [2.47; 8.44]
Forde 2022	Nonsquamous	36	92	3	84	10.96 [3.50; 34.26]
Lei 2023	Nonsquamous	7	15	1	11	5.13 [0.73; 35.92]
Yue 2023	Nonsquamous	18	45	5	50	4.00 [1.62; 9.89]
**Total (95% CI)**		**116**	**348**	**20**	**324**	**5.10 [3.25; 8.01]**
Heterogeneity: Tau^2 = < 0.1; Chi^2 = 2.1, df = 3 (P = 0.55); I^2 = 0%						
**Squamous**						
Heymach 2023	Squamous	66	169	35	191	2.13 [1.50; 3.04]
Forde 2022	Squamous	30	87	13	95	2.52 [1.41; 4.51]
Lei 2023	Squamous	20	27	6	32	3.95 [1.86; 8.41]
Yue 2023	Squamous	107	179	29	175	3.61 [2.53; 5.14]
**Total (95% CI)**		**223**	**462**	**83**	**493**	**2.85 [2.09; 3.90]**
Heterogeneity: Tau^2 = < 0.1; Chi^2 = 5.2, df = 3 (P = 0.16); I^2 = 42.1%						
**Stage II**						
Heymach 2023	Stage II	36	104	12	110	3.17 [1.75; 5.76]
Forde 2022	Stage II	20	65	5	62	3.82 [1.53; 9.54]
Yue 2023	Stage II	49	93	17	93	2.88 [1.80; 4.62]
**Total (95% CI)**		**105**	**262**	**34**	**265**	**3.09 [2.20; 4.36]**
Heterogeneity: Tau^2 = < 0.1; Chi^2 = 0.3, df = 2 (P = 0.86); I^2 = 0%						
**Stage III**						
Heymach 2023a	Stage III	62	173	21	165	2.82 [1.80; 4.40]
Heymach 2023b	Stage III	24	88	13	98	2.06 [1.12; 3.79]
Forde 2022	Stage III	46	113	11	115	4.26 [2.33; 7.79]
Lei 2023a	Stage III	20	30	5	36	4.80 [2.05; 11.25]
Lei 2023b	Stage III	8	13	2	9	2.77 [0.76; 10.12]
Yue 2023	Stage III	77	132	17	132	4.53 [2.84; 7.22]
**Total (95% CI)**		**237**	**549**	**69**	**555**	**3.40 [2.54; 4.55]**
Heterogeneity: Tau^2 = < 0.1; Chi^2 = 6, df = 5 (P = 0.31); I^2 = 16.7%						
**PD-L1 <1%**						
Heymach 2023	PD-L1 <1%	23	122	14	125	1.68 [0.91; 3.12]
Forde 2022	PD-L1 <1%	23	78	11	77	2.06 [1.08; 3.94]
Lei 2023	PD-L1 <1%	6	7	3	8	2.29 [0.89; 5.88]
Yue 2023	PD-L1 <1%	43	89	14	84	2.90 [1.72; 4.90]
**Total (95% CI)**		**95**	**296**	**42**	**294**	**2.24 [1.63; 3.09]**
Heterogeneity: Tau^2 = < 0.1; Chi^2 = 1.8, df = 3 (P = 0.61); I^2 = 0%						
**PD-L1 1-49%**						
Heymach 2023	PD-L1 1-49%	44	135	19	142	2.44 [1.50; 3.95]
Forde 2022	PD-L1 1-49%	21	51	2	47	9.68 [2.40; 39.05]
**Total (95% CI)**		**65**	**186**	**21**	**189**	**4.13 [1.11; 15.37]**
Heterogeneity: Tau^2 = 0.7; Chi^2 = 3.4, df = 1 (P = 0.07); I^2 = 70.2%						
**PD-L1 ≥50%**						
Heymach 2023	PD-L1 ≥50%	55	109	13	107	4.15 [2.41; 7.14]
Forde 2022	PD-L1 ≥50%	19	38	3	42	7.00 [2.25; 21.80]
**Total (95% CI)**		**74**	**147**	**16**	**149**	**4.58 [2.80; 7.47]**
Heterogeneity: Tau^2 = < 0.1; Chi^2 = 0.7, df = 1 (P = 0.42); I^2 = 0%						
**cisplatin**						
Heymach 2023	cisplatin	32	100	7	96	4.39 [2.04; 9.46]
Forde 2022	cisplatin	45	124	12	134	4.05 [2.25; 7.30]
**Total (95% CI)**		**77**	**224**	**19**	**230**	**4.17 [2.62; 6.66]**
Heterogeneity: Tau^2 = < 0.1; Chi^2 = 0, df = 1 (P = 0.87); I^2 = 0%						
**carboplatin**						
Heymach 2023	carboplatin	90	266	39	278	2.41 [1.72; 3.38]
Forde 2022	carboplatin	16	39	3	33	4.51 [1.44; 14.15]
**Total (95% CI)**		**106**	**305**	**42**	**311**	**2.58 [1.77; 3.75]**
Heterogeneity: Tau^2 = < 0.1; Chi^2 = 1.1, df = 1 (P = 0.30); I^2 = 5.9%						

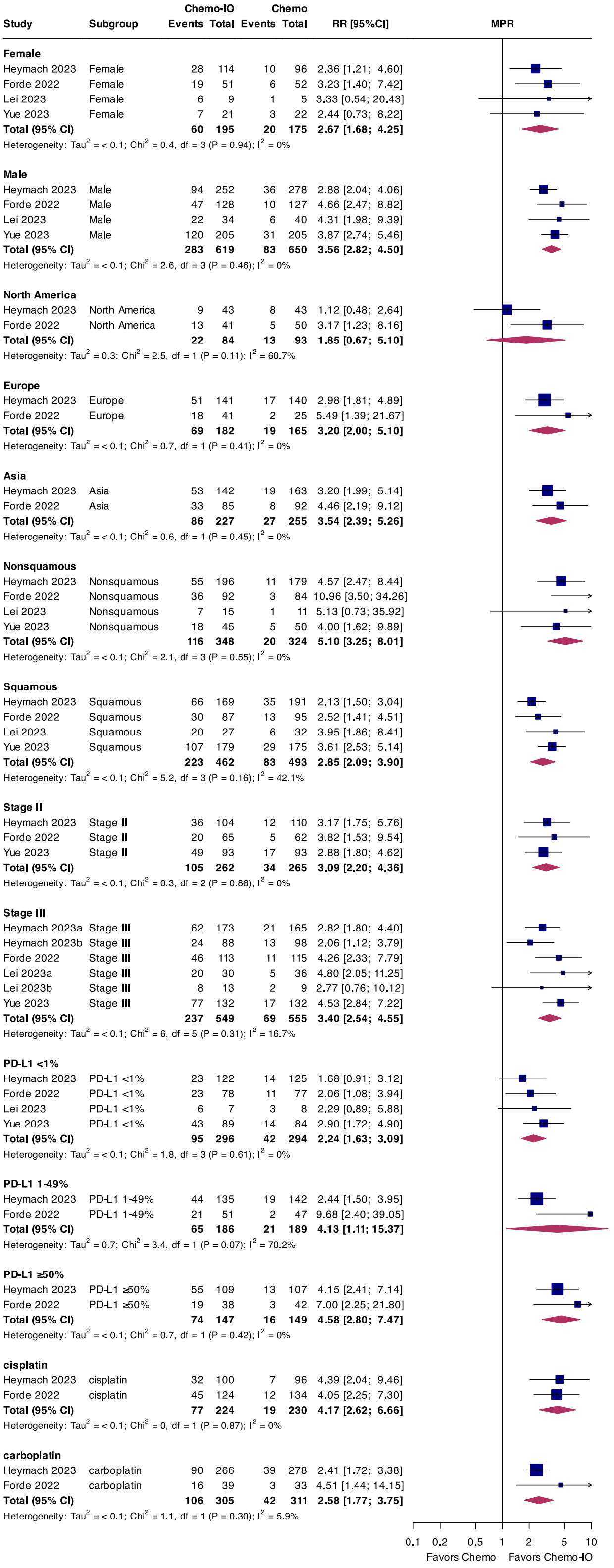

© 2024 Sorin M et al. *JAMA Oncology.*

eFigure 9. Pooled risk ratios of pCR across RCTs comparing neoadjuvant chemoimmunotherapy to neoadjuvant chemotherapy by sex, geographic region, histology, stage, PD-L1 status and chemotherapy type.

Study	Subgroup	Chemo-IO Events	Chemo-IO Total	Chemo Events	Chemo Total	RR [95%CI]
**Female**						
Heymach 2023	Female	14	114	3	96	3.93 [1.16; 13.27]
Provencio 2023	Female	10	21	2	13	3.10 [0.80; 11.96]
Forde 2022	Female	14	51	1	52	14.27 [1.95; 104.60]
Cascone 2023	Female	13	62	3	72	5.03 [1.50; 16.85]
Lei 2023	Female	2	9	0	5	2.89 [0.17; 49.99]
Yue 2023	Female	4	21	2	22	2.10 [0.43; 10.26]
**Total (95% CI)**		**57**	**278**	**11**	**260**	**4.04 [2.20; 7.45]**
Heterogeneity: Tau^2 = < 0.1; Chi^2 = 2.5, df = 5 (P = 0.77); I^2 = 0%						
**Male**						
Heymach 2023	Male	49	252	13	278	4.16 [2.31; 7.48]
Provencio 2023	Male	11	36	0	16	10.40 [0.65; 166.11]
Forde 2022	Male	29	128	3	127	9.59 [3.00; 30.69]
Cascone 2023	Male	45	167	8	160	5.39 [2.62; 11.07]
Lei 2023	Male	12	34	4	40	3.53 [1.25; 9.94]
Yue 2023	Male	88	205	11	205	8.00 [4.41; 14.52]
**Total (95% CI)**		**234**	**822**	**39**	**826**	**5.67 [4.03; 7.98]**
Heterogeneity: Tau^2 = < 0.1; Chi^2 = 4.1, df = 5 (P = 0.53); I^2 = 0%						
**North America**						
Heymach 2023	North America	6	43	1	43	6.00 [0.75; 47.76]
Forde 2022	North America	9	41	1	50	10.98 [1.45; 83.09]
Cascone 2023	North America	3	23	0	21	6.40 [0.35; 116.96]
**Total (95% CI)**		**18**	**107**	**2**	**114**	**7.79 [2.13; 28.47]**
Heterogeneity: Tau^2 = < 0.1; Chi^2 = 0.2, df = 2 (P = 0.91); I^2 = 0%						
**Europe**						
Heymach 2023	Europe	28	141	7	140	3.97 [1.79; 8.79]
Forde 2022	Europe	10	41	0	25	12.90 [0.79; 210.89]
Cascone 2023	Europe	27	123	7	127	3.98 [1.80; 8.81]
**Total (95% CI)**		**65**	**305**	**14**	**292**	**4.16 [2.40; 7.22]**
Heterogeneity: Tau^2 = < 0.1; Chi^2 = 0.7, df = 2 (P = 0.72); I^2 = 0%						
**Asia**						
Heymach 2023	Asia	26	142	7	163	4.26 [1.91; 9.52]
Forde 2022	Asia	24	85	3	92	8.66 [2.71; 27.72]
Cascone 2023	Asia	24	65	1	50	18.46 [2.58; 131.87]
**Total (95% CI)**		**74**	**292**	**11**	**305**	**6.43 [3.11; 13.29]**
Heterogeneity: Tau^2 = < 0.1; Chi^2 = 2.3, df = 2 (P = 0.31); I^2 = 14.2%						
**Nonsquamous**						
Heymach 2023	Nonsquamous	26	196	6	179	3.96 [1.67; 9.39]
Provencio 2023	Nonsquamous	14	36	0	15	12.32 [0.78; 193.77]
Forde 2022	Nonsquamous	21	92	0	84	39.28 [2.42; 638.45]
Cascone 2023	Nonsquamous	25	113	4	114	6.31 [2.27; 17.54]
Lei 2023	Nonsquamous	3	15	0	11	5.19 [0.30; 90.99]
Yue 2023	Nonsquamous	16	45	2	50	8.89 [2.16; 36.54]
**Total (95% CI)**		**105**	**497**	**12**	**453**	**6.01 [3.43; 10.53]**
Heterogeneity: Tau^2 = < 0.1; Chi^2 = 3.2, df = 5 (P = 0.67); I^2 = 0%						
**Squamous**						
Heymach 2023	Squamous	36	169	10	191	4.07 [2.08; 7.95]
Provencio 2023	Squamous	7	21	2	14	2.33 [0.56; 9.64]
Forde 2022	Squamous	22	87	4	95	6.01 [2.16; 16.74]
Cascone 2023	Squamous	33	116	7	118	4.80 [2.21; 10.40]
Lei 2023	Squamous	10	27	4	32	2.96 [1.05; 8.39]
Yue 2023	Squamous	74	179	11	175	6.58 [3.62; 11.96]
**Total (95% CI)**		**182**	**599**	**38**	**625**	**4.76 [3.41; 6.63]**
Heterogeneity: Tau^2 = < 0.1; Chi^2 = 3.3, df = 5 (P = 0.65); I^2 = 0%						
**Stage II**						
Heymach 2023	Stage II	22	104	5	110	4.65 [1.83; 11.83]
Forde 2022	Stage II	17	65	3	62	5.41 [1.67; 17.54]
Cascone 2023	Stage II	24	81	3	81	8.00 [2.51; 25.52]
Yue 2023	Stage II	36	93	5	93	7.20 [2.96; 17.54]
**Total (95% CI)**		**99**	**343**	**16**	**346**	**6.12 [3.68; 10.17]**
Heterogeneity: Tau^2 = < 0.1; Chi^2 = 0.7, df = 3 (P = 0.87); I^2 = 0%						
**Stage III**						
Heymach 2023a	Stage III	32	173	8	165	3.82 [1.81; 8.03]
Heymach 2023b	Stage III	9	88	3	98	3.34 [0.93; 11.95]
Forde 2022	Stage III	26	113	1	115	26.46 [3.65; 191.71]
Cascone 2023	Stage III	34	148	8	151	4.34 [2.08; 9.05]
Lei 2023a	Stage III	9	30	3	36	3.60 [1.07; 12.11]
Lei 2023b	Stage III	5	13	1	9	3.46 [0.48; 24.86]
Yue 2023	Stage III	55	132	8	132	6.88 [3.41; 13.86]
**Total (95% CI)**		**170**	**697**	**32**	**706**	**4.84 [3.36; 6.97]**
Heterogeneity: Tau^2 = < 0.1; Chi^2 = 4.9, df = 6 (P = 0.55); I^2 = 0%						
**PD-L1 <1%**						
Heymach 2023	PD-L1 <1%	11	122	4	125	2.82 [0.92; 8.61]
Forde 2022	PD-L1 <1%	13	78	2	77	6.42 [1.50; 27.49]
Cascone 2023	PD-L1 <1%	12	93	4	93	3.00 [1.00; 8.96]
Lei 2023	PD-L1 <1%	4	7	1	8	4.57 [0.66; 31.89]
Yue 2023	PD-L1 <1%	33	89	7	84	4.45 [2.08; 9.51]
**Total (95% CI)**		**73**	**389**	**18**	**387**	**3.93 [2.40; 6.43]**
Heterogeneity: Tau^2 = < 0.1; Chi^2 = 1.1, df = 4 (P = 0.89); I^2 = 0%						
**PD-L1 1-49%**						
Heymach 2023	PD-L1 1-49%	22	135	7	142	3.31 [1.46; 7.49]
Forde 2022	PD-L1 1-49%	12	51	0	47	23.06 [1.40; 378.83]
Cascone 2023	PD-L1 1-49%	22	83	3	76	6.71 [2.09; 21.54]
**Total (95% CI)**		**56**	**269**	**10**	**265**	**4.76 [2.31; 9.82]**
Heterogeneity: Tau^2 = < 0.1; Chi^2 = 2.3, df = 2 (P = 0.32); I^2 = 13.3%						
**PD-L1 ≥50%**						
Heymach 2023	PD-L1 ≥50%	30	109	5	107	5.89 [2.37; 14.61]
Forde 2022	PD-L1 ≥50%	17	38	2	42	9.39 [2.32; 38.02]
Cascone 2023	PD-L1 ≥50%	23	45	3	52	8.86 [2.85; 27.56]
**Total (95% CI)**		**70**	**192**	**10**	**201**	**7.36 [3.91; 13.85]**
Heterogeneity: Tau^2 = < 0.1; Chi^2 = 0.5, df = 2 (P = 0.80); I^2 = 0%						
**cisplatin**						
Heymach 2023	cisplatin	12	100	2	96	5.76 [1.32; 25.06]
Forde 2022	cisplatin	27	124	3	134	9.73 [3.03; 31.26]
Cascone 2023	cisplatin	16	55	2	42	6.11 [1.49; 25.12]
**Total (95% CI)**		**55**	**279**	**7**	**272**	**7.35 [3.41; 15.84]**
Heterogeneity: Tau^2 = < 0.1; Chi^2 = 0.4, df = 2 (P = 0.82); I^2 = 0%						
**carboplatin**						
Heymach 2023	carboplatin	51	266	14	278	3.81 [2.16; 6.71]
Forde 2022	carboplatin	12	39	0	33	21.20 [1.30; 344.84]
Cascone 2023	carboplatin	41	167	9	180	4.91 [2.46; 9.79]
**Total (95% CI)**		**104**	**472**	**23**	**491**	**4.39 [2.84; 6.76]**
Heterogeneity: Tau^2 = < 0.1; Chi^2 = 1.6, df = 2 (P = 0.46); I^2 = 0%						

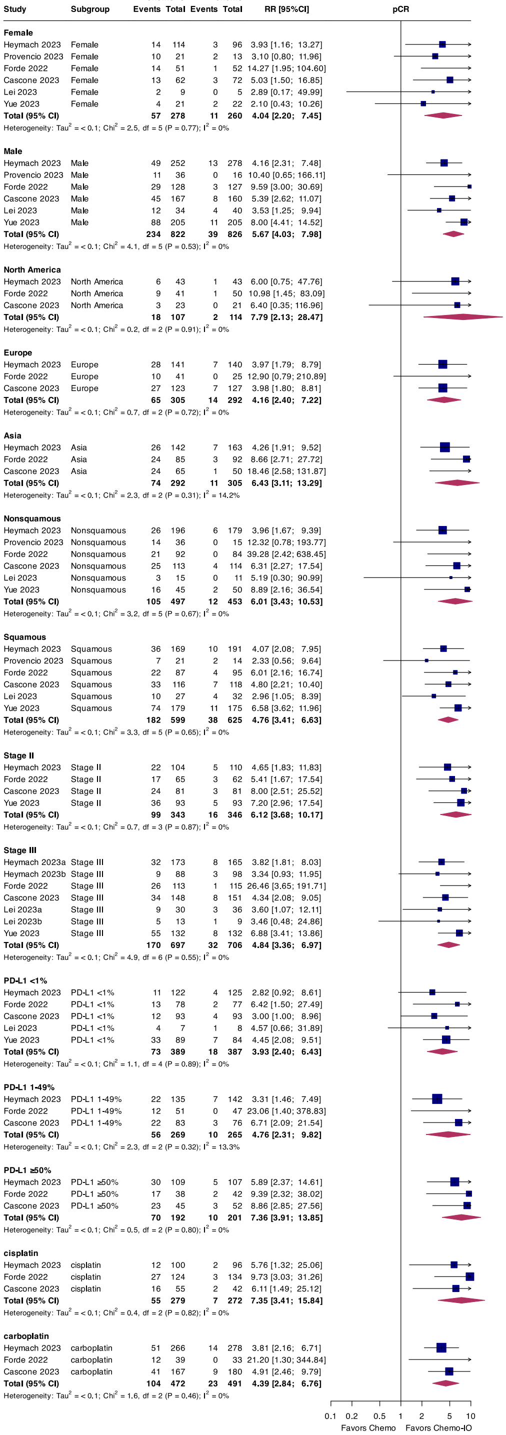

© 2024 Sorin M et al. *JAMA Oncology.*

eFigure 10. Pooled risk ratios across RCTs comparing neoadjuvant chemoimmunotherapy to neoadjuvant chemotherapy by the number of patients who received a lobectomy.

	Chemo−IO		Chemo			
**Study**	**Events**	**Total**	**Events**	**Total**	**Weight**	**RR [95% CI]**
Forde 2022	115	149	82	135	9.7%	1.27 [1.08; 1.49]
Wakelee 2023	256	325	238	317	21.7%	1.05 [0.96; 1.14]
Lu 2023	134	166	123	148	17.5%	0.97 [0.88; 1.08]
Heymach 2023	238	284	221	287	22.6%	1.09 [1.00; 1.18]
Provencio 2023	41	53	17	20	5.2%	0.91 [0.72; 1.15]
Cascone 2023	142	178	128	178	15.0%	1.11 [0.99; 1.25]
Lei 2023	36	40	34	42	8.2%	1.11 [0.93; 1.33]
**Total (95% CI)**	**962**	**1195**	**843**	**1127**	**100.0%**	**1.07 [1.01; 1.13]**

Heterogeneity: $Tau^2 < 0.1$; $Chi^2 = 10.4$, df = 6 (P = 0.11); $I^2 = 42.4\%$

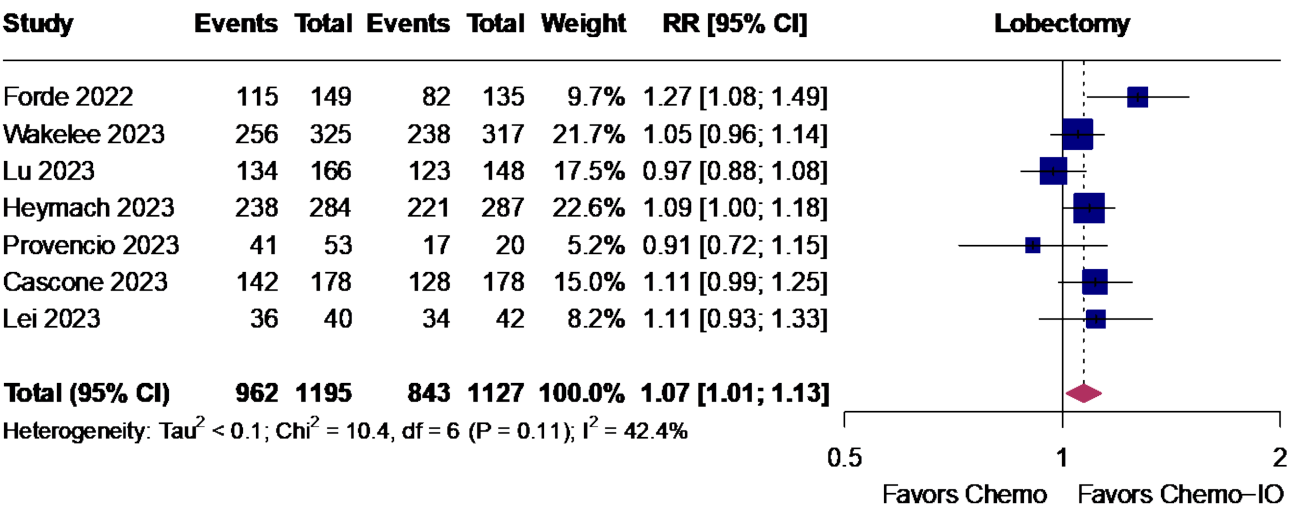

eFigure 11. Pooled risk ratios across RCTs comparing neoadjuvant chemoimmunotherapy to neoadjuvant chemotherapy by the number of patients who received all cycles of neoadjuvant treatment.

	Chemo−IO		Chemo			
**Study**	**Events**	**Total**	**Events**	**Total**	**Weight**	**RR [95% CI]**
Forde 2022	165	179	149	179	11.6%	1.11 [1.02; 1.20]
Wakelee 2023	295	397	297	400	11.0%	1.00 [0.92; 1.09]
Heymach 2023	318	366	331	374	17.4%	0.98 [0.93; 1.04]
Provencio 2023	53	57	28	29	8.4%	0.96 [0.87; 1.06]
Lei 2023	42	47	45	47	6.6%	0.93 [0.83; 1.05]
Lu 2023	176	202	185	202	13.9%	0.95 [0.89; 1.02]
Yue 2023	211	226	210	227	18.3%	1.01 [0.96; 1.06]
Cascone 2023	194	229	205	232	12.8%	0.96 [0.89; 1.03]
**Total (95% CI)**	**1454**	**1703**	**1450**	**1690**	**100.0%**	**0.99 [0.96; 1.02]**

Heterogeneity: $Tau^2 < 0.1$; $Chi^2 = 11.9$, df = 7 (P = 0.10); $I^2 = 41.4\%$

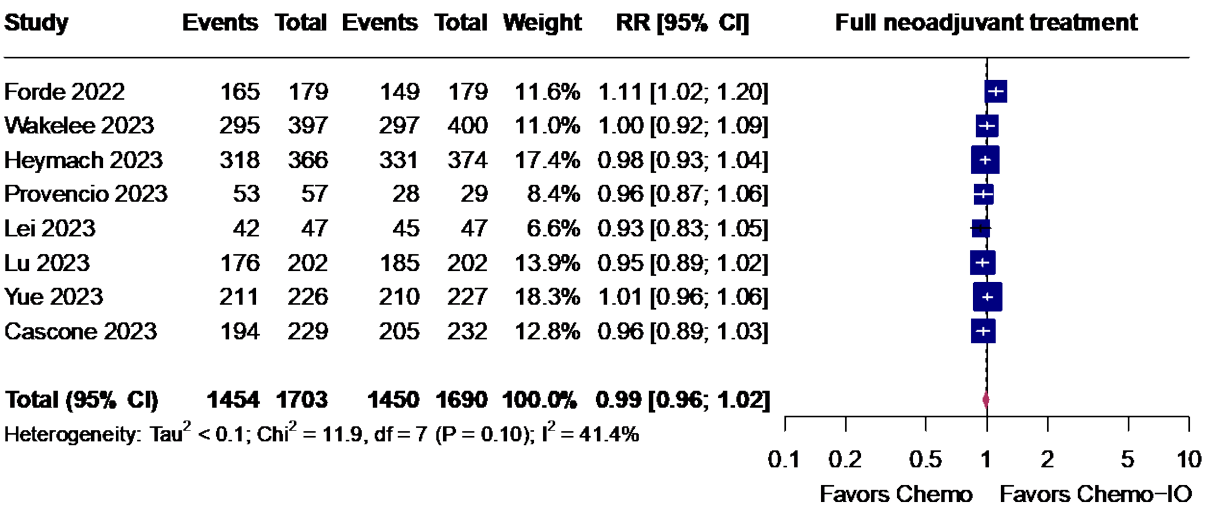

© 2024 Sorin M et al. *JAMA Oncology.*

eFigure 12. Pooled risk ratios across RCTs comparing neoadjuvant chemoimmunotherapy to neoadjuvant chemotherapy by the number of patients who received a pneumonectomy.

Study	Chemo–IO Events	Chemo–IO Total	Chemo Events	Chemo Total	Weight	RR [95% CI]
Forde 2022	25	149	34	135	23.0%	0.67 [0.42; 1.06]
Wakelee 2023	37	325	39	317	27.4%	0.93 [0.61; 1.41]
Lu 2023	15	166	14	148	10.2%	0.96 [0.48; 1.91]
Heymach 2023	27	284	29	287	19.7%	0.94 [0.57; 1.55]
Provencio 2023	6	53	2	20	2.1%	1.13 [0.25; 5.15]
Cascone 2023	16	178	24	178	13.7%	0.67 [0.37; 1.21]
Lei 2023	4	40	8	42	3.9%	0.53 [0.17; 1.61]
**Total (95% CI)**	**130**	**1195**	**150**	**1127**	**100.0%**	**0.81 [0.65; 1.01]**

Heterogeneity: $Tau^2 = 0$; $Chi^2 = 2.8$, df = 6 (P = 0.83); $I^2 = 0.0\%$

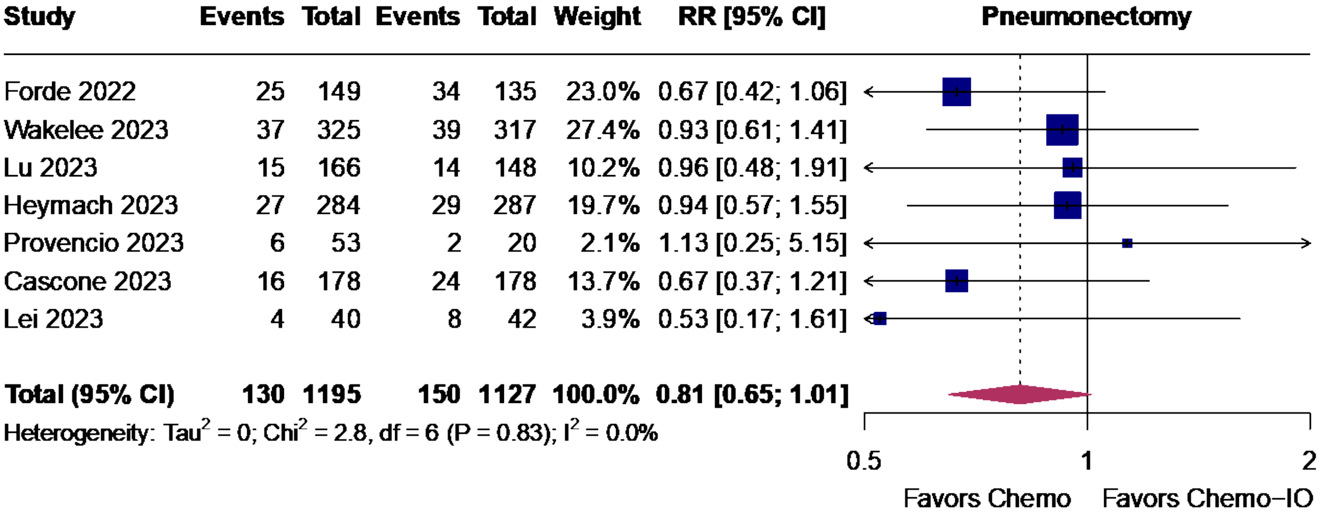

eFigure 13. Pooled risk ratios across RCTs comparing neoadjuvant chemoimmunotherapy to neoadjuvant chemotherapy by the number of patients who received a bilobectomy.

Study	Chemo–IO Events	Chemo–IO Total	Chemo Events	Chemo Total	Weight	RR [95% CI]
Forde 2022	3	149	4	135	7.0%	0.68 [0.15; 2.98]
Wakelee 2023	26	325	26	317	56.5%	0.98 [0.58; 1.64]
Heymach 2023	13	284	20	287	33.3%	0.66 [0.33; 1.29]
Provencio 2023	3	53	1	20	3.2%	1.13 [0.12; 10.26]
Lei 2023	0	40	0	42	0.0%	
**Total (95% CI)**	**45**	**851**	**51**	**801**	**100.0%**	**0.84 [0.57; 1.24]**

Heterogeneity: $Tau^2 = 0$; $Chi^2 = 1.0$, df = 3 (P = 0.81); $I^2 = 0.0\%$

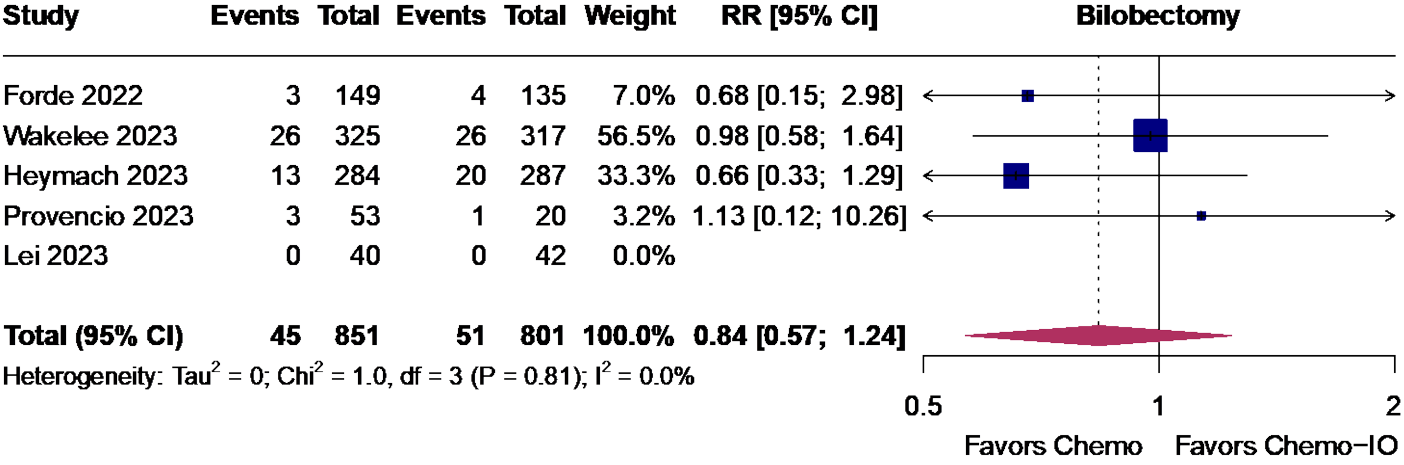

© 2024 Sorin M et al. *JAMA Oncology.*

eFigure 14. Pooled risk ratios across RCTs comparing neoadjuvant chemoimmunotherapy to neoadjuvant chemotherapy by the number of patients who had surgical delay.

	Chemo−IO		Chemo			
**Study**	**Events**	**Total**	**Events**	**Total**	**Weight**	**RR [95% CI]**
Forde 2022	31	149	24	135	33.6%	1.17 [0.72; 1.89]
Heymach 2023	58	284	67	287	64.3%	0.87 [0.64; 1.19]
Provencio 2023	0	53	0	20	0.0%	
Lei 2023	5	40	1	42	2.1%	5.25 [0.64; 43.00]
**Total (95% CI)**	**94**	**526**	**92**	**484**	**100.0%**	**1.00 [0.74; 1.36]**

Heterogeneity: $Tau^2 < 0.1$; $Chi^2 = 3.5$, df = 2 (P = 0.17); $I^2 = 42.6\%$

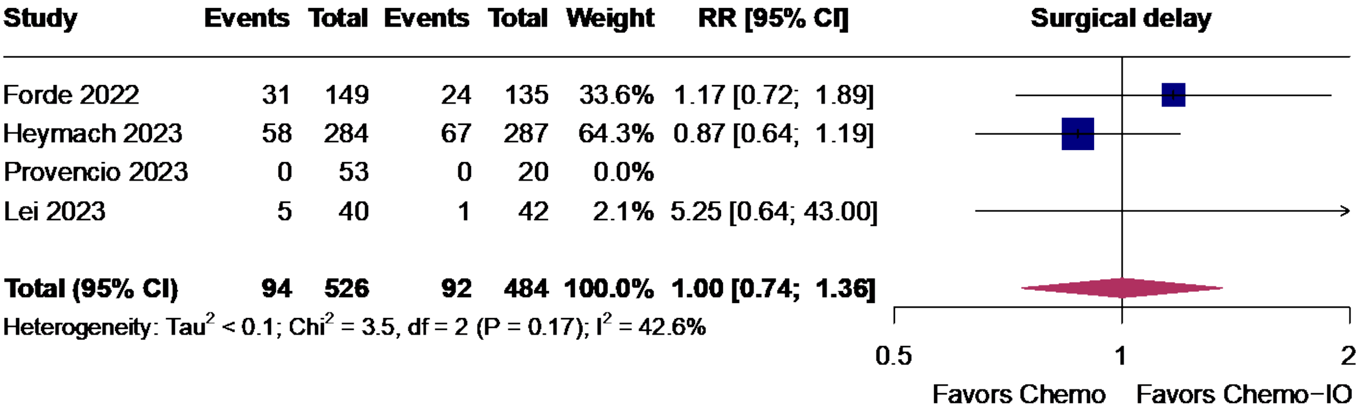

eFigure 15. Pooled risk ratios across RCTs comparing neoadjuvant chemoimmunotherapy to neoadjuvant chemotherapy by the number of patients who had open surgery.

	Chemo−IO		Chemo			
**Study**	**Events**	**Total**	**Events**	**Total**	**Weight**	**RR [95% CI]**
Forde 2022	88	149	85	135	40.0%	0.94 [0.78; 1.13]
Heymach 2023	145	284	153	287	56.0%	0.96 [0.82; 1.12]
Lei 2023	15	40	14	42	4.0%	1.12 [0.63; 2.02]
**Total (95% CI)**	**248**	**473**	**252**	**464**	**100.0%**	**0.96 [0.85; 1.08]**

Heterogeneity: $Tau^2 = 0$; $Chi^2 = 0.3$, df = 2 (P = 0.84); $I^2 = 0.0\%$

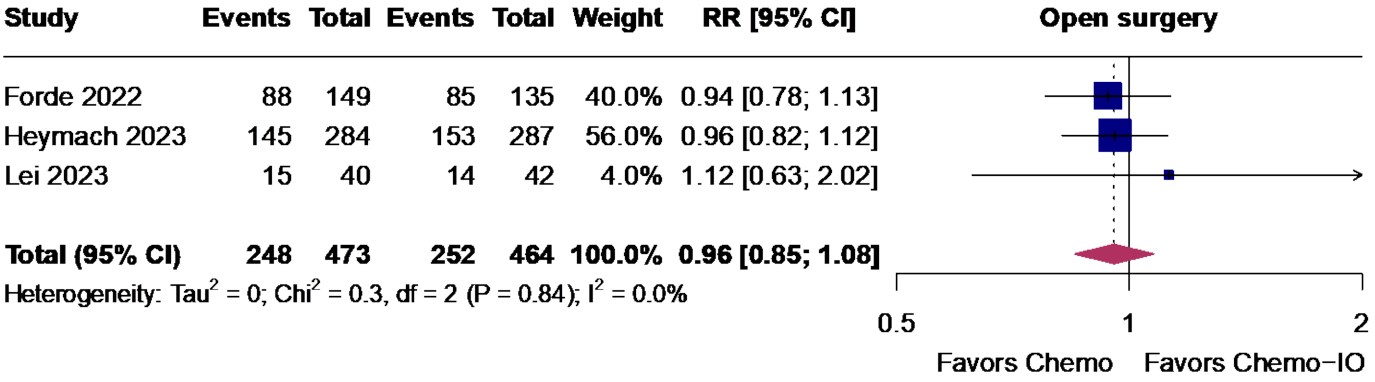

© 2024 Sorin M et al. *JAMA Oncology.*

eFigure 16. Pooled risk ratios across RCTs comparing neoadjuvant chemoimmunotherapy to neoadjuvant chemotherapy by the number of patients who had grade 3-4 treatment-related adverse events.

Study	Chemo-IO Events	Chemo-IO Total	Chemo Events	Chemo Total	Weight	RR [95% CI]
Forde 2022	59	179	65	179	7.1%	0.91 [0.68; 1.21]
Wakelee 2023	158	397	143	400	18.0%	1.11 [0.93; 1.33]
Provencio 2023	11	57	3	29	0.4%	1.87 [0.56; 6.17]
Heymach 2023	130	401	131	398	14.5%	0.98 [0.81; 1.20]
Lu 2023	122	202	105	202	19.1%	1.16 [0.98; 1.38]
Cascone 2023	62	229	53	232	5.7%	1.19 [0.86; 1.63]
Lei 2023	11	47	5	43	0.6%	2.01 [0.76; 5.32]
Yue 2023	157	226	148	227	34.7%	1.07 [0.94; 1.21]
**Total (95% CI)**	**710**	**1738**	**653**	**1710**	**100.0%**	**1.08 [1.00; 1.17]**

Heterogeneity: $Tau^2 = 0$; $Chi^2 = 5.8$, df = 7 (P = 0.56); $I^2 = 0.0\%$

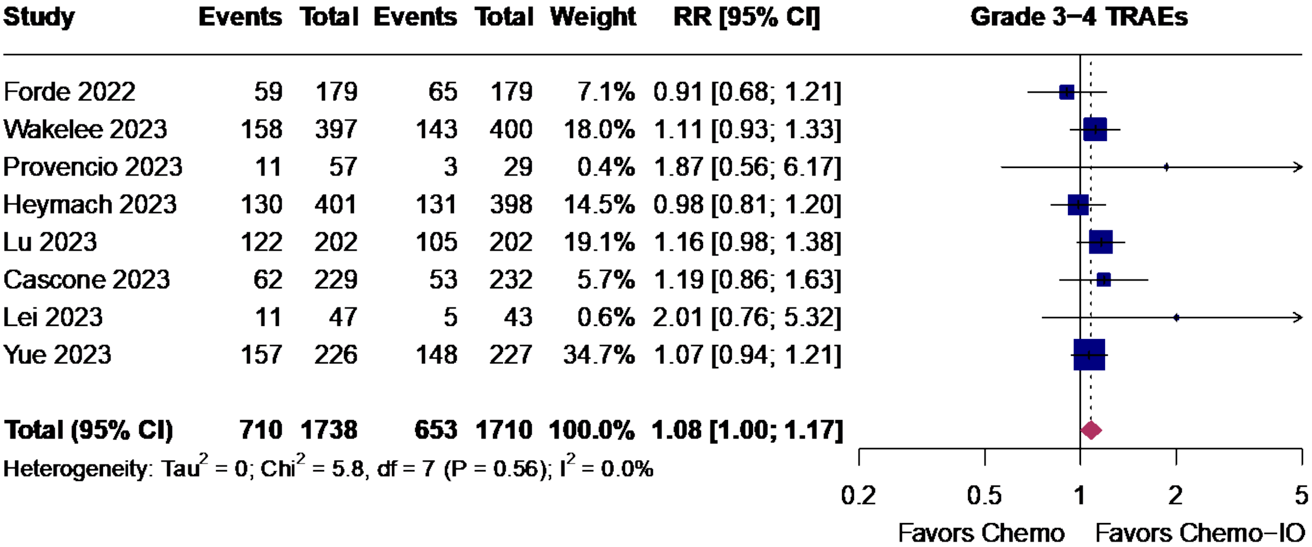

eFigure 17. Pooled risk ratios across RCTs comparing neoadjuvant chemoimmunotherapy to neoadjuvant chemotherapy by the number of patients who had grade 5 treatment-related adverse events.

Study	Chemo-IO Events	Chemo-IO Total	Chemo Events	Chemo Total	Weight	RR [95% CI]
Forde 2022	0	179	3	179	6.5%	0.14 [0.01; 2.75]
Wakelee 2023	3	397	3	400	22.2%	1.01 [0.20; 4.96]
Heymach 2023	7	401	2	398	23.0%	3.47 [0.73; 16.62]
Lu 2023	6	202	4	202	36.1%	1.50 [0.43; 5.24]
Cascone 2023	2	229	0	232	6.1%	5.07 [0.24; 104.93]
Lei 2023	0	47	0	43	0.0%	
Yue 2023	2	226	0	227	6.1%	5.02 [0.24; 104.02]
**Total (95% CI)**	**20**	**1681**	**12**	**1681**	**100.0%**	**1.66 [0.78; 3.52]**

Heterogeneity: $Tau^2 < 0.1$; $Chi^2 = 4.9$, df = 5 (P = 0.42); $I^2 = 0.0\%$

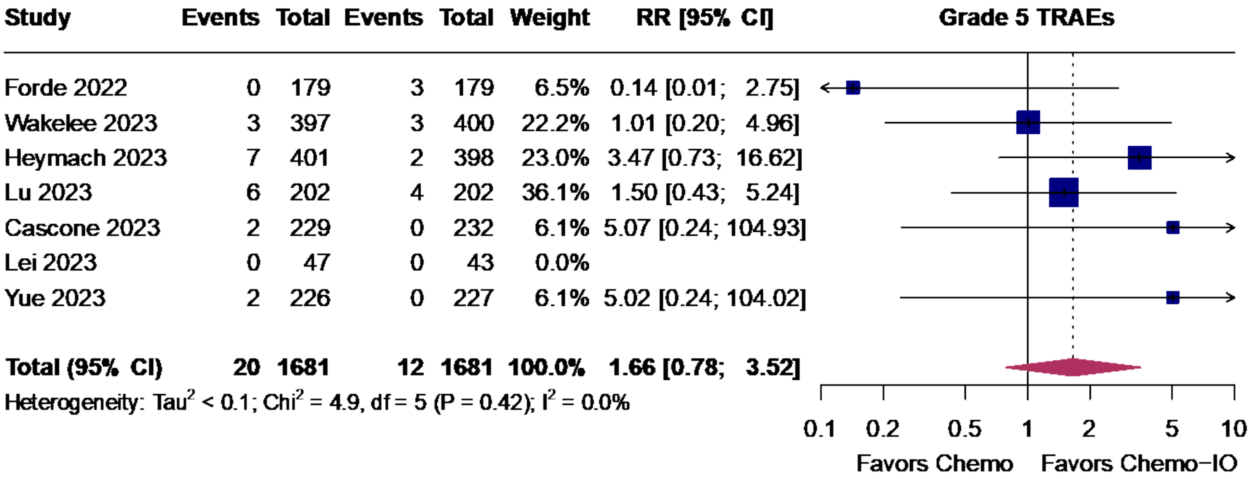

© 2024 Sorin M et al. *JAMA Oncology.*

eFigure 18. Pooled risk ratios across RCTs comparing neoadjuvant chemoimmunotherapy to neoadjuvant chemotherapy by the number of patients who had treatment-related adverse events.

	Chemo–IO		Chemo			
**Study**	**Events**	**Total**	**Events**	**Total**	**Weight**	**RR [95% CI]**
Forde 2022	145	179	156	179	1.7%	0.93 [0.85; 1.02]
Wakelee 2023	379	397	374	400	12.1%	1.02 [0.99; 1.06]
Provencio 2023	50	57	26	29	0.6%	0.98 [0.84; 1.14]
Heymach 2023	348	401	321	398	3.7%	1.08 [1.01; 1.14]
Lu 2023	201	202	199	202	35.0%	1.01 [0.99; 1.03]
Cascone 2023	197	229	197	232	2.4%	1.01 [0.94; 1.09]
Lei 2023	41	47	40	43	0.7%	0.94 [0.82; 1.08]
Yue 2023	224	226	225	227	43.8%	1.00 [0.98; 1.02]
**Total (95% CI)**	**1585**	**1738**	**1538**	**1710**	**100.0%**	**1.01 [1.00; 1.02]**

Heterogeneity: $Tau^2 < 0.1$; $Chi^2 = 10.0$, df = 7 (P = 0.19); $I^2 = 30.3\%$

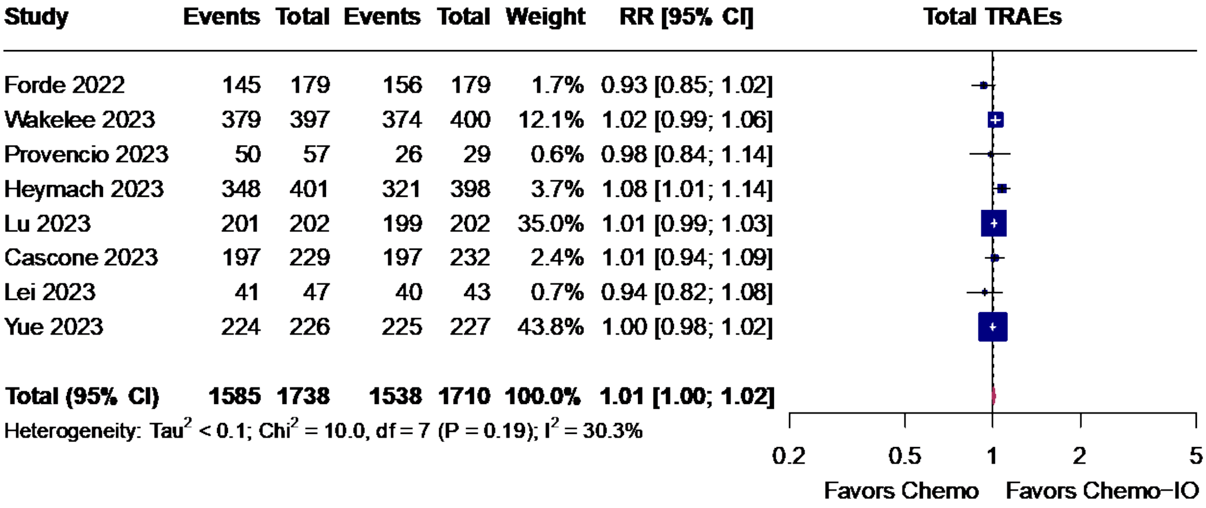

eFigure 19. Pooled risk ratios across RCTs comparing neoadjuvant chemoimmunotherapy to neoadjuvant chemotherapy by the number of patients who had grade 3-4 surgery-related adverse events.

	Chemo–IO		Chemo			
**Study**	**Events**	**Total**	**Events**	**Total**	**Weight**	**RR [95% CI]**
Forde 2022	17	149	20	135	12.3%	0.77 [0.42; 1.41]
Wakelee 2023	75	325	63	317	50.8%	1.16 [0.86; 1.56]
Lu 2023	36	166	28	148	23.0%	1.15 [0.74; 1.78]
Cascone 2023	21	178	21	178	13.9%	1.00 [0.57; 1.76]
Lei 2023	0	40	0	40	0.0%	
**Total (95% CI)**	**149**	**858**	**132**	**818**	**100.0%**	**1.08 [0.87; 1.33]**

Heterogeneity: $Tau^2 = 0$; $Chi^2 = 1.6$, df = 3 (P = 0.66); $I^2 = 0.0\%$

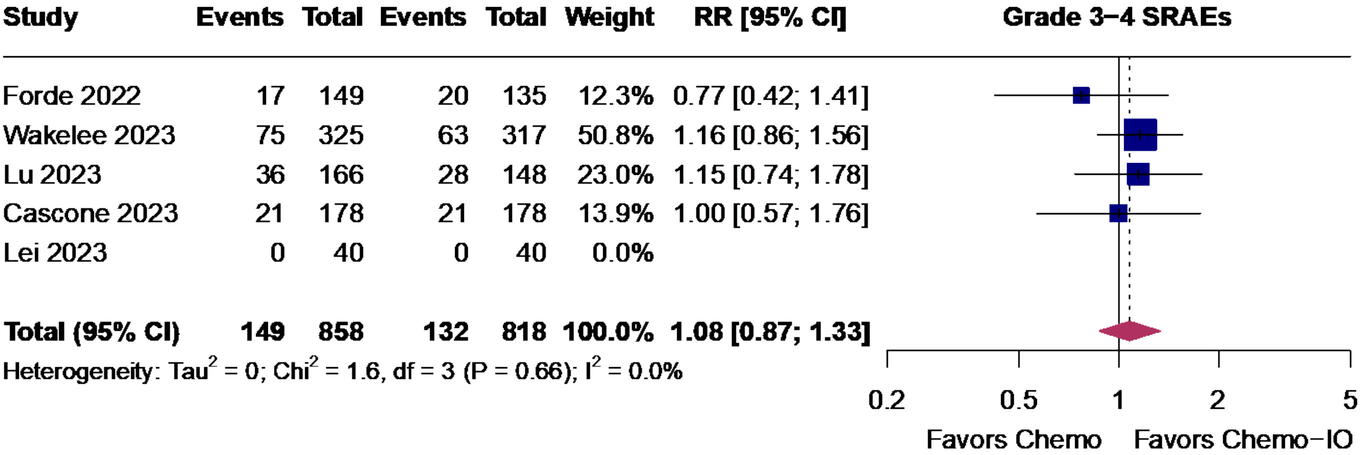

© 2024 Sorin M et al. *JAMA Oncology.*

eFigure 20. Pooled risk ratios across RCTs comparing neoadjuvant chemoimmunotherapy to neoadjuvant chemotherapy by the number of patients who had grade 5 surgery-related adverse events.

	Chemo−IO		Chemo			
**Study**	**Events**	**Total**	**Events**	**Total**	**Weight**	**RR [95% CI]**
Forde 2022	2	149	0	135	7.4%	4.53 [0.22; 93.56]
Wakelee 2023	9	325	5	317	58.1%	1.76 [0.59; 5.18]
Provencio 2023	1	53	0	20	6.8%	1.15 [0.05; 27.09]
Lu 2023	0	166	2	148	7.4%	0.18 [0.01; 3.69]
Cascone 2023	3	178	1	178	13.4%	3.00 [0.32; 28.57]
Lei 2023	1	40	0	40	6.8%	3.00 [0.13; 71.48]
**Total (95% CI)**	**16**	**911**	**8**	**838**	**100.0%**	**1.72 [0.75; 3.93]**

Heterogeneity: Tau^2 = 0; Chi^2 = 3.0, df = 5 (P = 0.71); I^2 = 0.0%

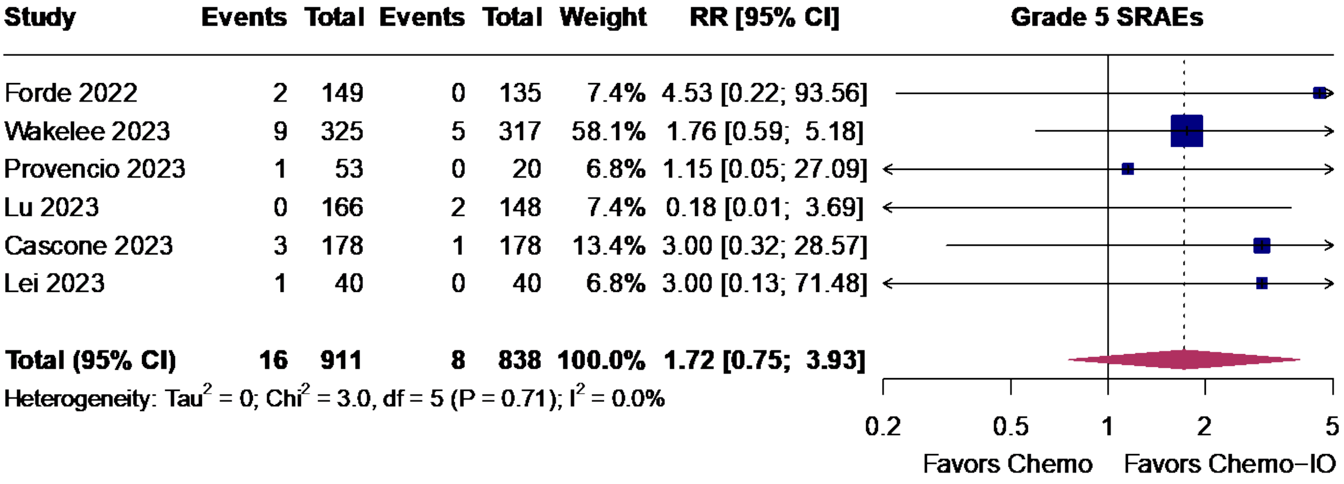

eFigure 21. Pooled risk ratios across RCTs comparing neoadjuvant chemoimmunotherapy to neoadjuvant chemotherapy by the number of patients who had surgery-related adverse events.

	Chemo−IO		Chemo			
**Study**	**Events**	**Total**	**Events**	**Total**	**Weight**	**RR [95% CI]**
Forde 2022	62	149	63	135	7.7%	0.89 [0.69; 1.16]
Wakelee 2023	231	325	226	317	54.5%	1.00 [0.90; 1.10]
Lu 2023	124	166	104	148	28.1%	1.06 [0.93; 1.22]
Cascone 2023	73	178	69	178	8.1%	1.06 [0.82; 1.37]
Lei 2023	16	40	14	40	1.6%	1.14 [0.65; 2.02]
**Total (95% CI)**	**506**	**858**	**476**	**818**	**100.0%**	**1.01 [0.94; 1.09]**

Heterogeneity: Tau^2 = 0; Chi^2 = 1.8, df = 4 (P = 0.78); I^2 = 0.0%

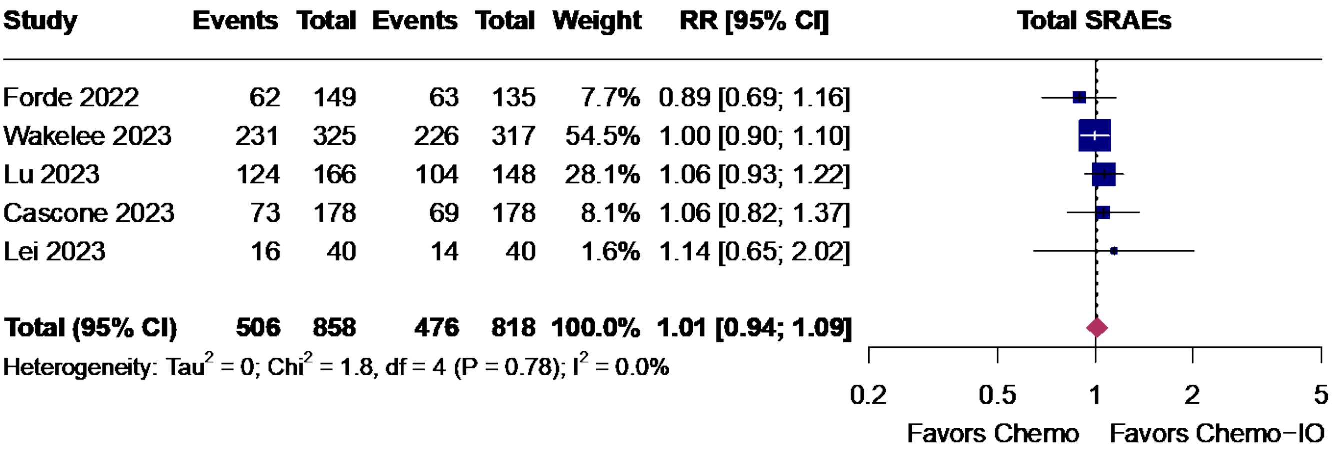

© 2024 Sorin M et al. *JAMA Oncology.*

eFigure 22. Pooled risk ratios across RCTs comparing neoadjuvant chemoimmunotherapy to neoadjuvant chemotherapy by the number of patients who did not receive surgery.

	Chemo–IO		Chemo				
Study	Events	Total	Events	Total	Weight	RR [95% CI]	Did not receive surgery
Forde 2022	30	179	44	179	11.1%	0.68 [0.45; 1.03]	
Wakelee 2023	72	397	83	400	20.6%	0.87 [0.66; 1.16]	
Heymach 2023	82	366	87	374	22.8%	0.96 [0.74; 1.26]	
Provencio 2023	4	57	9	29	1.8%	0.23 [0.08; 0.67]	
Lu 2023	36	202	54	202	13.2%	0.67 [0.46; 0.97]	
Cascone 2023	51	229	54	232	15.8%	0.96 [0.68; 1.34]	
Lei 2023	7	47	5	47	1.9%	1.40 [0.48; 4.10]	
Yue 2023	36	226	54	227	12.9%	0.67 [0.46; 0.98]	
**Total (95% CI)**	**318**	**1703**	**390**	**1690**	**100.0%**	**0.81 [0.70; 0.94]**	

Heterogeneity: $Tau^2 < 0.1$; $Chi^2 = 11.8$, df = 7 (P = 0.11); $I^2 = 40.5\%$

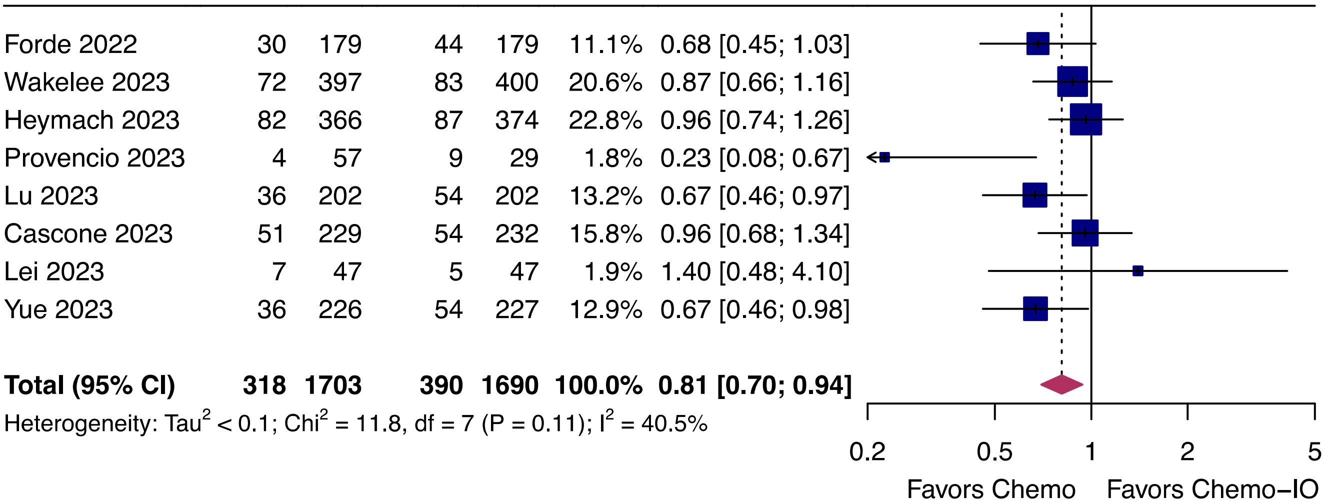

eFigure 23. Pooled risk ratios across RCTs comparing neoadjuvant chemoimmunotherapy to neoadjuvant chemotherapy by the number of patients who had progression precluding surgery.

	Chemo–IO		Chemo				
Study	Events	Total	Events	Total	Weight	RR [95% CI]	Disease progression precluding surgery
Forde 2022	12	179	17	179	16.5%	0.71 [0.35; 1.43]	
Wakelee 2023	10	397	17	400	15.4%	0.59 [0.27; 1.28]	
Heymach 2023	35	366	42	374	22.8%	0.85 [0.56; 1.30]	
Provencio 2023	0	57	4	29	2.1%	0.06 [0.00; 1.02]	
Lu 2023	5	202	31	202	12.7%	0.16 [0.06; 0.41]	
Cascone 2023	13	229	22	232	17.5%	0.60 [0.31; 1.16]	
Lei 2023	0	47	1	47	1.8%	0.33 [0.01; 7.98]	
Yue 2023	5	226	12	227	11.2%	0.42 [0.15; 1.17]	
**Total (95% CI)**	**80**	**1703**	**146**	**1690**	**100.0%**	**0.51 [0.33; 0.79]**	

Heterogeneity: $Tau^2 = 0.2$; $Chi^2 = 13.7$, df = 7 (P = 0.06); $I^2 = 49.0\%$

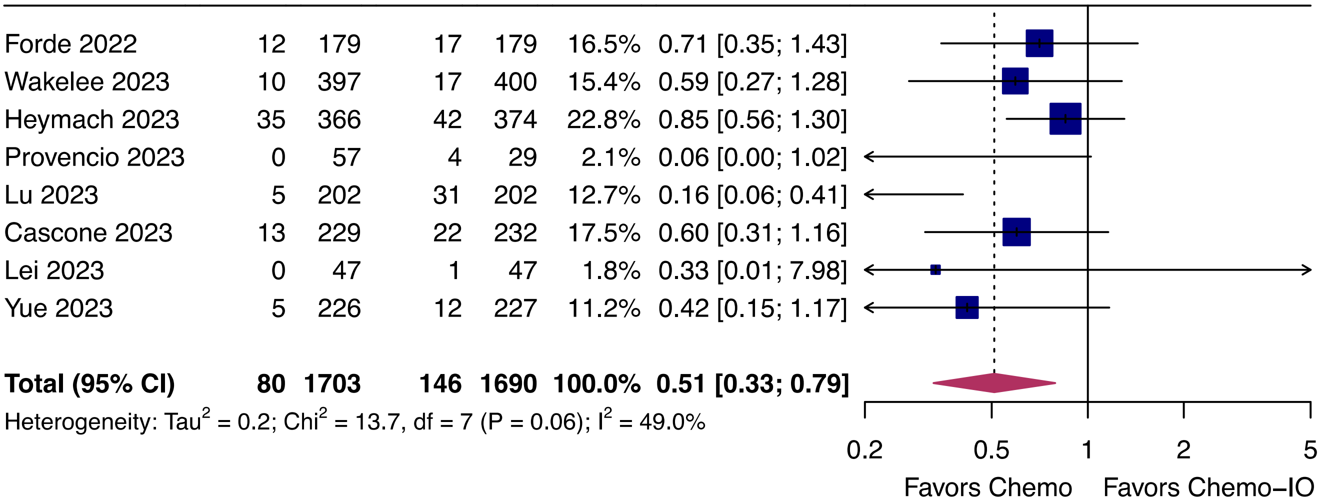

© 2024 Sorin M et al. *JAMA Oncology.*

eFigure 24. Pooled risk ratios across RCTs comparing neoadjuvant chemoimmunotherapy to neoadjuvant chemotherapy by the number of patients who had adverse events precluding surgery.

Study	Chemo–IO Events	Chemo–IO Total	Chemo Events	Chemo Total	Weight	RR [95% CI]
Forde 2022	2	179	1	179	7.0%	2.00 [0.18; 21.86]
Wakelee 2023	8	397	1	400	9.3%	8.06 [1.01; 64.15]
Heymach 2023	7	366	5	374	30.8%	1.43 [0.46; 4.47]
Provencio 2023	1	57	1	29	5.3%	0.51 [0.03; 7.84]
Lu 2023	6	202	0	202	4.8%	13.00 [0.74; 229.24]
Cascone 2023	7	229	4	232	27.0%	1.77 [0.53; 5.97]
Lei 2023	0	47	0	47	0.0%	
Yue 2023	6	226	2	227	15.8%	3.01 [0.61; 14.77]
**Total (95% CI)**	**37**	**1703**	**14**	**1690**	**100.0%**	**2.16 [1.15; 4.06]**

Heterogeneity: $Tau^2 < 0.1$; $Chi^2 = 4.9$, df = 6 (P = 0.56); $I^2 = 0.0\%$

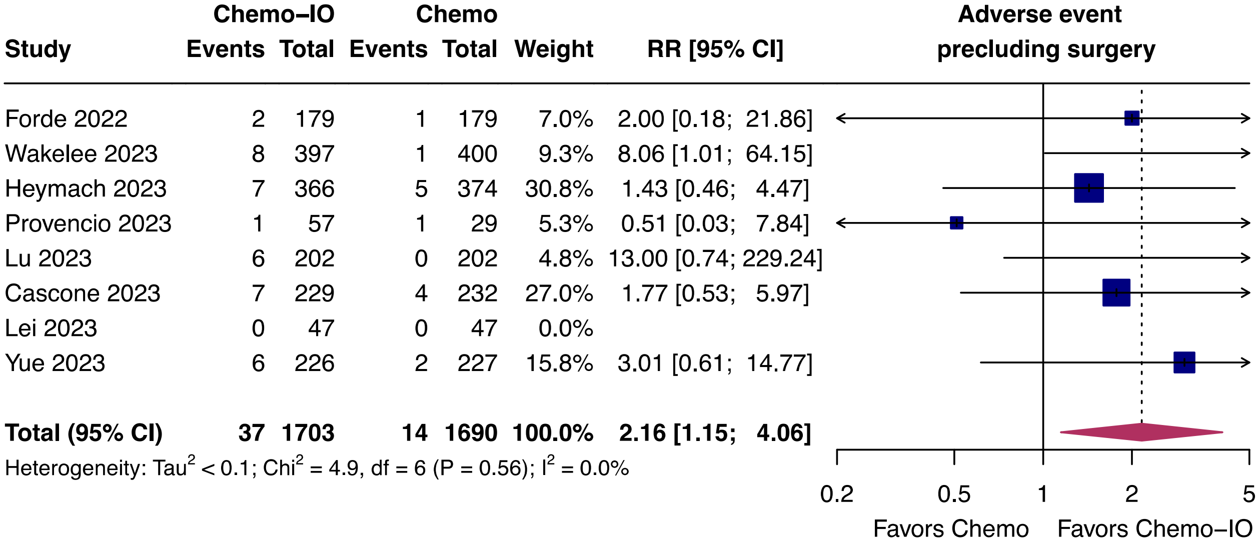

eFigure 25. Pooled risk ratios across RCTs comparing neoadjuvant chemoimmunotherapy to neoadjuvant chemotherapy by the number of patient refusals precluding surgery.

Study	Chemo–IO Events	Chemo–IO Total	Chemo Events	Chemo Total	Weight	RR [95% CI]
Forde 2022	9	179	11	179	12.2%	0.82 [0.35; 1.93]
Wakelee 2023	4	397	6	400	5.6%	0.67 [0.19; 2.36]
Heymach 2023	12	366	17	374	17.0%	0.72 [0.35; 1.49]
Lu 2023	18	202	13	202	19.0%	1.38 [0.70; 2.75]
Cascone 2023	8	229	18	232	13.5%	0.45 [0.20; 1.01]
Lei 2023	2	47	2	47	2.4%	1.00 [0.15; 6.81]
Yue 2023	20	226	28	227	30.2%	0.72 [0.42; 1.24]
**Total (95% CI)**	**73**	**1646**	**95**	**1661**	**100.0%**	**0.78 [0.58; 1.05]**

Heterogeneity: $Tau^2 = 0$; $Chi^2 = 4.7$, df = 6 (P = 0.58); $I^2 = 0.0\%$

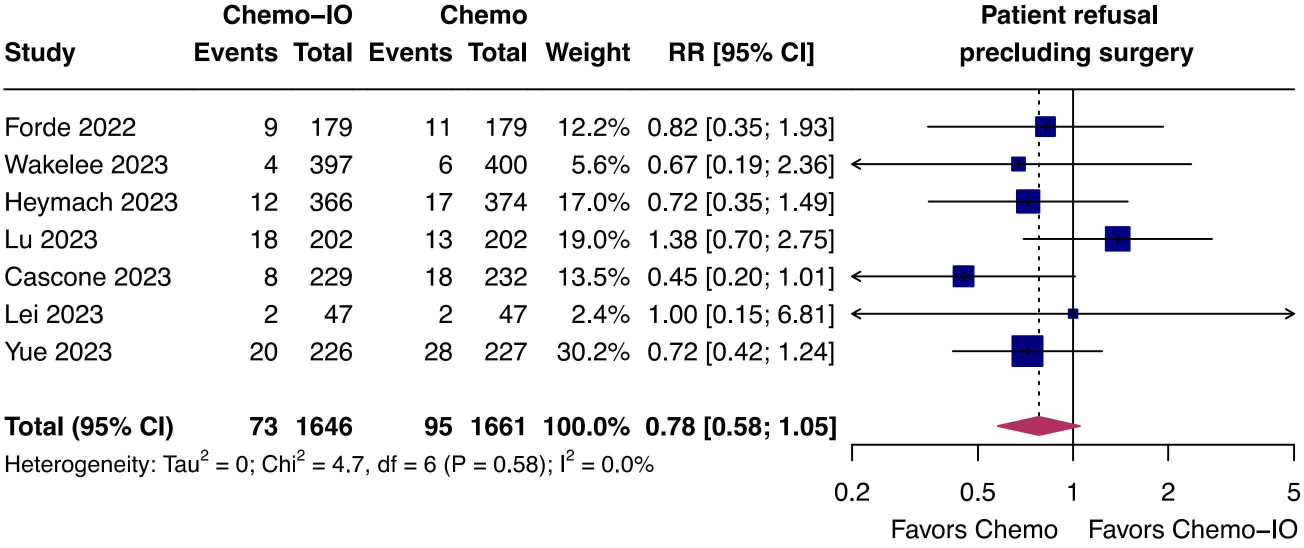

© 2024 Sorin M et al. *JAMA Oncology.*

eFigure 26. Pooled risk ratios across RCTs comparing neoadjuvant chemoimmunotherapy to neoadjuvant chemotherapy by the number of patients with other reasons precluding surgery.

	Chemo–IO		Chemo			
**Study**	**Events**	**Total**	**Events**	**Total**	**Weight**	**RR [95% CI]**
Forde 2022	4	179	7	179	9.7%	0.57 [0.17; 1.92]
Wakelee 2023	1	397	4	400	3.3%	0.25 [0.03; 2.24]
Heymach 2023	36	366	30	374	36.1%	1.23 [0.77; 1.95]
Lu 2023	7	202	10	202	14.6%	0.70 [0.27; 1.80]
Cascone 2023	15	229	16	232	23.5%	0.95 [0.48; 1.88]
Lei 2023	0	47	0	47	0.0%	
Yue 2023	5	226	12	227	12.8%	0.42 [0.15; 1.17]
**Total (95% CI)**	**68**	**1646**	**79**	**1661**	**100.0%**	**0.82 [0.54; 1.23]**

Heterogeneity: $Tau^2 < 0.1$; $Chi^2 = 6.0$, df = 5 (P = 0.31); $I^2 = 16.5\%$

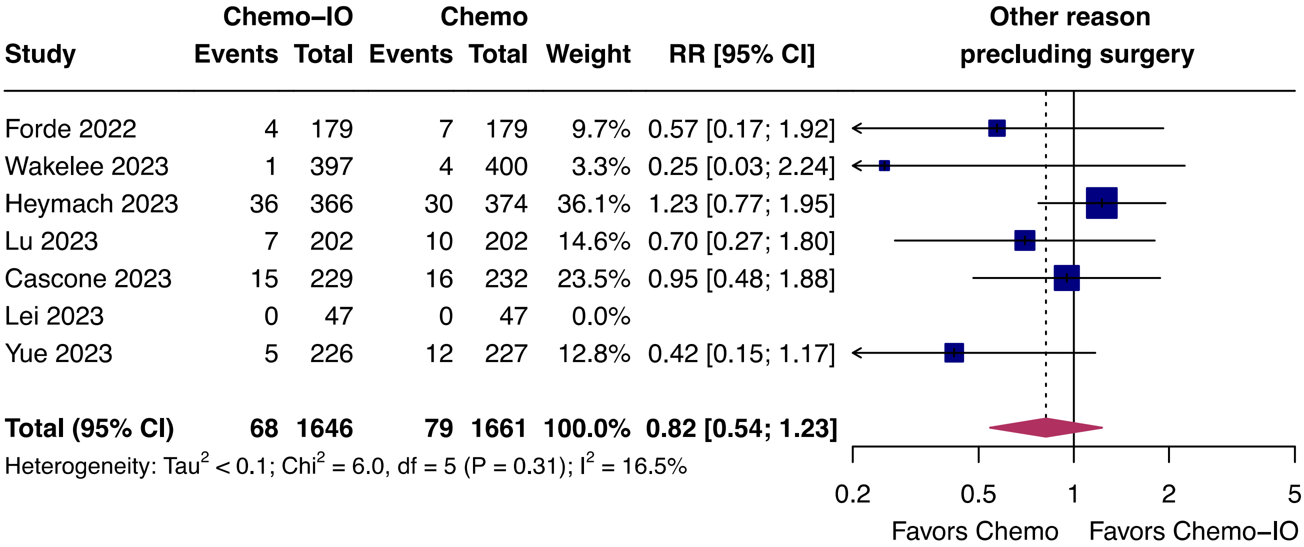

© 2024 Sorin M et al. *JAMA Oncology.*

eFigure 27. Pooled proportions across all studies comparing neoadjuvant chemoimmunotherapy to neoadjuvant chemotherapy by MPR.

Author	Events	Total	Percentage [95%CI]
**Chemo**			
Cascone 2018	7	47	14.89 [6.20; 28.31]
Chaft 2016	6	40	15.00 [5.71; 29.84]
Dy 2014	8	38	21.05 [9.55; 37.32]
Forde 2022	16	179	8.94 [5.20; 14.11]
Gu 2022	2	19	10.53 [1.30; 33.14]
Li 2015	15	93	16.13 [9.32; 25.20]
Mittal 2021	0	37	0.00 [0.00; 9.49]
Provencio 2023	4	29	13.79 [3.89; 31.66]
Rothschild 2021	34	67	50.75 [38.24; 63.18]
Wakelee 2023	44	400	11.00 [8.11; 14.48]
Xiong 2019	3	16	18.75 [4.05; 45.65]
Lei 2023	7	47	14.89 [6.20; 28.31]
Lu 2023	98	202	48.51 [41.44; 55.63]
Cascone 2023	28	232	12.07 [8.17; 16.97]
Heymach 2023	46	374	12.30 [9.15; 16.06]
Yue 2023	34	227	14.98 [10.60; 20.30]
**Total (95% CI)**	**352**	**2047**	**16.80 [11.96; 23.08]**
Heterogeneity: $Tau^2 = 0.5$; $Chi^2 = 188.9$, df = 15 ($P < 0.01$); $I^2 = 92.1\%$			
**Chemo_IO**			
Cascone 2023	7	22	31.82 [13.86; 54.87]
Cascone 2023	11	22	50.00 [28.22; 71.78]
Cui 2022	20	30	66.67 [47.19; 82.71]
Forde 2022	66	179	36.87 [29.80; 44.39]
Provencio 2020_2022	34	46	73.91 [58.87; 85.73]
Provencio 2023	30	57	52.63 [38.97; 66.02]
Rothschild 2021	34	67	50.75 [38.24; 63.18]
Shao 2023	7	29	24.14 [10.30; 43.54]
Shao 2023	12	31	38.71 [21.85; 57.81]
Shen 2021	24	37	64.86 [47.46; 79.79]
Shu 2020	17	30	56.67 [37.43; 74.54]
Sun 2022	10	20	50.00 [27.20; 72.80]
Tao 2023	31	55	56.36 [42.32; 69.70]
Tfayli 2020	1	15	6.67 [0.17; 31.95]
Wakelee 2023	120	397	30.23 [25.75; 35.01]
Yan 2022	19	37	51.35 [34.40; 68.08]
Zhang 2022	13	50	26.00 [14.63; 40.34]
Zhao 2021	20	33	60.61 [42.14; 77.09]
Zhu 2022	20	50	40.00 [26.41; 54.82]
Lei 2023	28	47	59.57 [44.27; 73.63]
Li 2023	18	39	46.15 [30.09; 62.82]
Lu 2023	17	202	8.42 [4.98; 13.13]
Cascone 2023	81	229	35.37 [29.19; 41.94]
Heymach 2023	122	366	33.33 [28.52; 38.42]
Yue 2023	127	226	56.19 [49.46; 62.77]
**Total (95% CI)**	**889**	**2316**	**43.67 [36.47; 51.15]**
Heterogeneity: $Tau^2 = 0.5$; $Chi^2 = 190.6$, df = 24 ($P < 0.01$); $I^2 = 87.4\%$			

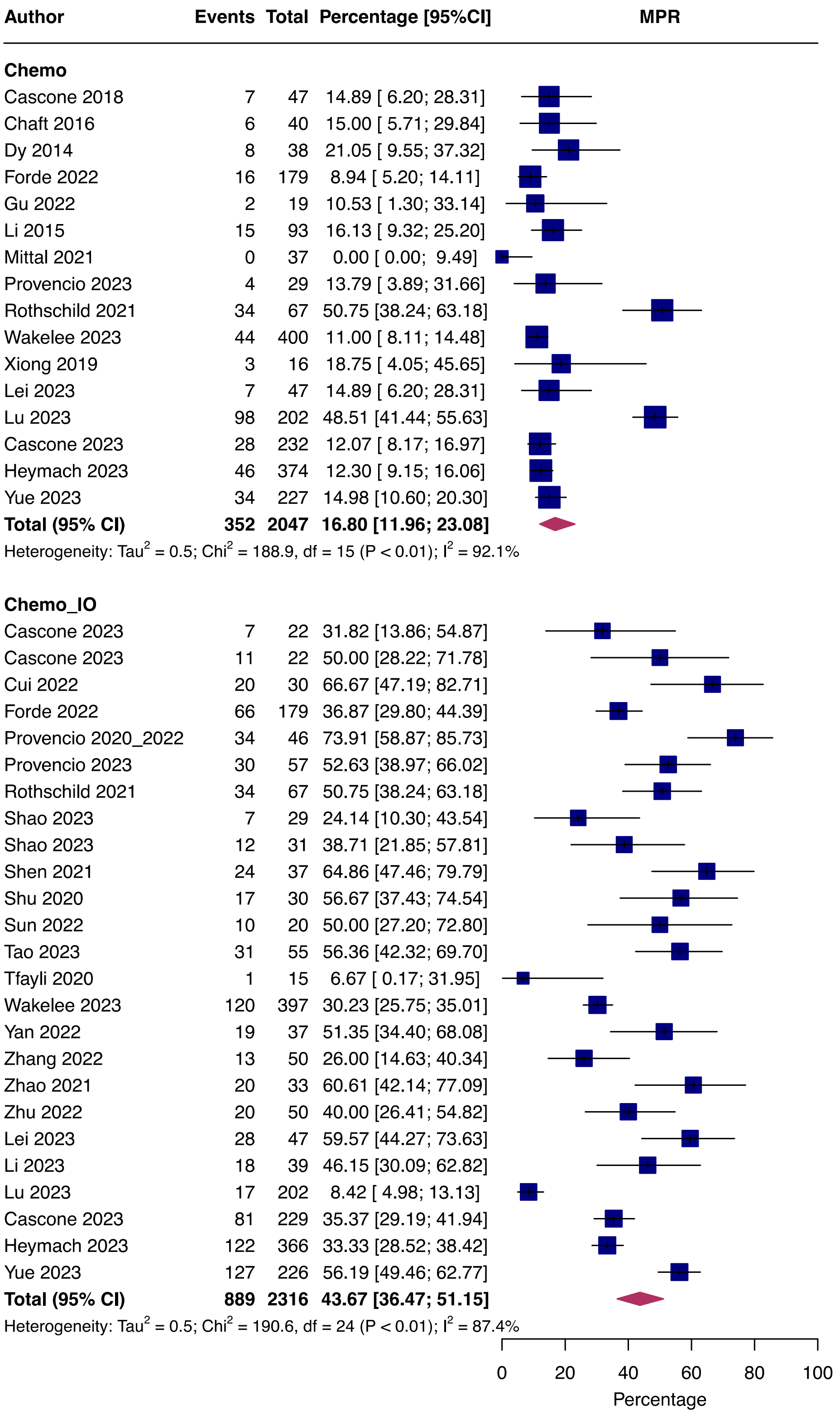

© 2024 Sorin M et al. *JAMA Oncology.*

eFigure 28. Pooled proportions across all studies comparing neoadjuvant chemoimmunotherapy to neoadjuvant chemotherapy by pCR.

Author	Events	Total	Percentage [95%CI]
**Chemo**			
Cascone 2018	5	47	10.64 [3.55; 23.10]
Dy 2014	0	38	0.00 [0.00; 9.25]
Forde 2022	4	179	2.23 [0.61; 5.62]
Gu 2022	1	19	5.26 [0.13; 26.03]
Hainsworth 2018	0	46	0.00 [0.00; 7.71]
Li 2015	7	93	7.53 [3.08; 14.90]
Mittal 2021	0	37	0.00 [0.00; 9.49]
Pless 2015	11	115	9.57 [4.87; 16.47]
Provencio 2023	2	29	6.90 [0.85; 22.77]
Romine 2019	0	25	0.00 [0.00; 13.72]
Rothschild 2021	10	67	14.93 [7.40; 25.74]
Wakelee 2023	16	400	4.00 [2.30; 6.41]
Westeel 2013	22	267	8.24 [5.24; 12.21]
Westeel 2013	16	261	6.13 [3.54; 9.76]
Xiong 2019	1	16	6.25 [0.16; 30.23]
Lei 2023	4	47	8.51 [2.37; 20.38]
Lu 2023	50	202	24.75 [18.96; 31.30]
Cascone 2023	11	232	4.74 [2.39; 8.33]
Heymach 2023	16	374	4.28 [2.46; 6.85]
Yue 2023	13	227	5.73 [3.08; 9.59]
**Total (95% CI)**	**189**	**2721**	**6.71 [4.83; 9.25]**
Heterogeneity: Tau^2 = 0.4; Chi^2 = 105.7, df = 19 ($P < 0.01$); I^2 = 82%			
**Chemo_IO**			
Cascone 2023	4	22	18.18 [5.19; 40.28]
Cascone 2023	4	22	18.18 [5.19; 40.28]
Cui 2022	16	30	53.33 [34.33; 71.66]
Forde 2022	43	179	24.02 [17.96; 30.96]
Hou 2022	6	11	54.55 [23.38; 83.25]
Provencio 2020_2022	26	46	56.52 [41.11; 71.07]
Provencio 2023	21	57	36.84 [24.45; 50.66]
Rothschild 2021	10	67	14.93 [7.40; 25.74]
Shao 2023	5	29	17.24 [5.85; 35.77]
Shao 2023	7	31	22.58 [9.59; 41.10]
Shen 2021	17	37	45.95 [29.49; 63.08]
Shu 2020	10	30	33.33 [17.29; 52.81]
Sun 2022	5	20	25.00 [8.66; 49.10]
Tao 2023	24	55	43.64 [30.30; 57.68]
Wakelee 2023	72	397	18.14 [14.47; 22.29]
Yan 2022	11	37	29.73 [15.87; 46.98]
Yang 2018	2	24	8.33 [1.03; 27.00]
Zhang 2022	6	50	12.00 [4.53; 24.31]
Zhao 2021	15	33	45.45 [28.11; 63.65]
Zhu 2022	10	50	20.00 [10.03; 33.72]
Lei 2023	14	47	29.79 [17.34; 44.89]
Lu 2023	2	202	0.99 [0.12; 3.53]
Cascone 2023	58	229	25.33 [19.83; 31.48]
Heymach 2023	63	366	17.21 [13.49; 21.48]
Yue 2023	92	226	40.71 [34.24; 47.42]
**Total (95% CI)**	**543**	**2297**	**26.31 [20.42; 33.18]**
Heterogeneity: Tau^2 = 0.6; Chi^2 = 152.7, df = 24 ($P < 0.01$); I^2 = 84.3%			

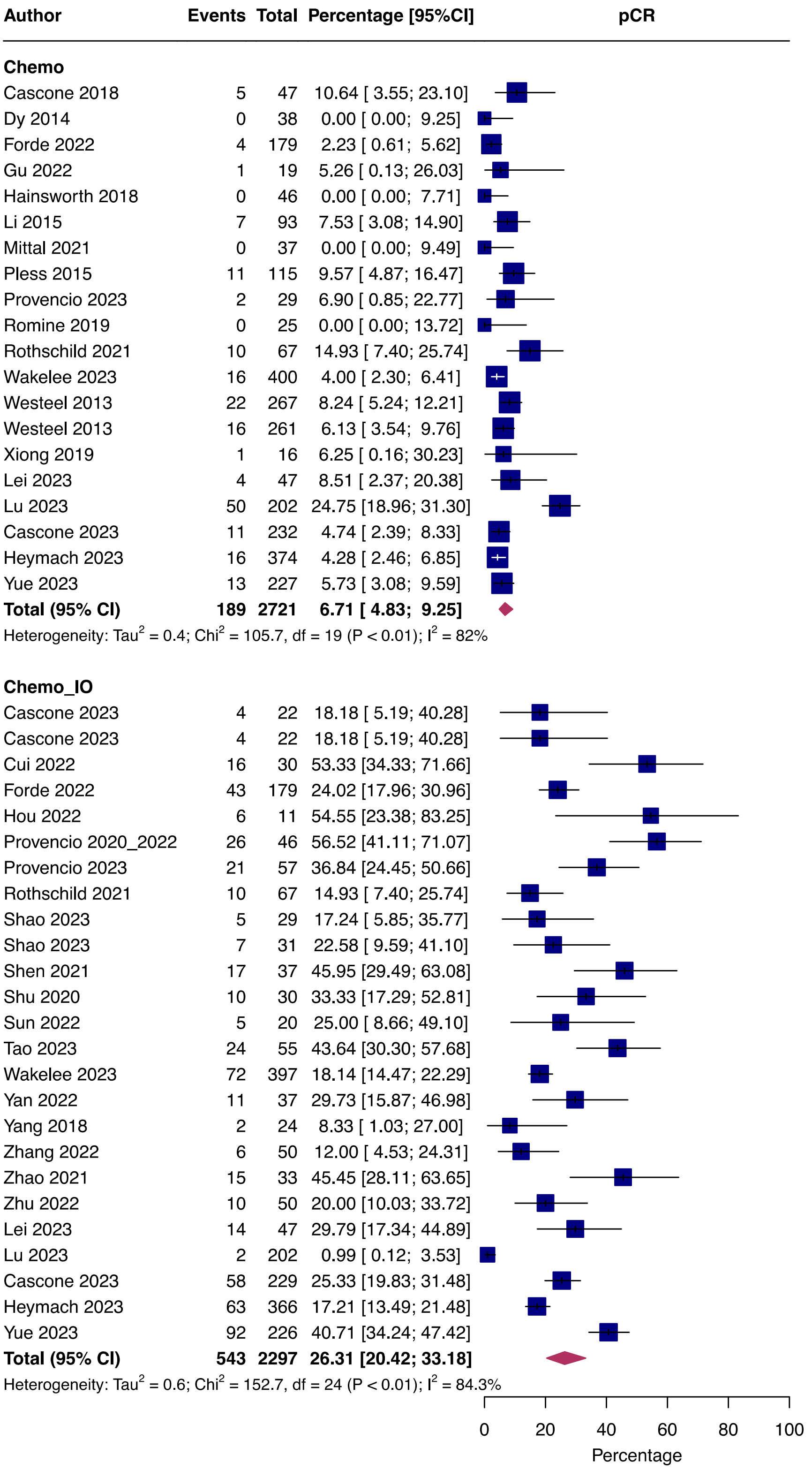

© 2024 Sorin M et al. *JAMA Oncology.*

eFigure 29. Pooled proportions across all studies comparing neoadjuvant chemoimmunotherapy to neoadjuvant chemotherapy by complete response per RECIST.

Author	Events	Total	Percentage [95%CI]
**Chemo**			
Cascone 2018	0	47	0.00 [0.00; 7.55]
Dy 2014	0	38	0.00 [0.00; 9.25]
Forde 2022	3	179	1.68 [0.35; 4.82]
Hainsworth 2018	0	46	0.00 [0.00; 7.71]
Li 2015	7	93	7.53 [3.08; 14.90]
Mittal 2021	4	37	10.81 [3.03; 25.42]
Pless 2015	2	115	1.74 [0.21; 6.14]
Romine 2019	0	25	0.00 [0.00; 13.72]
Rothschild 2021	2	67	2.99 [0.36; 10.37]
Xiong 2019	0	16	0.00 [0.00; 20.59]
Lei 2023	4	47	8.51 [2.37; 20.38]
Heymach 2023	1	374	0.27 [0.01; 1.48]
**Total (95% CI)**	**23**	**1084**	**2.95 [1.50; 5.71]**
Heterogeneity: Tau^2 = 0.7; Chi^2 = 23.6, df = 11 (P = 0.01); I^2 = 53.4%			
**Chemo_IO**			
Cascone 2023	0	22	0.00 [0.00; 15.44]
Cascone 2023	0	22	0.00 [0.00; 15.44]
Cui 2022	2	30	6.67 [0.82; 22.07]
Forde 2022	1	179	0.56 [0.01; 3.07]
Hou 2022	0	11	0.00 [0.00; 28.49]
Provencio 2020_2022	2	46	4.35 [0.53; 14.84]
Rothschild 2021	4	67	5.97 [1.65; 14.59]
Shao 2023	0	29	0.00 [0.00; 11.94]
Shao 2023	0	31	0.00 [0.00; 11.22]
Shen 2021	10	37	27.03 [13.79; 44.12]
Shu 2020	0	30	0.00 [0.00; 11.57]
Sun 2022	0	20	0.00 [0.00; 16.84]
Tfayli 2020	1	15	6.67 [0.17; 31.95]
Yan 2022	1	37	2.70 [0.07; 14.16]
Yang 2018	0	24	0.00 [0.00; 14.25]
Zhang 2022	0	50	0.00 [0.00; 7.11]
Zhao 2021	3	33	9.09 [1.92; 24.33]
Zhu 2022	8	50	16.00 [7.17; 29.11]
Lei 2023	11	47	23.40 [12.30; 38.03]
Heymach 2023	4	366	1.09 [0.30; 2.77]
**Total (95% CI)**	**47**	**1146**	**4.78 [2.63; 8.53]**
Heterogeneity: Tau^2 = 1.1; Chi^2 = 69.3, df = 19 (P < 0.01); I^2 = 72.6%			

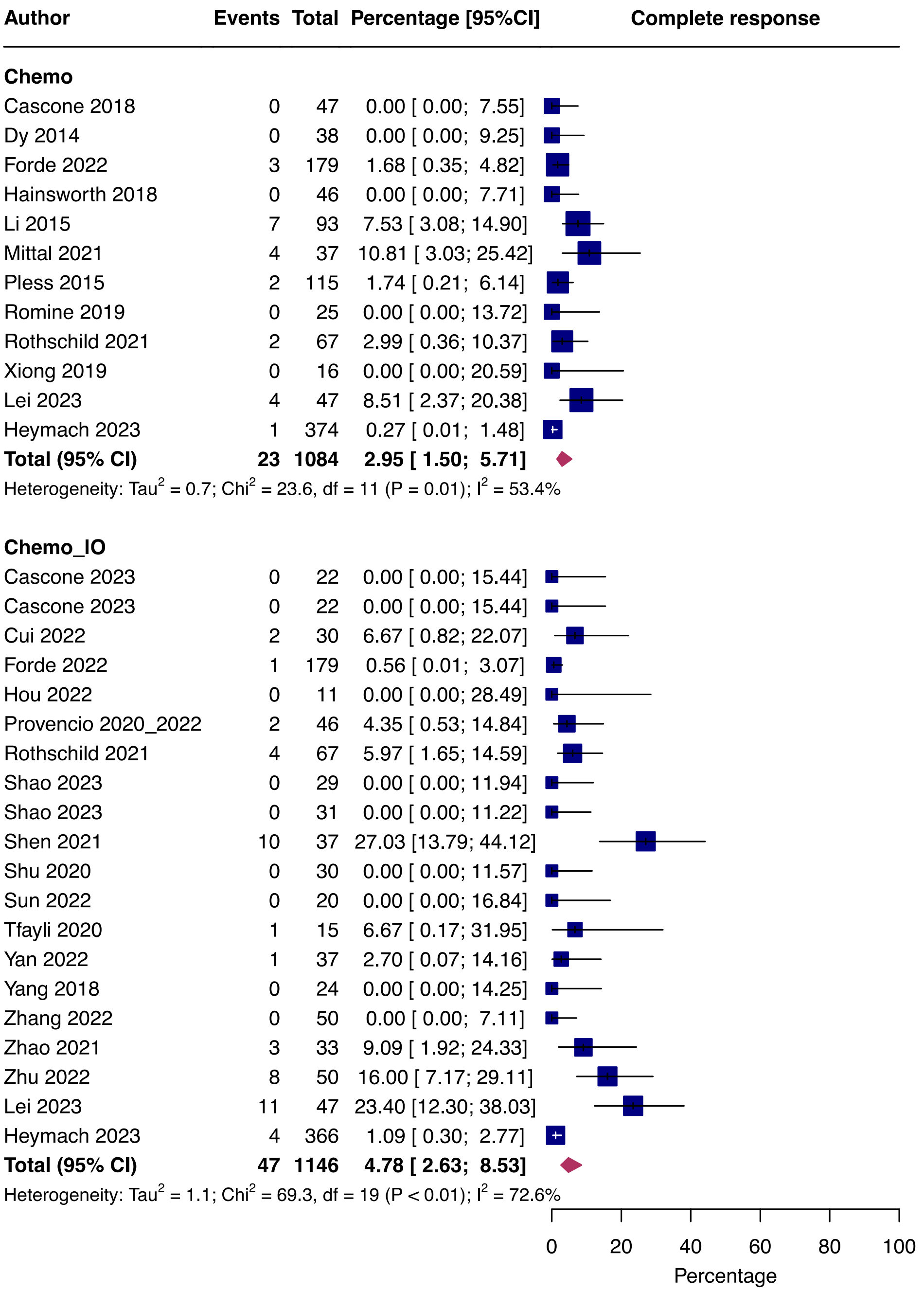

© 2024 Sorin M et al. *JAMA Oncology.*

eFigure 30. Pooled proportions across all studies comparing neoadjuvant chemoimmunotherapy to neoadjuvant chemotherapy by partial response per RECIST.

Author	Events	Total	Percentage [95%CI]
**Chemo**			
Cascone 2018	28	47	59.57 [44.27; 73.63]
Chaft 2016	24	40	60.00 [43.33; 75.14]
Dy 2014	11	38	28.95 [15.42; 45.90]
Forde 2022	64	179	35.75 [28.74; 43.25]
Gu 2022	9	19	47.37 [24.45; 71.14]
Hainsworth 2018	19	46	41.30 [27.00; 56.77]
Li 2015	68	93	73.12 [62.92; 81.79]
Mittal 2021	24	37	64.86 [47.46; 79.79]
Pless 2015	48	115	41.74 [32.61; 51.30]
Romine 2019	2	25	8.00 [0.98; 26.03]
Rothschild 2021	27	67	40.30 [28.49; 53.00]
Xiong 2019	3	16	18.75 [4.05; 45.65]
Lei 2023	20	47	42.55 [28.26; 57.82]
Heymach 2023	141	374	37.70 [32.77; 42.83]
**Total (95% CI)**	**488**	**1143**	**43.86 [35.20; 52.91]**
Heterogeneity: $Tau^2 = 0.4$; $Chi^2 = 69.9$, df = 13 ($P < 0.01$); $I^2 = 81.4\%$			
**Chemo_IO**			
Cascone 2023	9	22	40.91 [20.71; 63.65]
Cascone 2023	6	22	27.27 [10.73; 50.22]
Cui 2022	25	30	83.33 [65.28; 94.36]
Forde 2022	95	179	53.07 [45.48; 60.56]
Hou 2022	10	11	90.91 [58.72; 99.77]
Provencio 2020_2022	33	46	71.74 [56.54; 84.01]
Rothschild 2021	32	67	47.76 [35.40; 60.33]
Shao 2023	13	29	44.83 [26.45; 64.31]
Shao 2023	16	31	51.61 [33.06; 69.85]
Shen 2021	22	37	59.46 [42.10; 75.25]
Shu 2020	19	30	63.33 [43.86; 80.07]
Sun 2022	14	20	70.00 [45.72; 88.11]
Tfayli 2020	2	15	13.33 [1.66; 40.46]
Yan 2022	25	37	67.57 [50.21; 81.99]
Yang 2018	14	24	58.33 [36.64; 77.89]
Zhang 2022	23	50	46.00 [31.81; 60.68]
Zhao 2021	26	33	78.79 [61.09; 91.02]
Zhu 2022	30	50	60.00 [45.18; 73.59]
Lei 2023	20	47	42.55 [28.26; 57.82]
Heymach 2023	202	366	55.19 [49.94; 60.36]
**Total (95% CI)**	**636**	**1146**	**56.27 [49.65; 62.68]**
Heterogeneity: $Tau^2 = 0.2$; $Chi^2 = 52.7$, df = 19 ($P < 0.01$); $I^2 = 63.9\%$			

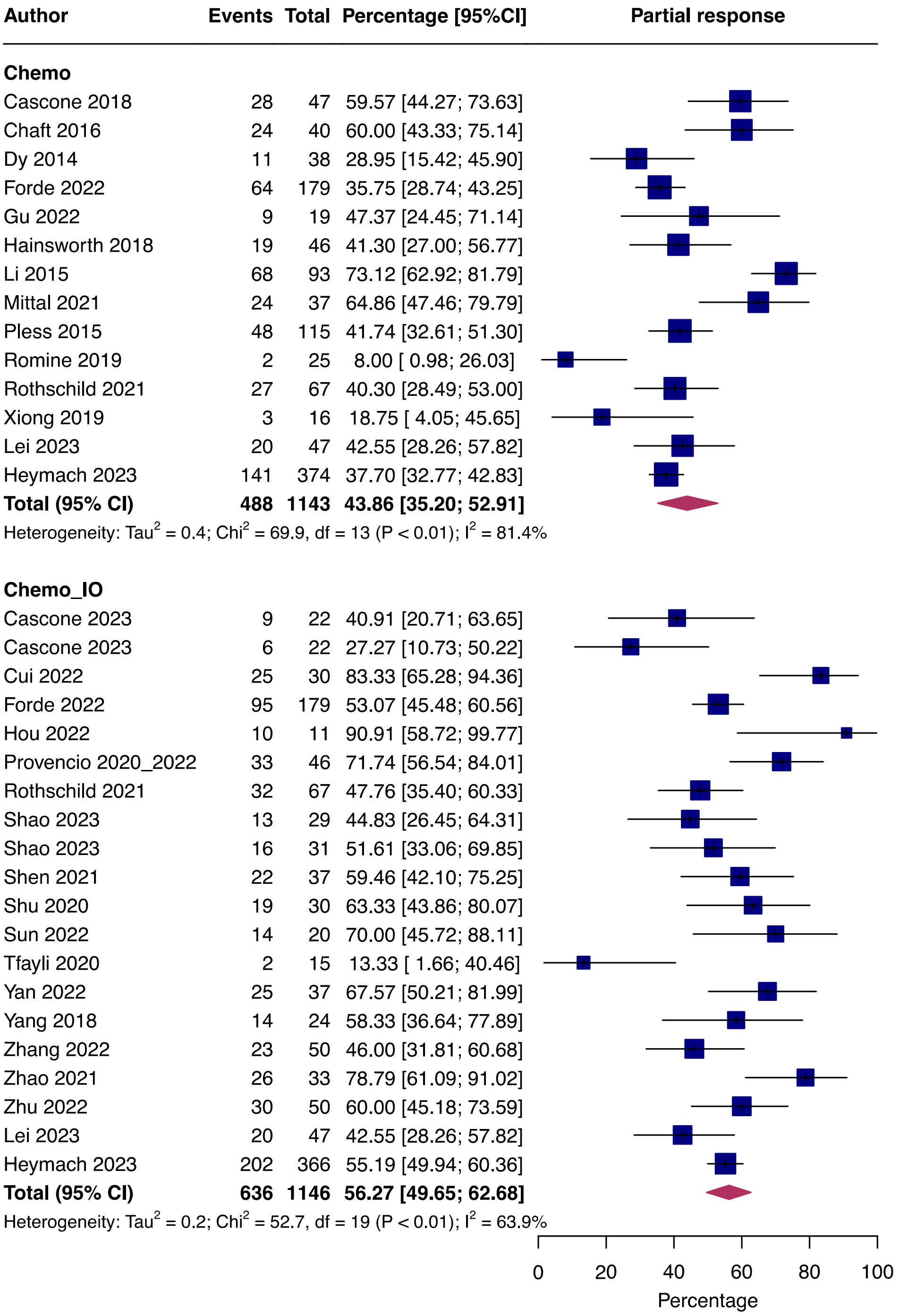

© 2024 Sorin M et al. *JAMA Oncology.*

eFigure 31. Pooled proportions across all studies comparing neoadjuvant chemoimmunotherapy to neoadjuvant chemotherapy by stable disease per RECIST.

Author	Events	Total	Percentage [95%CI]
**Chemo**			
Cascone 2018	18	47	38.30 [24.51; 53.62]
Dy 2014	23	38	60.53 [43.39; 75.96]
Forde 2022	88	179	49.16 [41.63; 56.73]
Gu 2022	9	19	47.37 [24.45; 71.14]
Hainsworth 2018	22	46	47.83 [32.89; 63.05]
Li 2015	14	93	15.05 [8.48; 23.97]
Mittal 2021	5	37	13.51 [4.54; 28.77]
Pless 2015	45	115	39.13 [30.16; 48.67]
Romine 2019	14	25	56.00 [34.93; 75.60]
Rothschild 2021	30	67	44.78 [32.60; 57.42]
Xiong 2019	12	16	75.00 [47.62; 92.73]
Lei 2023	15	47	31.91 [19.09; 47.12]
Heymach 2023	189	374	50.53 [45.35; 55.71]
**Total (95% CI)**	**484**	**1103**	**42.21 [33.16; 51.82]**
Heterogeneity: $Tau^2 = 0.4$; $Chi^2 = 61.2$, df = 12 ($P < 0.01$); $I^2 = 80.4\%$			
**Chemo_IO**			
Cascone 2023	13	22	59.09 [36.35; 79.29]
Cascone 2023	14	22	63.64 [40.66; 82.80]
Cui 2022	3	30	10.00 [2.11; 26.53]
Forde 2022	70	179	39.11 [31.91; 46.67]
Hou 2022	1	11	9.09 [0.23; 41.28]
Provencio 2020_2022	11	46	23.91 [12.59; 38.77]
Rothschild 2021	16	67	23.88 [14.31; 35.86]
Shao 2023	13	29	44.83 [26.45; 64.31]
Shao 2023	12	31	38.71 [21.85; 57.81]
Shen 2021	5	37	13.51 [4.54; 28.77]
Shu 2020	9	30	30.00 [14.73; 49.40]
Sun 2022	4	20	20.00 [5.73; 43.66]
Tfayli 2020	8	15	53.33 [26.59; 78.73]
Yan 2022	10	37	27.03 [13.79; 44.12]
Yang 2018	8	24	33.33 [15.63; 55.32]
Zhang 2022	24	50	48.00 [33.66; 62.58]
Zhao 2021	3	33	9.09 [1.92; 24.33]
Zhu 2022	10	50	20.00 [10.03; 33.72]
Lei 2023	9	47	19.15 [9.15; 33.26]
Heymach 2023	124	366	33.88 [29.04; 38.98]
**Total (95% CI)**	**367**	**1146**	**30.34 [24.00; 37.52]**
Heterogeneity: $Tau^2 = 0.4$; $Chi^2 = 62.1$, df = 19 ($P < 0.01$); $I^2 = 69.4\%$			

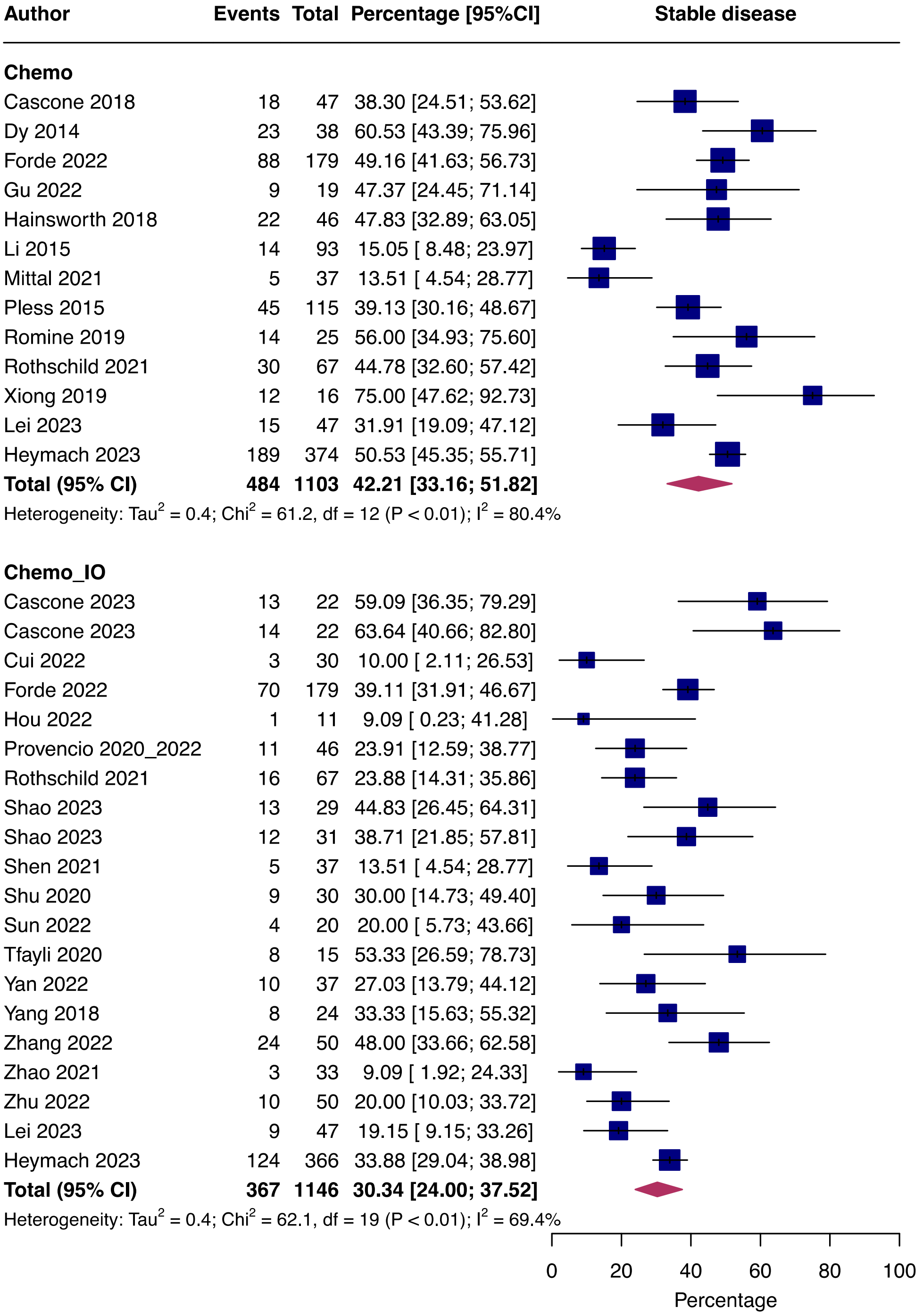

© 2024 Sorin M et al. *JAMA Oncology.*

eFigure 32. Pooled proportions across all studies comparing neoadjuvant chemoimmunotherapy to neoadjuvant chemotherapy by progressive disease per RECIST.

Author	Events	Total	Percentage [95%CI]
**Chemo**			
Cascone 2018	1	47	2.13 [0.05; 11.29]
Dy 2014	4	38	10.53 [2.94; 24.80]
Forde 2022	11	179	6.15 [3.11; 10.73]
Gu 2022	1	19	5.26 [0.13; 26.03]
Hainsworth 2018	6	46	13.04 [4.94; 26.26]
Li 2015	4	93	4.30 [1.18; 10.65]
Mittal 2021	4	37	10.81 [3.03; 25.42]
Pless 2015	16	115	13.91 [8.17; 21.61]
Romine 2019	1	25	4.00 [0.10; 20.35]
Rothschild 2021	3	67	4.48 [0.93; 12.53]
Xiong 2019	1	16	6.25 [0.16; 30.23]
Lei 2023	4	47	8.51 [2.37; 20.38]
Heymach 2023	15	374	4.01 [2.26; 6.53]
**Total (95% CI)**	**71**	**1103**	**7.14 [5.09; 9.94]**
Heterogeneity: $Tau^2 = 0.2$; $Chi^2 = 20.9$, df = 12 (P = 0.05); $I^2 = 42.6\%$			
**Chemo_IO**			
Cascone 2023	0	22	0.00 [0.00; 15.44]
Cascone 2023	1	22	4.55 [0.12; 22.84]
Cui 2022	0	30	0.00 [0.00; 11.57]
Forde 2022	8	179	4.47 [1.95; 8.62]
Provencio 2020_2022	0	46	0.00 [0.00; 7.71]
Rothschild 2021	4	67	5.97 [1.65; 14.59]
Shao 2023	0	29	0.00 [0.00; 11.94]
Shao 2023	1	31	3.23 [0.08; 16.70]
Shen 2021	0	37	0.00 [0.00; 9.49]
Shu 2020	2	30	6.67 [0.82; 22.07]
Sun 2022	2	20	10.00 [1.23; 31.70]
Tfayli 2020	3	15	20.00 [4.33; 48.09]
Yan 2022	1	37	2.70 [0.07; 14.16]
Yang 2018	2	24	8.33 [1.03; 27.00]
Zhang 2022	2	50	4.00 [0.49; 13.71]
Zhao 2021	1	33	3.03 [0.08; 15.76]
Zhu 2022	2	50	4.00 [0.49; 13.71]
Lei 2023	0	47	0.00 [0.00; 7.55]
Heymach 2023	11	366	3.01 [1.51; 5.31]
**Total (95% CI)**	**40**	**1135**	**4.46 [3.23; 6.13]**
Heterogeneity: $Tau^2 = < 0.1$; $Chi^2 = 16.2$, df = 18 (P = 0.58); $I^2 = 0\%$			

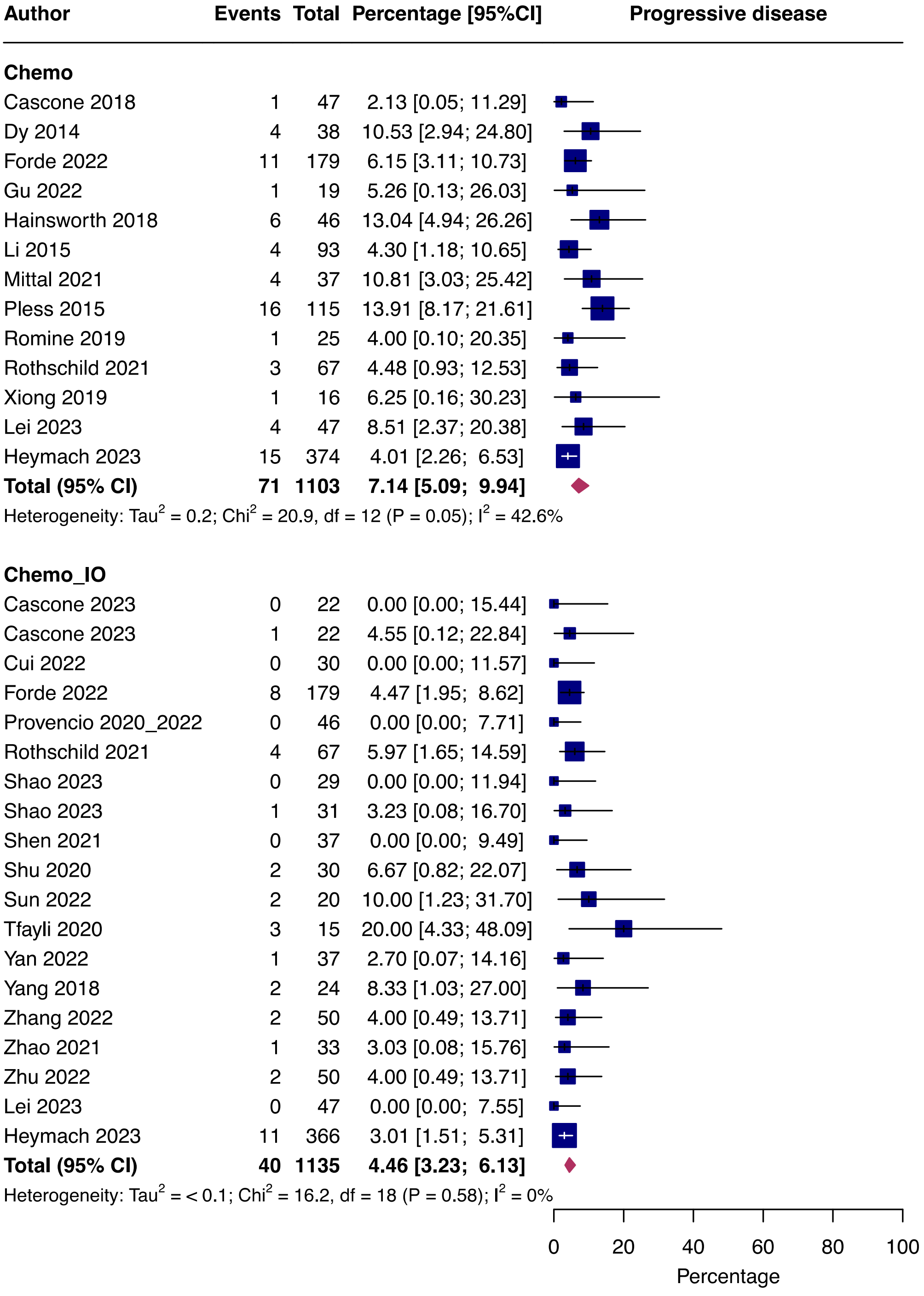

© 2024 Sorin M et al. *JAMA Oncology.*

eFigure 33. Pooled proportions across all studies comparing neoadjuvant chemoimmunotherapy to neoadjuvant chemotherapy by surgical resection.

Author	Events	Total	Percentage [95%CI]
**Chemo**			
Cascone 2018	37	47	78.72 [64.34; 89.30]
Chaft 2016	32	40	80.00 [64.35; 90.95]
Chen 2013	148	179	82.68 [76.33; 87.92]
Dy 2014	34	38	89.47 [75.20; 97.06]
Forde 2022	135	179	75.42 [68.44; 81.54]
Gu 2022	16	19	84.21 [60.42; 96.62]
Hainsworth 2018	27	46	58.70 [43.23; 73.00]
Li 2015	90	93	96.77 [90.86; 99.33]
Mittal 2021	17	37	45.95 [29.49; 63.08]
Pless 2015	94	115	81.74 [73.45; 88.33]
Provencio 2023	20	29	68.97 [49.17; 84.72]
Romine 2019	14	25	56.00 [34.93; 75.60]
Wakelee 2023	317	400	79.25 [74.94; 83.12]
Westeel 2013	257	267	96.25 [93.22; 98.19]
Westeel 2013	252	261	96.55 [93.56; 98.41]
Xiong 2019	8	16	50.00 [24.65; 75.35]
Zhu 2017	48	48	100.00 [92.60; 100.00]
Lei 2023	42	47	89.36 [76.90; 96.45]
Lu 2023	148	202	73.27 [66.60; 79.23]
Cascone 2023	178	232	76.72 [70.75; 82.00]
Heymach 2023	302	374	80.75 [76.38; 84.62]
Yue 2023	173	227	76.21 [70.13; 81.60]
**Total (95% CI)**	**2389**	**2921**	**81.18 [74.08; 86.69]**
Heterogeneity: Tau^2 = 0.8; Chi^2 = 150.5, df = 21 (P < 0.01); I^2 = 86.1%			
**Chemo_IO**			
Cascone 2023	22	22	100.00 [84.56; 100.00]
Cascone 2023	20	22	90.91 [70.84; 98.88]
Cui 2022	30	30	100.00 [88.43; 100.00]
Forde 2022	149	179	83.24 [76.95; 88.40]
Hou 2022	11	11	100.00 [71.51; 100.00]
Provencio 2020_2022	41	46	89.13 [76.43; 96.38]
Provencio 2023	53	57	92.98 [83.00; 98.05]
Rothschild 2021	55	67	82.09 [70.80; 90.39]
Shao 2023	26	29	89.66 [72.65; 97.81]
Shao 2023	29	31	93.55 [78.58; 99.21]
Shen 2021	37	37	100.00 [90.51; 100.00]
Shu 2020	26	30	86.67 [69.28; 96.24]
Sun 2022	16	20	80.00 [56.34; 94.27]
Tao 2023	48	55	87.27 [75.52; 94.73]
Tfayli 2020	11	15	73.33 [44.90; 92.21]
Wakelee 2023	325	397	81.86 [77.71; 85.53]
Yan 2022	34	37	91.89 [78.09; 98.30]
Yang 2018	13	24	54.17 [32.82; 74.45]
Zhang 2022	30	50	60.00 [45.18; 73.59]
Zhao 2021	30	33	90.91 [75.67; 98.08]
Zhu 2022	36	50	72.00 [57.51; 83.77]
Lei 2023	40	47	85.11 [71.69; 93.80]
Lu 2023	166	202	82.18 [76.19; 87.19]
Cascone 2023	178	229	77.73 [71.78; 82.95]
Heymach 2023	295	366	80.60 [76.17; 84.53]
Yue 2023	190	226	84.07 [78.64; 88.59]
**Total (95% CI)**	**1911**	**2312**	**83.28 [79.61; 86.41]**
Heterogeneity: Tau^2 = 0.2; Chi^2 = 60.7, df = 25 (P < 0.01); I^2 = 58.8%			

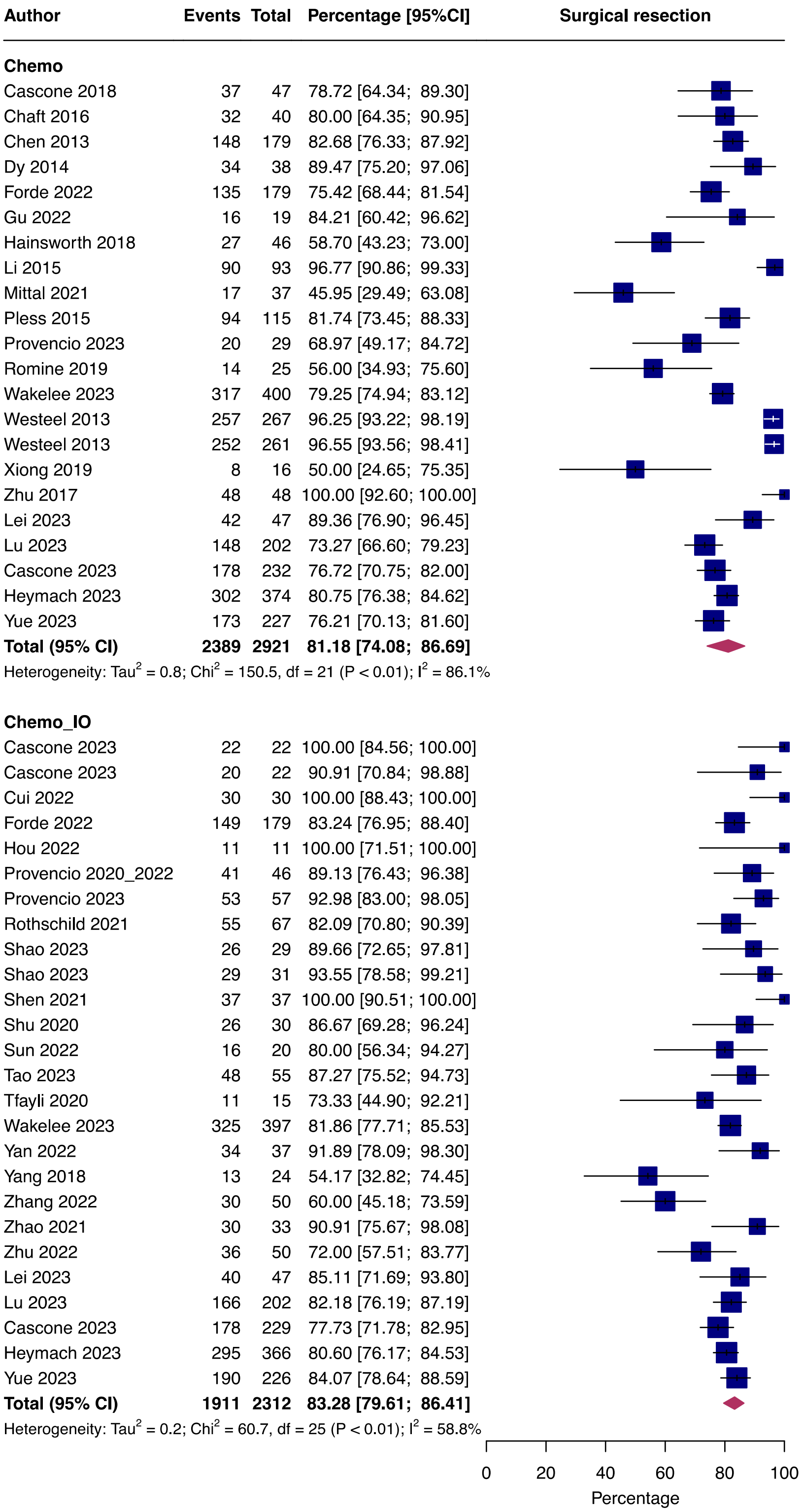

© 2024 Sorin M et al. *JAMA Oncology.*

eFigure 34. Pooled proportions across all studies comparing neoadjuvant chemoimmunotherapy to neoadjuvant chemotherapy by the number of patients who had R0 resection.

Author	Events	Total	Percentage [95%CI]
**Chemo**			
Cascone 2018	35	37	94.59 [81.81; 99.34]
Chaft 2016	29	32	90.62 [74.98; 98.02]
Dy 2014	32	34	94.12 [80.32; 99.28]
Forde 2022	105	135	77.78 [69.82; 84.48]
Gu 2022	15	16	93.75 [69.77; 99.84]
Hainsworth 2018	25	27	92.59 [75.71; 99.09]
Mittal 2021	17	17	100.00 [80.49; 100.00]
Pless 2015	76	94	80.85 [71.44; 88.24]
Provencio 2023	17	20	85.00 [62.11; 96.79]
Wakelee 2023	267	317	84.23 [79.74; 88.06]
Westeel 2013	244	257	94.94 [91.51; 97.28]
Westeel 2013	240	252	95.24 [91.83; 97.52]
Lei 2023	36	42	85.71 [71.46; 94.57]
Lu 2023	137	148	92.57 [87.09; 96.23]
Cascone 2023	161	178	90.45 [85.15; 94.34]
Heymach 2023	262	302	86.75 [82.40; 90.37]
**Total (95% CI)**	**1698**	**1908**	**89.65 [86.15; 92.34]**
Heterogeneity: $Tau^2 = 0.3$; $Chi^2 = 53.3$, df = 15 ($P < 0.01$); $I^2 = 71.9\%$			
**Chemo_IO**			
Cascone 2023	20	22	90.91 [70.84; 98.88]
Cascone 2023	19	20	95.00 [75.13; 99.87]
Forde 2022	124	149	83.22 [76.24; 88.84]
Hou 2022	11	11	100.00 [71.51; 100.00]
Provencio 2020_2022	41	41	100.00 [91.40; 100.00]
Provencio 2023	50	53	94.34 [84.34; 98.82]
Rothschild 2021	51	55	92.73 [82.41; 97.98]
Shao 2023	26	26	100.00 [86.77; 100.00]
Shao 2023	29	29	100.00 [88.06; 100.00]
Shen 2021	37	37	100.00 [90.51; 100.00]
Shu 2020	26	26	100.00 [86.77; 100.00]
Sun 2022	16	16	100.00 [79.41; 100.00]
Tao 2023	48	48	100.00 [92.60; 100.00]
Wakelee 2023	299	325	92.00 [88.50; 94.71]
Yan 2022	32	34	94.12 [80.32; 99.28]
Yang 2018	13	13	100.00 [75.29; 100.00]
Zhang 2022	30	30	100.00 [88.43; 100.00]
Zhao 2021	29	30	96.67 [82.78; 99.92]
Zhu 2022	36	36	100.00 [90.26; 100.00]
Lei 2023	37	40	92.50 [79.61; 98.43]
Lu 2023	159	166	95.78 [91.50; 98.29]
Cascone 2023	159	178	89.33 [83.83; 93.45]
Heymach 2023	269	295	91.19 [87.35; 94.16]
**Total (95% CI)**	**1561**	**1680**	**93.35 [90.94; 95.15]**
Heterogeneity: $Tau^2 = 0.2$; $Chi^2 = 34.5$, df = 22 ($P = 0.04$); $I^2 = 36.2\%$			

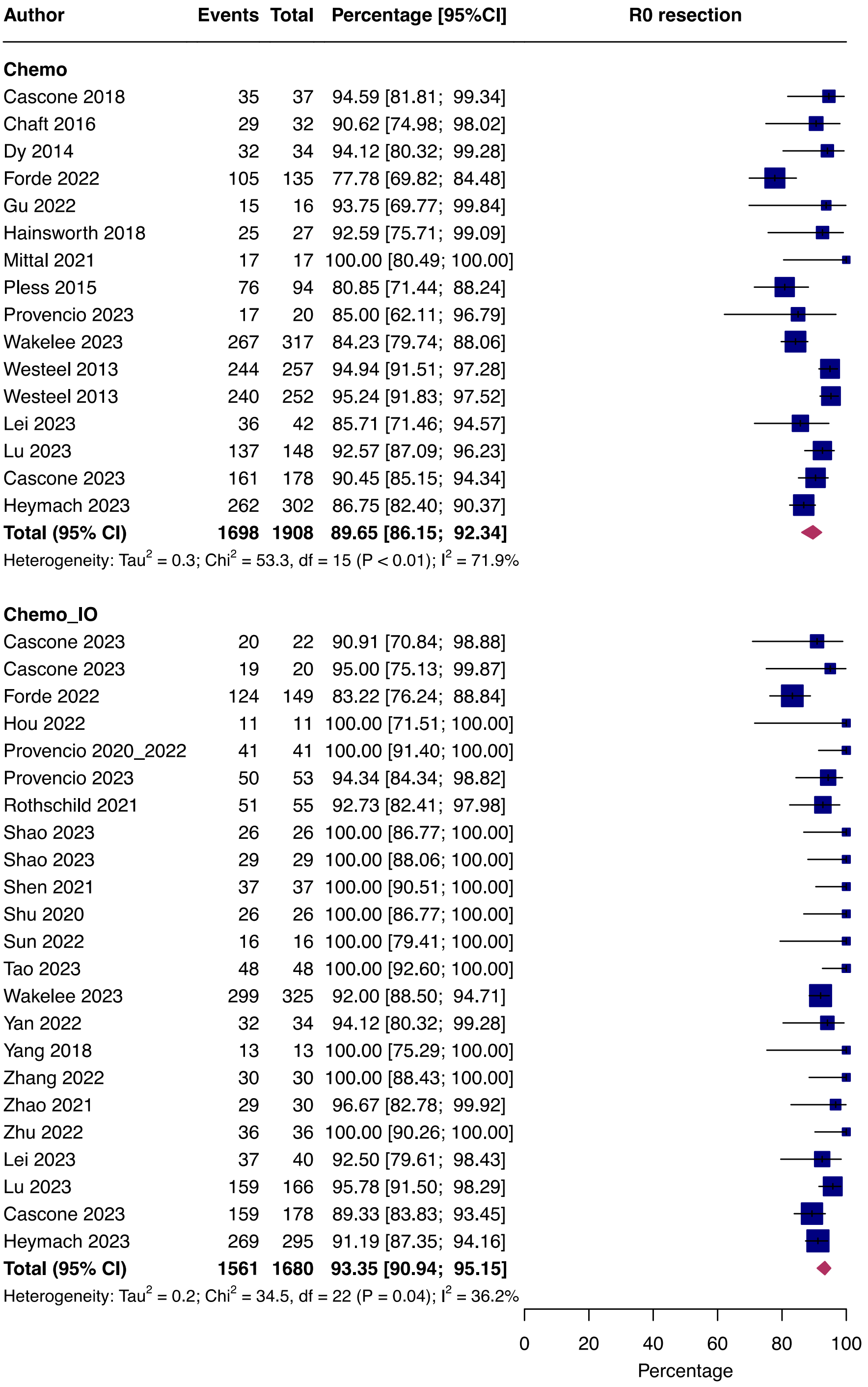

© 2024 Sorin M et al. *JAMA Oncology.*

eFigure 35. Pooled proportions across all studies comparing neoadjuvant chemoimmunotherapy to neoadjuvant chemotherapy by the number of patients who had a lobectomy.

Author	Events	Total	Percentage [95%CI]
**Chemo**			
Cascone 2018	31	37	83.78 [67.99; 93.81]
Chen 2013	68	148	45.95 [37.73; 54.32]
Dy 2014	26	34	76.47 [58.83; 89.25]
Forde 2022	82	135	60.74 [51.97; 69.03]
Hainsworth 2018	19	27	70.37 [49.82; 86.25]
Li 2015	39	90	43.33 [32.92; 54.20]
Mittal 2021	14	17	82.35 [56.57; 96.20]
Pless 2015	59	94	62.77 [52.18; 72.52]
Provencio 2023	17	20	85.00 [62.11; 96.79]
Wakelee 2023	238	317	75.08 [69.94; 79.74]
Lei 2023	34	42	80.95 [65.88; 91.40]
Lu 2023	123	148	83.11 [76.08; 88.76]
Cascone 2023	128	178	71.91 [64.70; 78.38]
Heymach 2023	221	302	73.18 [67.80; 78.09]
**Total (95% CI)**	**1099**	**1589**	**70.79 [63.23; 77.35]**
Heterogeneity: $Tau^2 = 0.3$; $Chi^2 = 97.4$, df = 13 ($P < 0.01$); $I^2 = 86.6\%$			
**Chemo_IO**			
Cascone 2023	17	22	77.27 [54.63; 92.18]
Cascone 2023	18	20	90.00 [68.30; 98.77]
Forde 2022	115	149	77.18 [69.60; 83.65]
Hou 2022	11	11	100.00 [71.51; 100.00]
Provencio 2020_2022	38	41	92.68 [80.08; 98.46]
Provencio 2023	41	53	77.36 [63.79; 87.72]
Rothschild 2021	43	55	78.18 [64.99; 88.19]
Shao 2023	20	26	76.92 [56.35; 91.03]
Shao 2023	23	29	79.31 [60.28; 92.01]
Shen 2021	22	37	59.46 [42.10; 75.25]
Shu 2020	19	26	73.08 [52.21; 88.43]
Sun 2022	13	16	81.25 [54.35; 95.95]
Wakelee 2023	256	325	78.77 [73.92; 83.09]
Yan 2022	24	34	70.59 [52.52; 84.90]
Yang 2018	10	13	76.92 [46.19; 94.96]
Zhang 2022	24	30	80.00 [61.43; 92.29]
Zhao 2021	22	30	73.33 [54.11; 87.72]
Zhu 2022	35	36	97.22 [85.47; 99.93]
Lei 2023	36	40	90.00 [76.34; 97.21]
Lu 2023	134	166	80.72 [73.89; 86.43]
Cascone 2023	142	178	79.78 [73.12; 85.41]
Heymach 2023	238	295	80.68 [75.70; 85.02]
**Total (95% CI)**	**1301**	**1632**	**78.97 [76.88; 80.92]**
Heterogeneity: $Tau^2 = < 0.1$; $Chi^2 = 26.6$, df = 21 ($P = 0.19$); $I^2 = 20.9\%$			

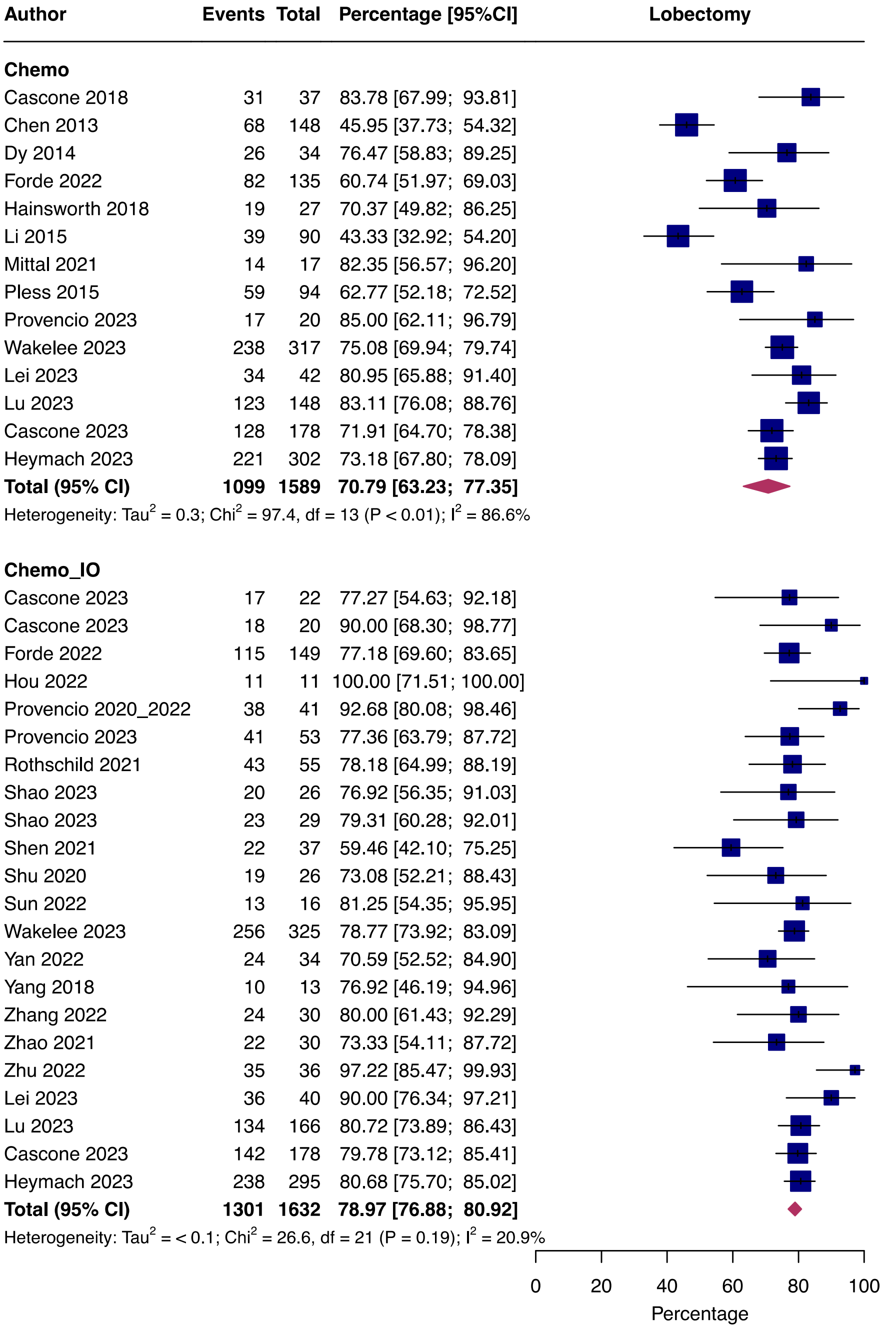

© 2024 Sorin M et al. *JAMA Oncology.*

eFigure 36. Pooled proportions across all studies comparing neoadjuvant chemoimmunotherapy to neoadjuvant chemotherapy by the number of patients who had a pneumonectomy.

Author	Events	Total	Percentage [95%CI]
**Chemo**			
Cascone 2018	4	37	10.81 [3.03; 25.42]
Chen 2013	75	148	50.68 [42.34; 58.98]
Dy 2014	6	34	17.65 [6.76; 34.53]
Forde 2022	34	135	25.19 [18.11; 33.38]
Hainsworth 2018	3	27	11.11 [2.35; 29.16]
Li 2015	32	90	35.56 [25.74; 46.35]
Mittal 2021	3	17	17.65 [3.80; 43.43]
Pless 2015	19	94	20.21 [12.63; 29.75]
Provencio 2023	2	20	10.00 [1.23; 31.70]
Wakelee 2023	39	317	12.30 [8.90; 16.43]
Lei 2023	8	42	19.05 [8.60; 34.12]
Lu 2023	14	148	9.46 [5.27; 15.36]
Cascone 2023	24	178	13.48 [8.83; 19.39]
Heymach 2023	29	302	9.60 [6.53; 13.50]
**Total (95% CI)**	**292**	**1589**	**17.76 [12.68; 24.32]**
Heterogeneity: $Tau^2 = 0.4$; $Chi^2 = 138.4$, df = 13 ($P < 0.01$); $I^2 = 90.6\%$			
**Chemo_IO**			
Cascone 2023	2	22	9.09 [1.12; 29.16]
Cascone 2023	1	20	5.00 [0.13; 24.87]
Forde 2022	25	149	16.78 [11.16; 23.76]
Hou 2022	0	11	0.00 [0.00; 28.49]
Provencio 2020_2022	3	41	7.32 [1.54; 19.92]
Provencio 2023	6	53	11.32 [4.27; 23.03]
Rothschild 2021	5	55	9.09 [3.02; 19.95]
Shao 2023	0	26	0.00 [0.00; 13.23]
Shao 2023	2	29	6.90 [0.85; 22.77]
Shen 2021	2	37	5.41 [0.66; 18.19]
Shu 2020	3	26	11.54 [2.45; 30.15]
Sun 2022	3	16	18.75 [4.05; 45.65]
Wakelee 2023	37	325	11.38 [8.14; 15.35]
Yan 2022	2	34	5.88 [0.72; 19.68]
Yang 2018	1	13	7.69 [0.19; 36.03]
Zhang 2022	4	30	13.33 [3.76; 30.72]
Zhao 2021	6	30	20.00 [7.71; 38.57]
Zhu 2022	0	36	0.00 [0.00; 9.74]
Lei 2023	4	40	10.00 [2.79; 23.66]
Lu 2023	15	166	9.04 [5.15; 14.47]
Cascone 2023	16	178	8.99 [5.23; 14.19]
Heymach 2023	27	295	9.15 [6.12; 13.04]
**Total (95% CI)**	**164**	**1632**	**10.58 [8.94; 12.49]**
Heterogeneity: $Tau^2 = < 0.1$; $Chi^2 = 19.7$, df = 21 ($P = 0.54$); $I^2 = 0\%$			

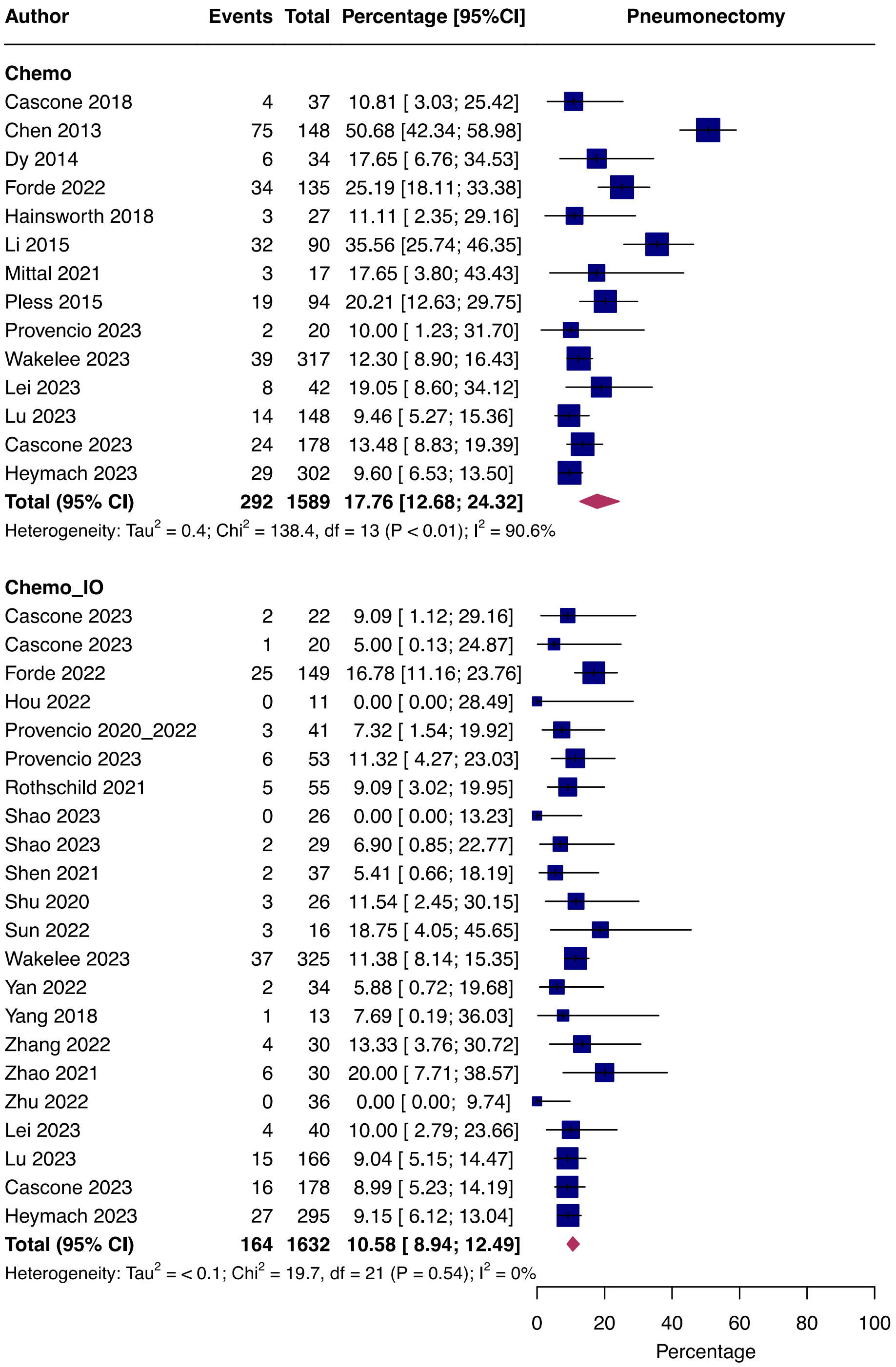

© 2024 Sorin M et al. *JAMA Oncology.*

eFigure 37. Pooled proportions across all studies comparing neoadjuvant chemoimmunotherapy to neoadjuvant chemotherapy by the incidence of death events.

Author	Events	Months	Total	Events per 100 person−months [95% CI]
**Chemo**				
Cascone 2018	29	62.00	47	0.47 [0.33; 0.67]
Chen 2013	128	53.80	179	2.38 [2.00; 2.83]
Dy 2014	15	37.10	38	0.40 [0.24; 0.67]
Li 2015	33	60.00	93	0.55 [0.39; 0.77]
Pless 2015	64	52.00	115	1.23 [0.96; 1.57]
Provencio 2023	11	26.10	29	0.42 [0.23; 0.76]
Romine 2019	8	95.00	25	0.08 [0.04; 0.17]
Rothschild 2021	15	28.60	67	0.52 [0.32; 0.87]
**Total (95% CI)**	.		**593**	**0.54 [0.28; 1.04]**
Heterogeneity: $Tau^2 = 0.8$; $Chi^2 = 200.3$, df = 7 ($P < 0.01$); $I^2 = 96.5\%$				
**Chemo_IO**				
Provencio 2023	12	26.10	57	0.46 [0.26; 0.81]
Rothschild 2021	15	28.60	67	0.52 [0.32; 0.87]
Shen 2021	0	7.00	37	0.00 [0.00; 1.14]
Shu 2020	4	12.90	30	0.31 [0.12; 0.83]
Sun 2022	2	27.00	20	0.07 [0.02; 0.30]
Yan 2022	1	12.10	37	0.08 [0.01; 0.59]
Zhang 2022	6	13.60	50	0.44 [0.20; 0.98]
Zhao 2021	1	10.30	33	0.10 [0.01; 0.69]
Zhu 2022	14	22.00	50	0.64 [0.38; 1.07]
**Total (95% CI)**	.		**381**	**0.34 [0.22; 0.54]**
Heterogeneity: $Tau^2 = 0.2$; $Chi^2 = 16$, df = 8 ($P = 0.04$); $I^2 = 49.9\%$				
Test for subgroup differences: $Chi^2 = 1.2$, df = 1 ($P = 0.27$)				

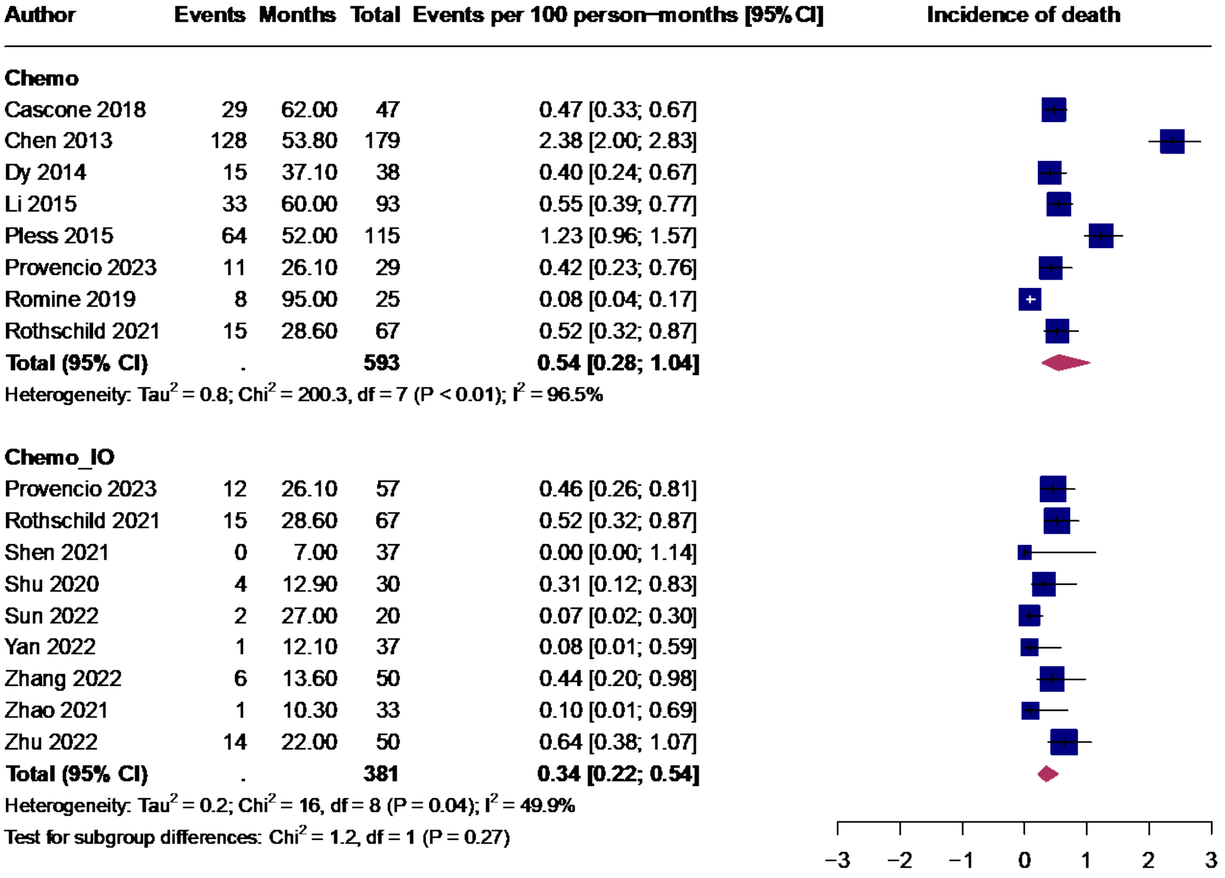

© 2024 Sorin M et al. *JAMA Oncology.*

eTable 1. Search strategy for Embase.

#	Query	Results
1	(random* or factorial* or placebo* or assign* or allocat* or crossover*).tw.	2572740
2	(cross adj over*).tw.	38625
3	(trial* and (control* or comparative)).tw.	842069
4	((blind* or mask*) and (single or double or triple or treble)).tw.	329610
5	(treatment adj arm*).tw.	26745
6	(control* adj group*).tw.	852318
7	((phase adj (III or three)) or (phase adj (II or two))).tw.	175257
8	(versus or vs).tw.	2788454
9	rct.tw.	54724
10	Crossover Procedure/	75990
11	double blind procedure/	214267
12	single blind procedure/	52165
13	randomization/	99085
14	placebo/	414590
15	exp clinical trial/	1869347
16	parallel design/	18809
17	latin square design/	491
18	randomized controlled trial/	791270
19	"randomized controlled trial (topic)"/	264085
20	controlled clinical trial/	471606
21	"controlled clinical trial (topic)"/	13456
22	or/1-21	6567900
23	exp animal/ or exp nonhuman/ or exp animal experiment/ or exp animal model/	35123223
24	exp human/	27044216
25	23 not 24	8079007
26	22 not 25	5777199
27	(NSCLC or ((cancer or carcinoma or tumo?r* or neoplasm*) adj2 (non-small cell or nonsmall cell or [illegible])).tw,kf. or lung tumor/	415333
28	lung non small cell cancer/ or non small cell lung cancer/	163890
29	27 or 28	439985
30	(neoadjuvant or neo-adjuvant or preoperative or pre-operative or induction).tw,kf.	1335880
31	exp neoadjuvant therapy/	52738
32	exp induction chemotherapy/	18384
33	30 or 31 or 32	1350877
34	(immunotherap* or chemoimmunotherap* or chemo-immunotherap*).tw,kf.	229384
35	immune checkpoint inhibitor*.tw,kf.	38043
36	exp immunological antineoplastic agent/ or immunological antineoplastic agent.tw,kf. or immune checkpoint [illegible]or/	387482
37	(nivolumab or Opdivo).tw,kf,dy. or nivolumab/	39743
38	(bms 936558 or bms936558 or cmab 819 or cmab819 or mdx 1106 or mdx1106 or ono 4538 or ono4538 or [illegible]).tw,kf,dy.	1673
39	(pembrolizumab or Keytruda).tw,kf,dy. or pembrolizumab/	39062
40	(mk 3475 or mk4375 or sch 900475 or sch900475).tw,kf,dy.	733
41	cemiplimab.tw,kf,dy. or cemiplimab/	1959
42	(Libtayo or regn 2810 or regn2810 or sar 439684 or sar439684).tw,kf,dy.	225

© 2024 Sorin M et al. *JAMA Oncology.*

43	durvalumab.tw,kf,dy. or durvalumab/	10884
44	(Imfinzi or medi 4736 or medi4736).tw,kf,du.	967
45	atezolizumab.tw,kf,du. or atezolizumab/	16156
46	(monoclonal antibody mpdl 3280a or monoclonal antibody mpdl3280a or mpdl 3280a or mpdl3280a or rg 7446 446 or ro 5541267 or ro5541267 or tecentriq or tecntriq).tw,kf,du.	1057
47	ipilimumab.tw,kf,du. or ipilimumab/	25821
48	(bms 734016 or bms734016 or cs 1002 or cs1002 or ibi 310 or ibi310 or "mdx 010" or mdx 101 or mdx010 or 01 or strentarga or yervoy).tw,kf,du.	1341
49	or/34-48	567967
50	chemotherap*.tw,kf.	822593
51	antineoplastic agent/ or antineoplastic agent.tw,kf.	372991
52	gemcitabine/ or (gemcitabine or Gemzar).tw,kf,dy.	76154
53	cisplatin/ or (cisplatin or platinol).tw,kf,dy.	231101
54	docetaxel/ or (docetaxel or Taxotere).tw,kf,dy.	76416
55	paclitaxel/ or (paclitaxel or Abraxane).tw,kf,dy.	140502
56	carboplatin/ or (carboplatin or Paraplatin).tw,kf,dy.	90486
57	or/50-56	1263609
58	26 and 29 and 33 and ((49 and 57) or 57)	4884
59	58 not limit 58 to conference abstracts	3025
60	limit 59 to dd=20130101-20231025	118

© 2024 Sorin M et al. *JAMA Oncology.*

eTable 2. Search strategy for MEDLINE.

#	Query	Results
1	Carcinoma, Non-Small-Cell Lung/	71701
2	(NSCLC or ((cancer or carcinoma or tumo?r* or neoplasm*) adj2 (non-small cell or nonsmall cell or lung))).tw,kf.	243394
3	Lung Neoplasms/	260560
4	limit 3 to yr="1966 - 1986"	34104
5	1 or 2 or 4	273228
6	(neoadjuvant or neo-adjuvant or preoperative or pre-operative or induction).tw,kf.	944661
7	Neoadjuvant Therapy/	29030
8	exp Induction Chemotherapy/	3872
9	6 or 7 or 8	950079
10	(immunotherap* or chemoimmunotherap* or chemo-immunotherap*).tw,kf.	149010
11	immune checkpoint inhibitor*.tw,kf.	24383
12	(31yo63lbsn or bms 936558 or bms-936558 or bms936558 or mdx 1106 or mdx-1106 or mdx1106 or nivolumab or ono 4538 or ono-4538 or ono4538 or opdivo).tw,kf,nm. or Nivolumab/	9930
13	(pembrolizumab or Keytruda).tw,kf,nm. or pembrolizumab/	9254
14	exp Antineoplastic Agents, Immunological/ or antineoplastic agent immunological.tw,kf,nm.	73950
15	(cemiplimab or Libtayo).tw,kf,nm. or cemiplimab/	391
16	(durvalumab or Imfinzi).tw,kf,nm. or durvalumab/	1603
17	(atezolizumab or Tecentriq).tw,kf,nm. or atezolizumab/	3252
18	(6t8c155666 or anti ctla 4 mab ipilimumab or anti-ctla-4 mab ipilimumab or ipilimumab or ipilimumab, anti-ctla-4 mab or "mdx 010" or mdx ctla 4 or mdx-010 or mdx-ctla-4 or mdx010 or yervoy).tw,kf,nm. or Ipilimumab/	5663
19	or/10-18	222788
20	chemotherap*.tw,kf.	506937
21	gemcitabine/ or (gemcitabine or Gemzar).tw,kf,nm.	21128
22	exp Antineoplastic Agents/ or antineoplastic agents.tw,kf.	1254372
23	(cisplatin or Platinol).tw,kf,nm. or Cisplatin/	88362
24	docetaxel.tw,kf,nm. or Docetaxel/	20133
25	(15h5577cqd or 699121phca or docetaxel or docetaxel anhydrous or docetaxel hydrate or docetaxel trihydrate or docetaxol or n dibenzoyl n tert butoxycarbonyl 10 deacetyltaxol or n-debenzoyl-n-tert-butoxycarbonyl-10-deacetyltaxol or nsc 628503 or rp 56976 or rp-56976 or rp56976 or taxoltere metro or Taxotere).tw,kf,nm.	20321
26	Paclitaxel/ or (paclitaxel or Abraxane).tw,kf,nm.	45232
27	(carboplatin or Paraplatin).tw,kf,nm. or carboplatin/	20660
28	or/20-27	1563671
29	(Randomized Controlled Trial or Controlled Clinical Trial or Pragmatic Clinical Trial or Equivalence Trial or Clinical Trial, Phase II or Clinical Trial, Phase III).pt.	722140
30	Randomized Controlled Trial/	601405
31	exp Randomized Controlled Trials as Topic/	168460
32	Controlled Clinical Trial/	95423
33	exp Controlled Clinical Trials as Topic/	174172
34	Randomization/	107032
35	Random Allocation/	107032
36	Double-Blind Studies/	176369
37	Single-Blind Studies/	32983
38	Placebos/	35932

39	Control Group/	2044
40	(random* or sham or placebo*).ti,ab,hw,kf.	1846289
41	((singl* or doubl*) adj (blind* or dumm* or mask*)).ti,ab,hw,kf.	270225
42	((tripl* or trebl*) adj (blind* or dumm* or mask*)).ti,ab,hw,kf.	1687
43	(control* adj3 (study or studies or trial* or group*)).ti,ab,kf.	1250932
44	(Nonrandom* or non random* or non-random* or quasi-random* or quasirandom*).ti,ab,hw,kf.	55816
45	allocated.ti,ab,hw.	85621
46	((open label or open-label) adj5 (study or studies or trial*)).ti,ab,hw,kf.	45829
47	((equivalence or superiority or non-inferiority or noninferiority) adj3 (study or studies or trial*)).ti,ab,hw,kf.	12543
48	(pragmatic study or pragmatic studies).ti,ab,hw,kf.	618
49	((pragmatic or practical) adj3 trial*).ti,ab,hw,kf.	7924
50	((quasiexperimental or quasi-experimental) adj3 (study or studies or trial*)).ti,ab,hw,kf.	12752
51	(phase adj3 (II or III or "2" or "3") adj3 (study or studies or trial*)).ti,hw,kf.	86301
52	or/29-51	2674296
53	5 and 9 and ((19 and 28) or 28) and 52	1496
54	limit 53 to dt=20130101-20231025	524

© 2024 Sorin M et al. *JAMA Oncology.*

eTable 3. Non-randomized study characteristics.

Treatment mode	First author	Publication year	NCT number	Clinical trial	Study phase	Study design	Neoadjuvent treatment regimen	Main inclusion criteria	Sample size	Proportion of males (%)	Median age, years	Proportion of SCC (%)	Proportion of stage III (%)
Chemotherapy	Chen	2013	NA	NA	NA	Single-arm	Mitomycin + cisplatin + vindesine	Stage I-IIIA resected NSCLC	179	74	61	44	37
Chemotherapy	Westeel	2013	NCT00198354	IFCT 0002	III	Single-arm	Preoperative chemotherapy	Stage IA-II resectable NSCLC	267	81	Pre: 60	Pre: 43	Pre: 0
Chemotherapy	Westeel	2013	NCT00198354	IFCT 0002	III	Single-arm	Perioperative chemotherapy	Stage IA-II resectable NSCLC	261	81	Peri: 62	Peri: 44	Peri: 0
Chemotherapy	Dy	2014	NA	NA	II	Single-arm	Cisplatin + pemetrexed	Stage IB-IIIA resectable NSCLC	38	50	62.5	26	50
Chemotherapy	Li	2015	NA	NA	NA	Single-arm	Docetaxel + cisplatin	NSCLC	93	77	NR	53	100
Chemotherapy	Pless	2015	NCT00030771	NA	III	Single-arm	Cisplatin + docetaxel	Stage IIIA N2 resectable NSCLC	115	67	59	31	100
Chemotherapy	Chaft	2016	NA	NEOSCAN	II	Single-arm	Platinum-doublet chemotherapy	Stage IB-IIIA resectable lung cancer	40	35	63	18	80
Chemotherapy	Zhao	2016	NCT02497118	NA	NA	Single-arm	Cisplatin + vinorelbine	Stage IIIA N2 resectable NSCLC	10	90	58	30	100
Chemotherapy	Zhu	2017	NA	NA	NA	Single-arm	Gemcitabine hydrochloride and cisplatin	Stage I-II NSCLC	48	54	NA	NA	0
Chemotherapy	Chen	2018	NA	NA	NA	Single-arm	Pemetrexed + cisplatin	EGFR mutant lung adenocarcinoma	43	28	NA	0	100
Chemotherapy	Hainsworth	2018	NA	NA	II	Single-arm	Pemetrexed + carboplatin	Resectable non-squamous NSCLC	46	39	65	0	52
Chemotherapy	Cascone	2018	NCT00254384	NA	II	Single-arm	Cisplatin + docetaxel	Stage I-III resectable NSCLC	47	62	64	38	40
Chemoimmunotherapy	Yang	2018	NCT01820754	TOP1201	II	Single-arm	Ipilimumab + paclitaxel + cisplatin or ipilimumab + paclitaxel + carboplatin	Stage IB-IIIA NSCLC	24	50	65	38	79
Chemotherapy	Ma	2019	NA	NA	NA	Single-arm	Docetaxel + cisplatin	Stage IIIA NSCLC	41	NA	NA	NA	100
Chemotherapy	Romine	2019	NCT00227539	NA	II	Single-arm	Cisplatin + pemetrexed	Stage IB-IIIB resectable NSCLC	25	48	62	12	76
Chemotherapy	Xiong	2019	NCT01217619	NA	NA	Single-arm	Cisplatin doublet chemotherapy + vinorelbine or gemcitabine or paclitaxel or docetaxel or permetrexed	Stage IIIA N2 NSCLC	16	63	60	0	100
Chemoimmunotherapy	Shu	2020	NCT02716038	NA	II	Single-arm	Atezolizumab + nab-paclitaxel + carboplatin	Stage IB-IIIA resectable NSLC	30	50	67	40	77
Chemoimmunotherapy	Tfayli	2020	NCT03480230	NA	NA	Single-arm	Avelumab + chemotherapy	Stage IB-IIIA resectable NSCLC	15	47	65	13	53
Chemoimmunotherapy	Shen	2021	NA	NA	NA	Single-arm	Pembrolizumab + albumin-paclitaxel + carboplatin	Stage IIB to IIIB resectable squamous NSCLC	37	95	63	100	NA
Chemotherapy	Zhu	2021	NA	NA	II	Single-arm	Nab-paclitaxel + carboplatin	Stage IIIA N2 resectable squamous NSCLC	36	100	58	100	100
Chemotherapy	Mittal	2021	NA	NA	II	Single-arm	Paclitaxel and carboplatin	Stage IB-IIIB NSCLC	37	57	55	35	81

© 2024 Sorin M et al. *JAMA Oncology.*

Chemoimmunotherapy	Rothschild	2021	NCT02572843	SAKK 16/14	II	Single-arm	Durvalumab + cisplatin + docetaxel	Stage IIIA N2 NSCLC	67	52	61	33	100
Chemoimmunotherapy	Zhao	2021	NCT04304248	NA	II	Single-arm	Toripalimab + carboplatin + pemetrexed/nab-paclitaxel	Stage IIIA-IIIB resectable NSCLC	33	82	61	55	100
Chemoimmunotherapy	Zhang	2022	ChiCTR1900023758	NA	II	Single-arm	Sintilimab + carboplatin + pemetrexed or sintilimab + carboplatin + gemcitabine	Stage IB-IIIA resectable NSCLC	50	88	NA	56	100
Chemoimmunotherapy	Zhu	2022	ChiCTR190002401	LungMate 002	II	Single-arm	Toripalimab + chemotherapy	Stage II-IIIB resectable NSCLC	50	84	66	64	92
Chemoimmunotherapy	Cui	2022	NA	NA	II	Single-arm	Toripalimab + nabpaclitaxel or toripalimab + pemetrexed & carboplatin	Stage IIIA or T3-4N2 IIIB resectable NSCLC	30	80	NA	50	100
Chemoimmunotherapy	Hou	2022	NA	NA	I	Single-arm	Toripalimab + platinum-paclitaxel	Stage II-IIIB resectable NSCLC	11	91	63	82	64
Chemotherapy	Gu	2022	NCT04197076	NA	NA	Single-arm	Chemotherapy	Stage IIB-IIIB resectable NSCLC	19	84	64	63	89
Chemoimmunotherapy	Yan	2022	NCT04316364	NA	IB	Single-arm	Adebrelimab + nab-paclitaxel + carboplatin	Stage II-IIIB resectable NSCLC	37	95	63	84	70
Chemoimmunotherapy	Sun	2022	NCT04326153	NA	II	Single-arm	Sintilimab + nab-paclitaxel + carboplatin	Stage IIIA-IIIB resectable NSCLC	20	90	59.5	80	100
Chemoimmunotherapy	Cascone	2023	NCT03158129	NEOSTAR	II	Single-arm	Nivolumab + cisplatin & docetaxel or pemetrexed	Stage IB-IIIA NSCLC	22	45	69.5	23	50
Chemoimmunotherapy	Cascone	2023	NCT03158129	NEOSTAR	II	Single-arm	Ipilimumab + nivolumab + cisplatin & docetaxel or pemetrexed	Stage IB-IIIA resectable NSCLC	22	68	63.1	23	59
Chemoimmunotherapy	Li	2023	NCT04422392	NA	II	Single-arm	Sintilimab + platinum-doublet chemotherapy	Stage IIIA-N2 resectable NSCLC	39	97	62-64	67	100
Chemoimmunotherapy	Shao	2023	NCT04459611	neoSCORE	II	Single-arm	Sintilimab + chemotherapy (2 cycles)	Stage IB-IIIA NSCLC	29	86	66	66	62
Chemoimmunotherapy	Shao	2023	NCT04459611	neoSCORE	II	Single-arm	Sintilimab + chemotherapy (3 cycles)	Stage IB-IIIA NSCLC	31	74	63	55	45
Chemoimmunotherapy	Tao	2023	NCT04606303	Renaissance	II	Single-arm	Toripalimab + platinum-doublet chemotherapy	Stage IIB-IIIB NSCLC	55	91	62	80	71
Chemotherapy	Zhong	2019_2023	NCT01407822	EMERGING-CTONG 1103	II	Single-arm	Gemcitabine + cisplatin	EGFR mutant stage IIIA-N2 resectable NSCLC	35	23	58	NA	100
Chemoimmunotherapy	Provencio	2020_2022	NCT03081689	NADIM	II	Single-arm	Nivolumab + paclitaxel + carboplatin	Stage IIIA resectable NSCLC	46	74	63	35	100

© 2024 Sorin M et al. *JAMA Oncology.*

eTable 4. Johanna Brigs bias assessment.

Author (Year)	1. Were the two/three groups similar and recruited from the same population?	2. Were the exposures measured similarly to assign people to both exposed and unexposed groups?	3. Was the exposure measured in a valid and reliable way?	4. Were confounding factors identified?	5. Were strategies to deal with confounding factors stated?	6. Were the groups/participants free of the outcome at the start of the study?	7. Were the outcomes measured in a valid and reliable way?	8. Was the follow up time reported and sufficient to be long enough for outcomes to occur?	9. Was follow up complete, and if not, were the reasons to loss to follow up described and explored?	10. Were strategies to address incomplete follow up utilized?	11. Was appropriate statistical analysis used?
Cascone (2018)	N/A	N/A	N/A	N	N/A	Y	Y	Y	N	U	Y
Cascone (2023)	N/A	N/A	N/A	N	N/A	Y	Y	Y	N	U	Y
Chaft (2016)	N/A	N/A	N/A	N	N/A	Y	Y	Y	N	U	Y
Chen (2013)	N/A	N/A	N/A	U	Y	Y	Y	Y	Y	N/A	Y
Chen (2018)	N/A	N/A	N/A	N	N/A	Y	Y	Y	Y	N/A	Y
Cui (2022)	N/A	N/A	N/A	N	N/A	Y	Y	N/A	N/A	N/A	Y
Dy (2014)	N/A	N/A	N/A	N	N/A	Y	Y	Y	N	U	Y
Gu (2022)	N/A	N/A	N/A	U	U	Y	Y	Y	N	U	Y
Hainsworth (2018)	N/A	N/A	N/A	N	N/A	Y	Y	Y	N	U	Y
Hou (2022)	N/A	N/A	N/A	U	U	Y	Y	Y	Y	N/A	Y
Li (2015)	N/A	N/A	N/A	N	N/A	Y	Y	Y	Y	N/A	Y
Li (2023)	N/A	N/A	N/A	U	U	Y	Y	U	N	U	Y
Ma (2019)	N/A	N/A	N/A	N	N/A	Y	Y	U	U	U	Y
Mittal (2021)	N/A	N/A	N/A	N	N/A	Y	Y	Y	N	U	Y
Pless (2015)	N/A	N/A	N/A	N	N/A	Y	Y	Y	N	U	Y
Provencio (2020)	N/A	N/A	N/A	N	N/A	Y	Y	Y	N	U	Y
Provencio (2022)	N/A	N/A	N/A	N	N/A	Y	Y	Y	N	U	Y
Romine (2019)	N/A	N/A	N/A	N	N/A	Y	Y	Y	Y	N/A	Y
Rothschild (2021)	N/A	N/A	N/A	N	N/A	Y	Y	Y	N	U	Y
Shao (2023)	N/A	N/A	N/A	N	N/A	Y	Y	Y	N	U	Y
Shen (2021)	N/A	N/A	N/A	U	U	Y	Y	N	U	U	Y
Shu (2020)	N/A	N/A	N/A	N	N/A	Y	Y	Y	N	U	Y
Sun (2022)	N/A	N/A	N/A	U	U	Y	Y	N	N	N	Y
Tao (2023)	N/A	N/A	N/A	N	N/A	Y	Y	N	U	Y	
Tfayli (2020)	N/A	N/A	N/A	N	N/A	Y	Y	U	U	U	Y
Westeel (2013)	N/A	N/A	N/A	N	N/A	Y	Y	Y	Y	N/A	Y
Xiong (2019)	N/A	N/A	N/A	N	N/A	Y	Y	U	N	U	Y
Yang (2018)	N/A	N/A	N/A	N	N/A	Y	Y	U	U	U	Y
Yan (2022)	N/A	N/A	N/A	U	U	Y	Y	Y	N	U	Y

**Zhang (2022)**	N/A	N/A	N/A	U	U	Y	Y	Y	N	U	Y
**Zhao (2016)**	N/A	N/A	N/A	U	U	Y	Y	U	N	U	Y
**Zhao (2021)**	N/A	N/A	N/A	N	N/A	Y	Y	N	U	U	Y
**Zhong (2019)**	N/A	N/A	N/A	N	N/A	Y	Y	Y	N	U	Y
**Zhong (2023)**	N/A	N/A	N/A	N	N/A	Y	Y	Y	N	U	Y
**Zhu (2017)**	N/A	N/A	N/A	N	N/A	Y	U	Y	U	U	Y
**Zhu (2021)**	N/A	N/A	N/A	U	U	Y	Y	Y	Y	N/A	Y
**Zhu (2022)**	N/A	N/A	N/A	N	N/A	Y	Y	N	N	U	Y

© 2024 Sorin M et al. *JAMA Oncology.*

eTable 5. Surgical and efficacy outcomes for RCTs.

First author	ITT Chemo IO	ITT Chemo	Surgical resection Chemo IO	Surgical resection Chemo	R0 resection Chemo IO	R0 resection Chemo	MPR Chemo IO	MPR Chemo	pCR Chemo IO	pCR Chemo	Median follow up time (months)	PFS/EFS HR	OS HR
Forde 2022	179	179	149	135	124	105	66	16	43	4	41.4	0.68 (95% 0.49-0.93)	0.57 (95% CI 0.38-0.87)
Wakelee 2023	397	400	325	317	299	267	120	44	72	16	36.6	0.59 (95% 0.48-0.72)	0.72 (95% CI 0.56-0.93)
Heymach 2023	366	374	295	302	269	262	122	46	63	16	11.7	0.68 (95% CI 0.53-0.88)	NA
Provencio 2023	57	29	53	20	50	17	30	4	21	2	26.1	0.47 (95% CI 0.25-0.88)	0.43 (95% CI 0.19-0.98)
Lu 2023	202	202	166	148	159	137	98	17	50	2	18.25	0.4 (95% CI 0.27-0.57)	0.62 (95% CI 0.38-0.999)
Cascone 2023	229	232	178	178	159	161	81	28	58	11	25.4	0.59 (95% CI 0.44-0.79)	NA
Lei 2023	47	47	40	42	37	36	28	7	14	4	14.1	0.52 (95% CI 0.21-1.29)	NA
Yue 2023	226	227	190	173	NA	NA	127	34	92	13	16.8	NA	NA

© 2024 Sorin M et al. *JAMA Oncology.*

eTable 6. Definition of EFS, PFS and criteria used for pathological endpoints. Abbreviations: EFS, event-free survival; PFS, progression-free survival; irPRC, immune-related pathologic response criteria; IASLC, International Association for the Study of Lung Cancer.

First author	EFS or PFS definition	Pathological endpoints criteria
Forde 2022	Event-free survival was defined as the time from randomization to any progression of disease precluding surgery, progression or recurrence of disease after surgery, progression of disease in the absence of surgery, or death from any cause; data on patients with subsequent therapy were censored at the last tumor assessment that could be evaluated on or before the date of subsequent therapy. EFS was based on BICR assessment per RECIST v1.1	irPRC
Wakelee 2023	Event-free survival was defined the time from randomization to the first occurrence of local progression that precluded the planned surgery, unresectable tumor, progression or recurrence according to the Response Evaluation Criteria in Solid Tumors, version 1.1, by the investigator's assessment, or death from any cause	IASLC 2020
Provencio 2023	Progression-free survival was defined as the time from randomization to progression of disease, recurrence of disease, or death from any cause	NA
Heymach 2023	Event-free survival was defined as the time from randomization to the earliest of the following: progressive disease that precluded surgery, progressive disease that was discovered and reported by the investigator when attempting surgery and that prevented completion of the surgery, local or distant recurrence assessed independently according to RECIST, or death from any cause.	IASLC 2020
Lu 2023	EFS is defined as the time from randomization until radiographic disease progression, local progression precluding surgery, inability to resect the tumor, local or distant recurrence, or death due to any cause.	NA
Cascone 2023	NA	irPRC
Lei 2023	EFS (defined as the time from randomization to the first occurrence of disease progression or recurrence, or death from any cause	NA
Yue 2023	NA	NA

© 2024 Sorin M et al. *JAMA Oncology.*

eTable 7. Reasons precluding surgery.

First author	ITT Chemo IO	ITT Chemo	Did not receive surgery, n (%) Chemo IO	Did not receive surgery, n (%) Chemo	Disease progression, n(%) Chemo IO	Disease progression, n(%) Chemo	Patient refusal, n(%) Chemo IO	Patient refusal, n(%) Chemo	Adverse event, n(%) Chemo IO	Adverse event, n(%) Chemo	Other, n (%) Chemo IO	Other, n (%) Chemo
Forde 2022	179	179	30 (16.8)	44 (24.6)	12 (6.7)	17 (9.5)	9 (5.0)	11 (6.1)	2 (1.1)	1 (0.6)	4 (2.2)	7 (3.9)
Wakelee 2023	397	400	72 (18.1)	83 (20.8)	10 (2.5)	17 (4.3)	4 (1.0)	6 (1.5)	8 (2.0)	1 (0.3)	1 (0.3)	4 (1.0)
Heymach 2023	366	374	71 (19.4)	72 (19.3)	27 (7.4)	30 (8.0)	12 (3.3)	13 (3.5)	7 (1.9)	5 (1.3)	22 (6.0)	23 (6.1)
Provencio 2023	57	29	4 (7.0)	9 (31.0)	0 (0)	4 (13.8)	1 (1.8)	0 (0)	1 (1.8)	1 (1.8)	2 (3.5)	4 (13.8)
Lu 2023	202	202	36 (17.8)	54 (26.7)	5 (2.5)	31 (15.3)	18 (8.9)	13 (6.4)	6 (3.0)	0 (0)	7 (3.5)	10 (5.0)
Cascone 2023	229	232	51 (22.3)	54 (23.3)	13 (5.7)	22 (9.5)	11 (4.8)	18 (7.8)	7 (3.1)	4 (1.7)	15 (6.6)	16 (6.9)
Lei 2023	47	47	7 (14.9)	5 (2.1)	0 (0)	1 (2.1)	2 (4.3)	2 (4.3)	0 (0)	0 (0)	0 (0)	0 (0)
Yue 2023	226	227	36 (15.9)	54 (23.8)	5 (2.2)	12 (5.3)	20 (8.8)	28 (12.3)	6 (2.7)	2 (0.9)	5 (2.2)	12 (5.3)

© 2024 Sorin M et al. *JAMA Oncology.*
