# Supplementary material for: Neoadjuvant Chemoimmunotherapy for NSCLC: A Systematic Review and Meta-Analysis
Source: JAMA Oncol. 2024 Mar 21;10(5):621–33. doi: 10.1001/jamaoncol.2024.0057 (PMC10958389; doi:10.1001/jamaoncol.2024.0057)
Supplement: Supplement 2. — Data Sharing Statement [file jamaoncol-e240057-s002.pdf]

Data Sharing Statement

Sorin. Neoadjuvant Chemoimmunotherapy for NSCLC: A Systematic Review and Meta-Analysis. *JAMA Oncol*. Published online March 21, 2024. doi:10.1001/jamaoncol.2024.0057

Data
Data available: No

Additional Information
Explanation for why data not available: No patient-level data was used.
